# Computational understanding of catalyst-controlled borylation of fluoroarenes: directed *vs.* undirected pathway†

**DOI:** 10.1039/d0ra03428b

**Published:** 2020-05-21

**Authors:** Yu-hua Liu, Zhong-Jie Jiang

**Affiliations:** School of Physics and Electronic Engineering, Guangzhou University Guangzhou 510006 China; Guangzhou Key Laboratory for Surface Chemistry of Energy Materials, New Energy Research Institute, College of Environment and Energy, South China University of Technology Guangzhou 510006 Guangdong China eszjiang@scut.edu.cn

## Abstract

In this work, density functional theory (DFT) calculations are performed to understand the origin of the regioselective C–H borylation of aromatics catalyzed by Co(i)/^iPr^PNP and Ir(iii)/dtbpy (4,4-di-*tert*-butyl bipyridine). The calculation results indicate that for the Co(i)/^iPr^PNP catalytic system, the undirected pathway is 2.9 kcal mol^−1^ more favoured over the directed pathway leading to *ortho*-to-fluorine selectivity. In contrast, for the Ir(iii)/dtbpy catalytic system, the directed pathway is 1.2 kcal mol^−1^ more favoured over the undirected pathway bringing about *ortho*-to-silyl selectivity. For Co(i)/^iPr^PNP catalyzed borylation, the undirected pathway which involves steps of *ortho*-to-fluorine C–H oxidative addition, C–B reductive elimination, B–B oxidative addition, and B–H reductive elimination is favorable due to the electron deficient character of the *ortho*-to-fluorine C–H bond. For Ir(iii)/dtbpy catalyzed borylation, the directed pathway consisting of Si–H oxidative addition, B–H reductive elimination, C–H oxidative addition, B–B oxidative addition, C–B reductive elimination, Si–H reductive elimination is favored over the undirected pathway attributed to the directing effect of the hydrosilyl group. The favourable undirected pathway (*orth*o-to-fluorine selectivity) for Co(i)/^iPr^PNP catalyzed borylation and the favourable directed pathway (*ortho*-to-silyl selectivity) for Ir(iii)/dtbpy catalyzed borylation could explain well the experimentally observed *ortho*-to-fluorine borylation of hydrosilyl substituted fluoroarenes with cobalt catalyst (J. V. Obligacion, M. J. Bezdek and P. J. Chirik, *J. Am. Chem. Soc.*, 2017, **139**, 2825–2832) and *ortho*-to-silyl selectivity with iridium catalyst (T. A. Boebel and J. F. Hartwig, *J. Am. Chem. Soc.*, 2008, **130**, 7534–7535).

## Abstract 2

In this work, density functional theory (DFT) calculations are performed to understand the origin of the regioselective C–H borylation of aromatics catalyzed by Co(i)/^iPr^PNP and Ir(iii)/dtbpy (4,4-di-*tert*-butyl bipyridine).

## Graphical abstract

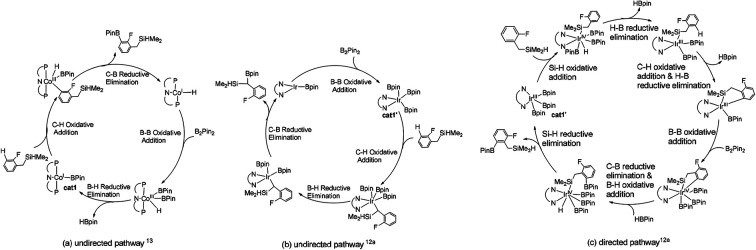

## Introduction

Transition metal catalyzed C–H borylation of aromatics has attracted considerable attention as it offers an alternative method to standard organic synthesis.^1^ However, different C–H bonds in reactants can result in different functionalization, leading to the formation of the resulting products with substantially different properties.^2^ Therefore, controlling the regioselectivity of the C–H borylation reaction is of great importance to obtain the desired synthetics. Generally, strategies for the regioselective C–H borylation of aromatics include the undirected reaction pathway, in which no directing groups are involved in the reaction,^3^ and directing group controlled regioselective borylation,^3*f*–*j*^ where the reaction regioselectivity is well controlled by a directing group.

**Scheme 1:**
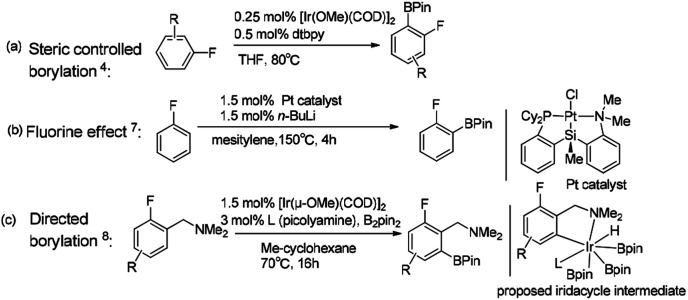
Complementary selectivity in transition metal catalyzed C(sp2)–H borylation of fluoroarenes.^5,7,8^

**Scheme 2:**
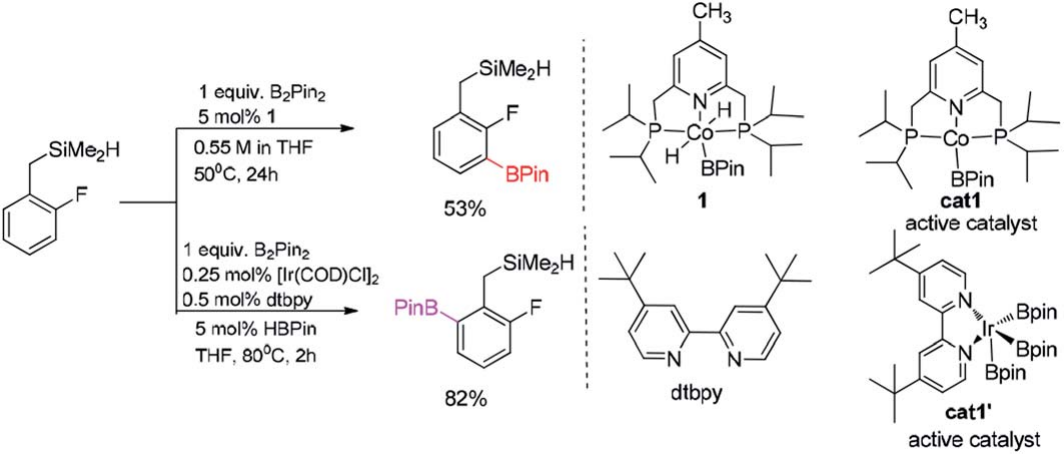
Computational model for borylation of hydrosilyl substituted fluoroarenes.^11^

Hartwig reported that the *ortho*-to-fluorine selectivity was controlled by steric hindrance in iridium/dtbpy catalyzed borylation of trisubstituted fluoroarenes^4^ (Scheme 1a) in which the product of borylated fluoroarene is especially essential for pharmaceutical chemistry.^5^ In contrast, Chatani reported platinum-NHC catalyzed C–H borylation of fluoroarenes, which afforded *ortho*-to-fluorine arylboronates with no steric protection.^6^ Similarly, Iwasawa reported the *ortho*-to-fluorine effect in platinum/PSiN catalyzed C–H borylation of arenes^7^ (Scheme 1b). Different from the steric controlled borylation by installing large group on the substrate or catalyst, introducing directing group bearing heteroatoms (*e.g.* N, O, S, Si) on the substrate is an effective way to achieve *meta*/*para*-to-fluorine C–H borylation.^8–10^ The interactions between directing groups and catalyst could bring one of the *ortho*-C–H bonds close to the metal center and promote its regioselective activation.^9*a*,*b*^ For example, Iwasawa reported the iridium-picolylamine catalyzed *ortho*-to-amine borylation of fluoroarenes using dimethylamine as the directing group^8^ (Scheme 1c). Hartwig *et al.* reported the hydrosilyl directed borylation of fluoroarenes catalyzed by iridium/dtbpy favoring *ortho*-to-silyl selectivity^10^ (Scheme 2). In contrast, Obligacion *et al.* reported the same reaction catalyzed by cobalt/^iPr^PNP with *ortho*-to-fluorine selectivity^11^ (Scheme 2).

The examples mentioned above clearly demonstrate that the C–H borylation of aromatics can proceed by the undirected or directed pathways with the iridium or cobalt-based catalysts.^12,13^ Recently, Hall's group had reported the cobalt pincer complex catalyzed regioselective borylation of aromatics^13*b*^ (without directing group) while Sunoj's group reported the use of hydrosilyl (–SiR_2_H) as a directing group in the iridium-catalyzed regioselective borylation of the benzylic C–H bond.^9*a*^ Although there have been some reports on the Si–Co and Si–Ir metal complex,^3*l*,9*a*,12*d*^ and the directing effect of the silyl group has been identified in iridium catalyzed borylation of benzylic C–H bond,^9*a*^ detailed mechanism study on the regioselectivity of the borylation reactions in Scheme 2 has not been reported. In the iridium and cobalt catalyzed borylation of hydrosilyl substituted fluoroarenes (Scheme 2), how about directing effect of hydrosilyl group for both the cobalt and the iridium catalyzed borylation? Is the directing effect always favourable? Based on these considerations, we studied the detailed mechanism of borylation of hydrosilyl substituted fluoroarenes catalyzed by iridium and cobalt by density functional theory (DFT) method. Specifically, our attention mainly focuses on the questions raised by experimental observations: (1) what are the details of the reaction pathways? (2) which step is the rate-determining step?

## Results and discussion

We choose the reaction between hydrosilyl substituted fluoroarenes and B_2_pin_2_ as the model (Scheme 2). Activations of both *ortho*-to-fluorine C–H bond and *ortho*-to-silyl C–H bond by the cobalt catalyst 1 and Ir(iii)/dtbpy are calculated to make comparison and explain the regiochemical preference (Scheme 2). Since the work by Patel *et al.*^12*a*^ and Obligacion *et al.*^13^ have suggested that the most likely active catalysts for Co(i) and Ir(iii) catalyzed borylation reactions are cat1 and cat1′, respectively, we discuss all the reaction pathways in the following sections with cat1 and cat1′ as the starting points, given that a cascade of steps along the reaction pathways is triggered by the active catalysts.

**Fig. 1:**
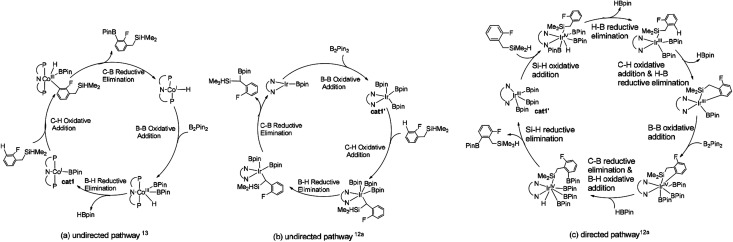
Potential mechanisms of (a) directed pathway for borylation of aromatic C–H bond^13^ and (b) undirected pathway for borylation of Si–H bond^12*a*^ (c) directed pathway for borylation of benzylic C–H bond.^12*a*^

According to the probable mechanisms of Ir(iii) and Co(i) catalyzed borylation of aromatics proposed in literature,^12*a*,13^ the directing group controlled borylation catalyzed by iridium may proceed in a way that the iridium centre is brought close to the *ortho*-to-silyl C–H bond by the silyl group and induces the activation of *ortho*-to-silyl C–H bond to form a 5-membered cycle.^12*a*^ In contrast, cobalt-catalyzed borylation starts with *ortho*-to-fluorine C–H oxidative addition^13^ and no participation of directing group. The adaption of reported mechanisms to our peculiar reaction system is shown in Fig. 1. The proposed pathways consist of the directed and undirected pathway. For undirected pathway (Fig. 1a), the cobalt catalyst 1 triggers a cascade of significant steps which involves: (i) B–B oxidation addition, (ii) H–B reductive elimination, (iii) C–H oxidation addition, and (iv) C–B reductive elimination. Alternatively, the undirected pathway may refer to the activation of the benzylic C–H bond which involves (Fig. 1b): (i) C–H oxidation addition, (ii) H–B reductive elimination, (iii) C–B reductive elimination, and (iv) B–B oxidation addition. While, directed pathway catalyzed by iridium is divided into six distinct steps (Fig. 1c): (i) Si–H oxidation addition to Ir center; (ii) H–B reductive elimination; (iii) C–H bond oxidation addition & H–B reductive elimination; (iv) B–B oxidation addition; (v) C–B bond formation; (v) H–B addition; (vi) Si–H elimination. There is also a possibility that both the directed and undirected pathways co-exist for the cobalt/iridium-catalyzed C–H activation process. Significant differences exist between the three catalytic cycles. The preference for which pathway depends on the nature of the catalyst such as the numbers of open coordination sites on the transition metal centre of catalyst and electronic effects of the catalyst. In the following section, we use the DFT method to calculate both directed and undirected C–H activation pathways to identify the energetically most favored pathway, with the aim of revealing the details of the mechanism.

### Co(i)/^iPr^PNP catalyzed undirected and directed pathway

**Fig. 2:**
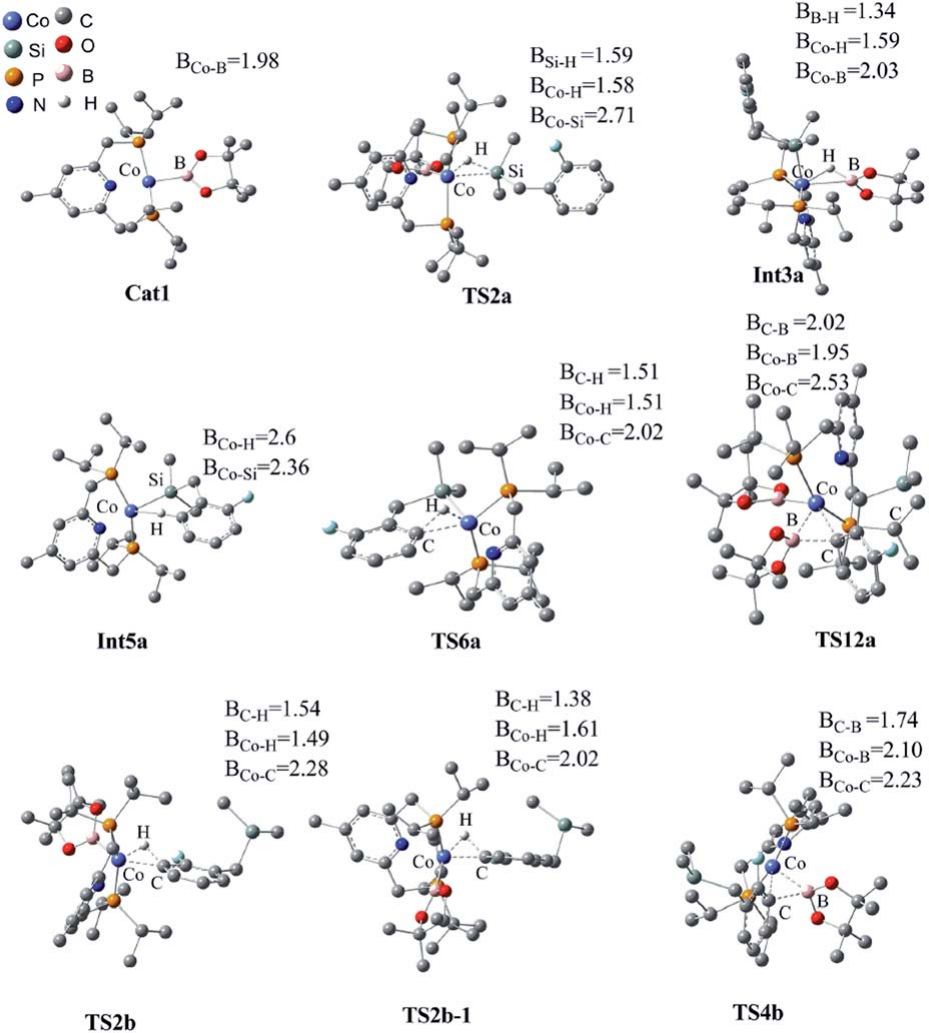
Optimized structures of the key transition states and intermediates in Co(i)/^iPr^PNP catalyzed borylation of silyl substituted fluoroarenes, along with the key bond distances in angstroms. Color code, C: dark gray, O: red, B: pink, H: light gray, N: dark blue, Co: light blue, Si: celeste, P: yellow. Irrelevant hydrogen atoms are omitted for clarity.

**Fig. 3:**
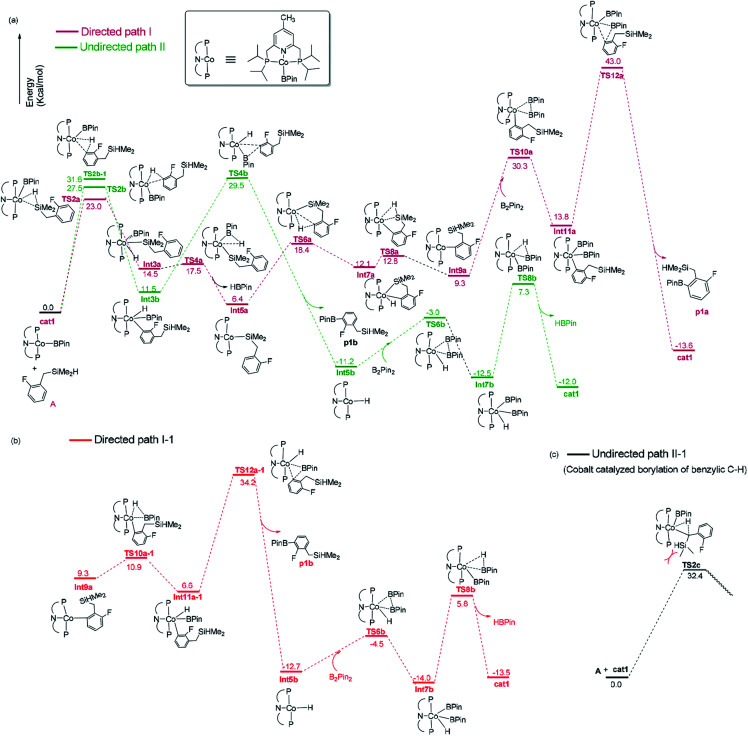
Gibbs free energy profile of directed path I and path I-1, undirected path II and path II-1 in the borylation of hydrosilyl substituted fluoroarenes catalyzed by Co(i)/^iPr^PNP.

We have examined the geometric and energy features of each elementary step involved in the Co(i)/^iPr^PNP catalyzed directed and undirected pathways. The optimized structures of selected transition states (TS) and intermediates are given in Fig. 2, while the energy profiles are presented in Fig. 3. All the TS structures and Co(i) species studied show the tridentate coordination of the pincer ligand. The Co–N distance of cobalt pincer-complex cat1 (Fig. 2) is rather short, 2.00 Å.

The directed pathway starts from silylation (Fig. 3a, dull red). The hydrogen of hydrosilyl group approaches cobalt centre from the position adjacent to boryl group affording Int3a*via*TS2a with a barrier of 23.0 kcal mol^−1^ (Fig. 3a). The comparison of geometry between TS2a and cat1 indicates that Si–H bond approaches to the axial position of the metal center, pushes the equatorial boryl ligands to the axial position (Fig. 2).^14^ The distance between the incoming hydride and the boron atom Int3a is 1.34 Å (Fig. 2) indicating a weak orbital interaction between the vacant p-orbitals of the boron and the hydride.^15,16^ It is expected that Int3a first converts to Int5a to release vacant coordination sites *via* expulsion of a molecule of HBpin. The required energy for the removal of a molecular of HBpin from Int3a is only 3.0 kcal mol^−1^. The *ortho*-to-silyl aromatic C–H bond in Int5a is brought close to the cobalt center by silyl group, such that C–H could oxidatively add to cobalt center affording Int7a*via* a cyclic transition state TS6a (Fig. 2). At first glance, there is a possibility that B_2_pin_2_ oxidatively adds to the cobalt center of Int7a. However, attempts to find a corresponding TS is failed, presumably resulting from the unavailable coordination sites on the cobalt center. Subsequently, we investigate the possibility of Si–H bond reductive elimination from Int7a. The reductive elimination of Si–H bond from Int7a affords Int9a*via*TS8a. Given that there are partially open coordination sites at the cobalt center in Int9a, B_2_pin_2_ could add to the cobalt center to form Int11a. One of the boryl ligand in Int11a approaches the adjacent aromatic carbon to form C–B bond *via*TS12a (Fig. 2). Upon the C–B bond reductive elimination, the product p1a is afforded, and active catalyst cat1 is regenerated. Along with the energetic profile of the directed pathway (Fig. 3, dull red), the overall barrier for the C–B reductive elimination *via* TS12a is the largest (43.0 kcal mol^−1^), and can be considered as the rate determining step. Starting from int9a, in place of B_2_Pin_2_ oxidative addition, HBpin is also possible to add to the cobalt center following with the C–B reductive elimination and regeneration of cat1. The corresponding energetic profile is shown in Fig. 3, which shows an overall barrier of 34.2 kcal mol^−1^ (TS12a-1 in Fig. 3b). It is noteworthy that in this path the steps of the catalyst regeneration is the same as that in undirected path II (Fig. 3a, Int5b → cat1) and the steps before Int9a are the same as those in the directed path I (Fig. 3a, cat1 → Int9a).

Different from the directed pathway, the undirected pathway starts with the *ortho*-to-fluorine C–H activation. In this step, the *ortho*-to-fluorine hydrogen may approach cobalt center from the position adjacent to boryl group or from the location opposite to the boryl group to afford TS2b-1 and TS2b which requires an energy of 31.6 kcal mol^−1^ and 27.5 kcal mol^−1^, respectively. For the former approach, the carbon and boryl group is in an opposite position which is impossible for the subsequent C–B reductive elimination to afford the borylated product. So, we do not calculate the pathway along with the former. The intermediate Int3b is obtained *via*TS2b. The incoming hydride and boryl group of Int3b are situated in the equatorial position (Fig. 2). Then, the aromatic C–B is reductively eliminated from Int3b*via*TS4b, affording the product p1b. The overall barrier for the C–B reductive elimination is the largest (29.5 kcal mol^−1^) and is the rate-determining step (Fig. 3a). The catalyst cat1 is regenerated upon the following B–B oxidative addition and B–H reductive elimination, which require an energy of 8.2 kcal mol^−1^ and 19.8 kcal mol^−1^, respectively. In addition, we have calculated the pathway for Co(i)-catalyzed benzylic C–H borylation (Fig. 3c). As a result of the steric hindrance between the ligand and the silyl group, the barrier for the benzylic C–H activation (32.4 kcal mol^−1^) is too high to occur under the experimental condition (50 °C, 24 h). This calculation result is different from the hydrosilyl directed borylation of *ortho*-hydrosilyl benzene in which the borylation of the benzylic C–H is favorable over the aromatic C–H bond activation.^9*a*^

Obviously, the overall barrier in the undirect pathway (29.5 kcal mol^−1^, TS4b in Fig. 3a) for Co(i)/^iPr^PNP system is much lower than that of the direct pathway (43.0 kcal mol^−1^ barrier for TS12a in Fig. 3a or 34.2 kcal mol^−1^ barrier for TS12a-1 in Fig. 3b) We believe that the lower overall barrier of the undirected pathway than directed pathway can be ascribed to the electron-withdrawing of the fluorine atom *ortho* to the activated C–H bond, because the electron deficiency property of the transition state accelerates the C–B reductive elimination (18.0 kcal mol^−1^ of the barrier for TS4b*vs.* 29.2 kcal mol^−1^ of that for TS4a, Fig. 3a). Such a high barrier difference between the directed and undirected pathway (29.5 kcal mol^−1^ overall barrier for TS4b in the undirected pathway *vs.* 43.0 kcal mol^−1^ barrier for TS12a or 34.2 kcal mol^−1^ barrier for TS12a-1 in the directed pathway, Fig. 3) suggests that the Co(i)/^iPr^PNP catalyzed borylation favors the undirect pathway. With Co(i)/^iPr^PNP catalytic system, the calculated favorable undirect pathway (*ortho*-to-fluorine selectivity) is well consistent with the experimental observed *ortho*-to-fluorine regioselectivity which is reported in the literature^11^ (53%, Scheme 2), strongly suggesting the accuracy of our calculation. Additionally, the slightly high barrier of undirected pathway (29.5 kcal mol^−1^) also well explains the relatively low chemical yield in the cobalt catalyzed model reaction (53%).^12^ It is worth noting that a recent computational study has revealed that the cobalt–carbon bonds of the intermediate in the (^iPr^PNP)Co-catalyzed borylation could be strengthened by the *ortho*-fluorine atom, which gives an additional demonstration of the *ortho*-to-fluorine regioselectivity other than *para*- or *meta*-selectivity.^13*g*^

### Ir(iii) catalyzed undirected and directed pathway

**Fig. 4:**
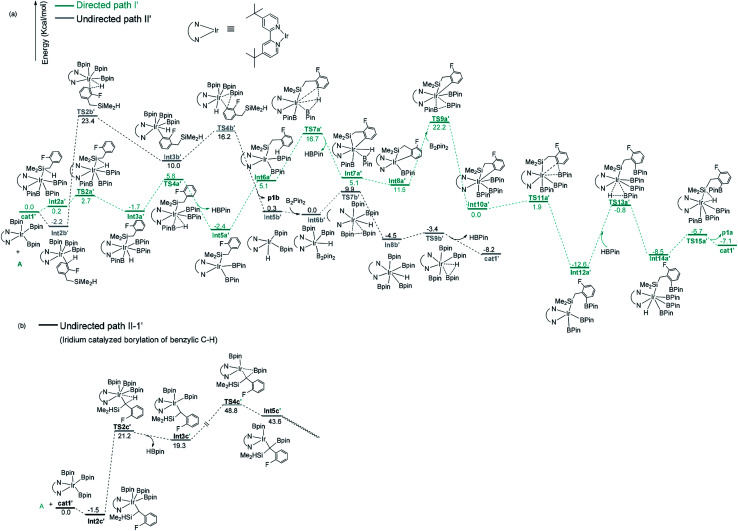
Gibbs free energy profile of path I′ and path II′ in borylation of silyl substituted fluoroarenes with Ir(iii)/dtbpy system.

**Fig. 5:**
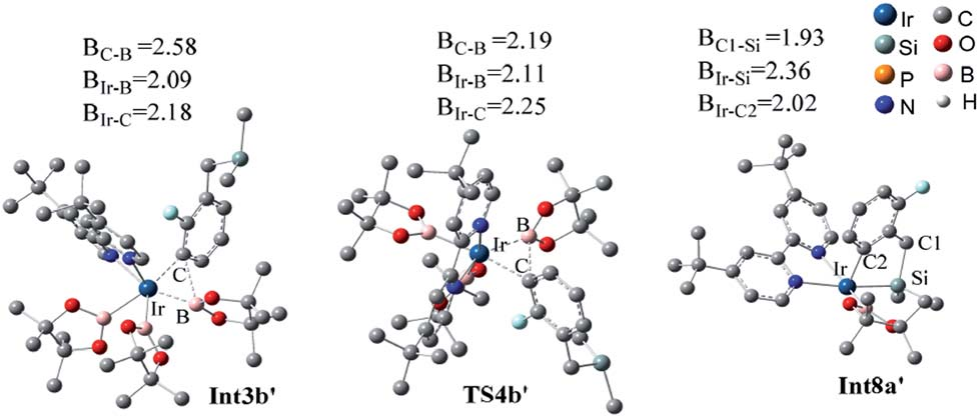
Optimized structures of the key transition states and intermediates in Co(i)/^iPr^PNP catalyzed borylation of silyl substituted fluoroarenes, along with the key bond distances in angstroms. color code, C: dark gray, O: red, B: pink, H: light gray N: dark blue, Co: light blue, Si: celeste, P: yellow. Irrelevant hydrogen atoms are omitted for clarity.

After a brief study on the directed and undirected pathways catalyzed by Co(i)/^iPr^PNP, we turn to the similar pathways catalyzed by Ir(iii)/dtbpy catalytic system. The energy and geometric features of each elementary step involved in the Ir(iii)/dtbpy catalyzed directed and undirected pathways are shown in Fig. 4 and 5.

For the undirected path I′, the catalytic cycle starts with *ortho*-to-fluorine C–H activation of the substrate A to form Int3b′*via*TS2b′ (a barrier of 25.6 kcal mol^−1^, Fig. 4a). The energy required for the subsequent C–B reductive elimination is 6.2 kcal mol^−1^ (TS4b′, Fig. 4a). After C–B reductive elimination, the intermediate Int6b′ is formed and coordinates with B_2_pin_2_*via*TS7b′, which requires an energy of 9.9 kcal mol^−1^, a molecular HBpin is released *via*TS9b′, which require an energy of 1.1 kcal mol^−1^, and finally, the catalytic cycle is closed by the regeneration of the catalyst cat1′. The most stable intermediate in undirected path I′ is Int2b′. The intermediate Int5a′ in the directed path II′ is 0.2 kcal mol^−1^ more stable than Int2b′, and the “population” of Int2b′ will be marginally smaller than the population of Int5a′. The reaction may proceed from the intermediate Int5a′. Therefore, in a strict sense, the overall energy barrier within C–H approximation should be calculated as the energy difference between TS2b′ and Int5a′(25.8 kcal mol^−1^, Fig. 4a).

It is worth noting that the barrier for the initial *ortho*-to-fluorine C–H activation catalyzed by Ir(iii)/dtbpy is lower than that catalyzed by Co(i)/^iPr^PNP (25.8 kcal mol^−1^*vs.* 27.5 kcal mol^−1^) (TS2b′ in Fig. 4a*vs.*TS2b in Fig. 3a), which can be ascribed to the electron effects on the transition states of C–H activation. Comparing the transition states of the *ortho*-to-fluorine C–H activation in cobalt and iridium catalyzed borylation (TS2b, TS2b′), the Mulliken charge on the cobalt center of TS2b is negative while that on iridium of TS2b′ is positive (−0.517325 *vs.* 0.349631, Fig. 1, ESI†), which shows stronger electron withdrawing effect of iridium center than that of cobalt center. We propose that the electron withdrawing character of iridium center could facilitate the *ortho*-to-fluorine C–H activation, which may explain for the lower barrier of TS2b′ (Fig. 4a) than that of TS2b (Fig. 3a) (25.8 kcal mol^−1^*vs.* 27.5 kcal mol^−1^).

Additionally, as for iridium catalyzed directed path I′, the active catalyst cat1′ triggers the catalytic cycle by silylation, which only requires an energy of 2.7 kcal mol^−1^ (TS2a′). Given that the formed Int3a′ is saturated coordination,^13*a*^ it is expected to exclude one molecular of HBpin prior to the C–H oxidative insertion. Upon release of one molecular HBpin, the following C–H oxidative insertion is accompanied by the release of another molecular HBpin which can be confirmed by IRC calculations. Then the intermediate Int8a′ is afforded, which has open coordination sites. One equivalent of B_2_pin_2_ thereafter oxidatively add to Int8a′, generating Int10a′*via*TS9a′, with an overall barrier of 24.6 kcal mol^−1^. In the intermediate Int10a′, one boryl group is situated close to the *ortho*-to-silyl aromatic carbon (Fig. 4a). Then, C–B bond is formed *via* C–B reductive elimination from Int10a′, affording Int12a′*via*TS11a′. Because intermediate Int12a′ has open coordination sites, one molecular HBpin is added to iridium center of Int12a′ to generate Int14a′*via*TS13a′. The added HBpin provides hydrogen for the subsequent reductive elimination of the Si–H bond. Upon Si–H reductive elimination, the catalytic cycle is closed by affording the final product and regeneration of catalyst cat1′.

In addition, we have calculated the pathway for Ir-catalyzed benzylic C–H borylation (Fig. 4b). The barrier for path II-1′ is much higher (48.8 kcal mol^−1^) than other pathways (25.8 kcal mol^−1^ for Ir catalyzed undirected path II′; 24.6 kcal mol^−1^ for Ir catalyzed directed path I′) and should be finally ruled out. Similar to Co(i)-catalyzed benzylic C–H borylation, it is different from the hydrosilyl directed borylation of *ortho*-hydrosilyl benzene in which the borylation of the benzylic C–H bond is favorable over the borylation of the aromatic C–H bond.^9*a*^ Comparing the rate-determining step in the directed path I′ and undirected path II′ for the iridium catalyzed C–H borylation (TS9a′*vs.*TS2b′), the directed pathway leading to *ortho*-to-silyl selectivity is 1.2 kcal mol^−1^ more favorable over undirected pathway leading to *ortho*-to-fluorine selectivity (24.6 kcal mol^−1^ barrier of TS9a′ in Fig. 4a*vs.* 25.8 kcal mol^−1^ barrier of TS2b′ in Fig. 4a).With Ir(iii)/dtbpy catalytic system, the calculated favorable directed pathway (*ortho*-to-silyl selectivity) is also consistent with experimental observed *ortho*-to-silyl selectivity^11^ (82%, Scheme 2).

## Conclusions

The mechanisms of regioselective borylation of hydrosilyl substituted fluoroarenes by Ir(iii)/dtbpy and Co(i)/^iPr^PNP catalysts have been studied by DFT calculation. It shows that the borylation of hydrosilyl substituted fluoroarenes by Ir(iii)/dtbpy exhibits an *ortho*-to-hydrosilyl selectivity and follows a directed reaction pathway consisting of steps: (i) Si–H oxidative addition (ii) H–B reductive elimination, (iii) C–H bond oxidative addition and H–B reductive elimination, (iv) B–B oxidative addition, (v) C–B formation, (vi) H–B oxidative addition and (vi) Si–H reductive elimination. The preference for *ortho*-to-hydrosilyl selectivity in Ir(iii)/dtbpy catalyzed borylation can be attributed to the directing effect of hydrosilyl group. The borylation of hydrosilyl substituted fluoroarenes by Co(i)/^iPr^PNP, however, exhibits a high selectivity of *ortho*-to-fluorine and follows the undirected pathway which involving the steps: (i) C–H oxidative addition, (ii) C–B reductive elimination, (iii) B–B oxidative addition, and (iv) B–H reductive elimination. The preference for *ortho*-to-fluorine selectivity in Co(i)/^iPr^PNP catalyzed borylation is attributable to the acidity of *ortho*-to-fluorine C–H bond, while in the silyl-directed pathway, the high steric hindrance in the transition state of C–B reductive elimination leads to too high overall barrier to overcome. These calculation results well explain the experimental observation reported previously, strongly suggesting the accuracy of our calculation. The work present here therefore provides rational mechanistic insights into the origin of regioselective borylation of hydrosilyl substituted fluoroarenes. This will be helpful to well understand the underlying physics of the regioselective borylation of hydrosilyl substituted fluoroarenes and can be extended to the synthesis of other organic compounds with predictable regioselectivity by introducing suitable directing group, adjusting the available coordination sites on the catalyst, and/or modifying the electronic effects of the catalyst.

## Computational section

Geometry optimizations without symmetry restriction were carried out at the B3LYP^17^/BSI level, where BSI denotes the combination of the lanl2dz^18^ for Ir and Co, 6-31G(d)^19^ basis for other atoms. Frequency results were examined to confirm stationary points as transition states (only one imaginary frequency) or minima (no imaginary frequencies), and were also used to obtain zero-point energy-corrected enthalpies and free energies at 298.15 K and 1 atm. In addition, intrinsic reaction coordinate (IRC) analysis was conducted to confirm that the transition state connects the correct reactant and product on the potential energy surface.^20^ The energetic results were further improved by single-point energy calculations at ωB97XD^21*a*^/BSII level of theory, where BSII represents a basis set with SDD^22^ for Ir and Co and Def2TZVP^21*b*^ basis set for other atoms. The solvent effects accounted by the SMD^23^ solvation model, using the experimental solvent tetrahydrofuran. Furthermore, natural population analysis (NPA)^24^ was performed at the same level by single point calculations. The Gibbs free energies obtained from the ωB97XD/BSII//B3LYP/BS1 level was discussed in this study, unless otherwise specified. All calculations are performed with the Gaussian 09 program package.^25^

## Conflicts of interest

There are no conflicts to declare.

## Supplementary Material

RA-010-D0RA03428B-s001
