# Supplementary material for: Computational understanding of catalyst-controlled borylation of fluoroarenes: directed vs. undirected pathway
Source: RSC Adv. 2020 May 21;10(33):19562–9. doi: 10.1039/d0ra03428b (PMC9054080; doi:10.1039/d0ra03428b)
Supplement: RA-010-D0RA03428B-s001 [file RA-010-D0RA03428B-s001.pdf]

Electronic Supplementary Material (ESI) for RSC Advances.
This journal is © The Royal Society of Chemistry 2020

Computational understanding on catalyst-controlled borylation of fluoroarenes: directed vs. undirected pathway

Yuhua Liu,[a] Zhong-Jie Jiang*[b]

[a] *School of physics and electronic engineering, Guangzhou University, Guangzhou, 510006, China*

[b] *Guangzhou Key Laboratory for Surface Chemistry of Energy Materials, New Energy Research Institute, College of Environment and Energy, South China University of Technology, Guangzhou 510006, Guangdong, China*

I. Geometry and Mulliken charge for selected transition states at the B3LYP/6-31G* Level

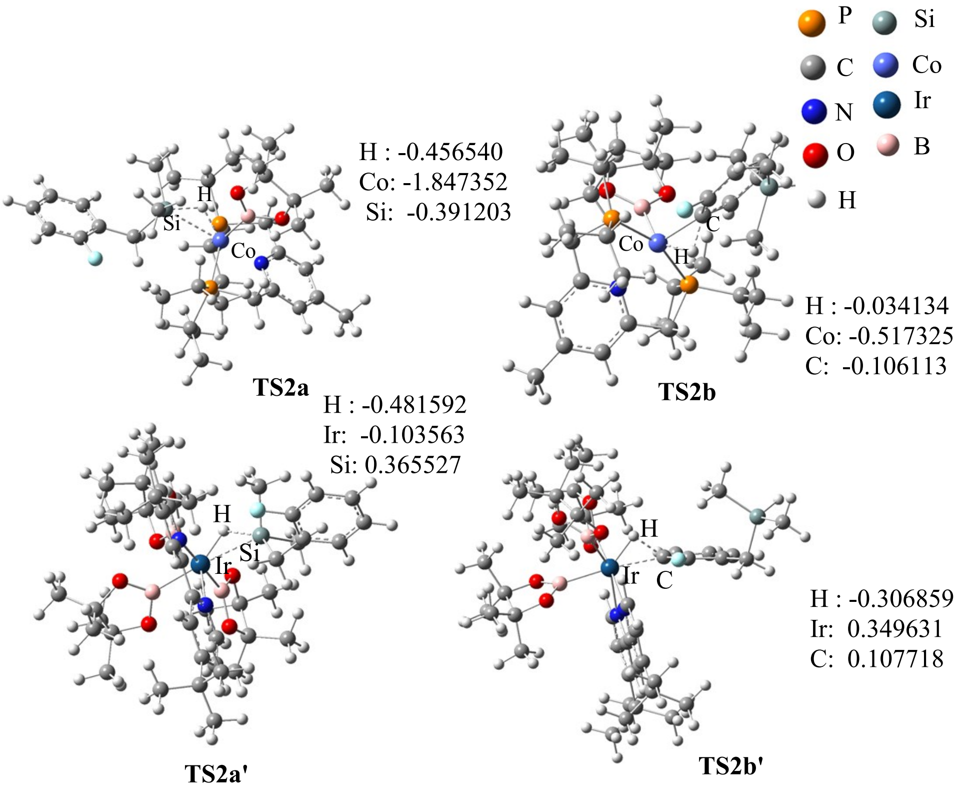

Fig 1. Mulliken Charge Distribution for selective transition states at the B3LYP/6-31G* Level

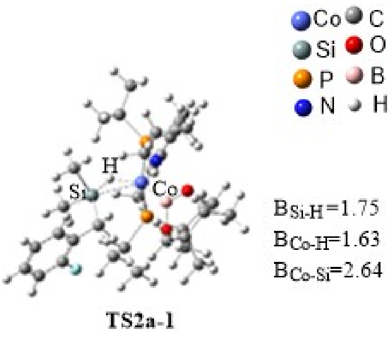

Fig 2. Geometry for selected **TS2a-1**

II. Cartesian coordinates and energies from the B3LYP method and electronic energies from the M06 method.

cat1

Number of Negative Frequencies = 0

B	-0.20341	1.07238	-2.01027
C	0.70121	0.24491	2.62379
C	0.64001	0.0035	3.99461
C	-0.58135	-0.28458	4.61216
C	-1.719	-0.25819	3.79939
C	-1.61062	0.01096	2.43583
C	-0.91861	1.78218	-4.11942
C	0.59791	2.14837	-3.93184
N	-0.40937	0.20817	1.83662
O	-1.15136	0.8636	-3.02614
O	0.78077	1.95073	-2.50941
C	1.55054	1.19061	-4.66306
H	2.57748	1.40058	-4.34597
H	1.33216	0.14912	-4.41803
H	1.49702	1.3196	-5.75018
C	0.96728	3.59381	-4.27302
H	2.03244	3.75616	-4.07507
H	0.78841	3.80726	-5.33384
H	0.40142	4.31146	-3.67409
C	-1.25702	1.07424	-5.43285
H	-2.32636	0.83896	-5.46163
H	-1.03076	1.71318	-6.29509
H	-0.70326	0.13842	-5.53702
C	-1.85826	2.98337	-3.92223
H	-1.76702	3.71123	-4.73632
H	-2.89378	2.62961	-3.89912
H	-1.65324	3.49377	-2.97572
H	1.5563	0.0375	4.57756
H	-2.70365	-0.41992	4.22947
C	-2.82012	0.22133	1.57181
C	1.98187	0.60428	1.92275
H	2.69391	1.06632	2.61481
C	-0.6669	-0.60582	6.08258
H	-0.51457	-1.6795	6.25633
H	0.09971	-0.07263	6.65462
H	-1.64727	-0.34447	6.49401
Co	-0.22123	0.32337	-0.17683
H	2.44475	-0.30263	1.51158
P	1.49584	1.6244	0.42558
C	0.95653	3.29364	1.15933
H	-0.09265	3.08293	1.39924
C	0.95974	4.38638	0.07691
H	0.429	5.2761	0.43938
H	1.97815	4.69758	-0.18354
H	0.46968	4.03979	-0.83799
C	1.63456	3.7671	2.45585
H	2.70218	3.96924	2.32934
H	1.16481	4.70057	2.79426
H	1.52273	3.03742	3.26468
C	3.12659	1.93636	-0.46061
H	2.82948	2.58417	-1.29145
C	4.22137	2.638	0.35912
H	5.13028	2.7323	-0.24974
H	3.93476	3.6453	0.67237
H	4.49323	2.06782	1.25582
C	3.66192	0.63125	-1.06845
H	4.54707	0.8476	-1.68069
H	3.97363	-0.07857	-0.29214
H	2.92098	0.14203	-1.70081
H	-3.06188	1.29162	1.62169
H	-3.68895	-0.31689	1.96308
P	-2.43828	-0.13139	-0.22986
C	-2.90152	-1.96973	-0.33708
C	-3.8082	0.85883	-1.11498
C	-5.07389	1.16344	-0.29258
H	-4.86709	1.73278	0.61865
H	-5.76063	1.76884	-0.89887
H	-5.61067	0.25237	-0.0072
C	-2.50612	-2.59806	-1.68449
H	-3.19138	-2.30787	-2.4862
H	-1.49365	-2.31078	-1.97621
H	-2.53512	-3.69224	-1.60382
C	-4.20713	0.26252	-2.47619
H	-4.82165	-0.63616	-2.35945
H	-4.81248	0.99486	-3.0273
H	-3.3319	0.02184	-3.07996
H	-3.29477	1.80661	-1.31641
C	-4.33319	-2.36869	0.05774

H	-5.06978	-2.03747	-0.68165
H	-4.40652	-3.46276	0.11858
H	-4.63423	-1.97009	1.03266
H	-2.21749	-2.38899	0.41407

Thermal correction to Energy=0.761716

Thermal correction to Enthalpy= 0.762660

Thermal correction to Gibbs Free Energy= 0.648493

Sum of electronic and zero-point Energies= -2077.504616

Sum of electronic and thermal Energies= -2077.463224

Sum of electronic and thermal Enthalpies= -2077.462280

Sum of electronic and thermal Free Energies=-2077.576446

SCF Done: E(wB97XD) = -2079.153339

A

Number of Negative Frequencies = 0

C	-1.35231	-0.04842	-1.25354
C	-1.86705	0.89241	-2.16878
C	-2.37912	-0.69374	-0.50463
C	-3.20243	1.25699	-2.34396
C	-3.72695	-0.37347	-0.64101
H	-2.09901	-1.41142	0.25537
C	-4.15047	0.60301	-1.54485
H	-4.45824	-0.87817	-0.01249
H	-5.20291	0.85719	-1.64223
F	-0.97027	1.50441	-3.02281
C	-3.60596	2.27216	-3.38765
H	-4.51462	2.79449	-3.06028
H	-2.82989	3.0365	-3.51246
Si	-3.97012	1.51159	-5.10096
H	-5.18011	0.6428	-4.97238
C	-2.5308	0.45441	-5.71987
H	-1.61208	1.04496	-5.80888
H	-2.32757	-0.36799	-5.02522
H	-2.75519	0.02064	-6.70178
C	-4.36082	2.89959	-6.33349
H	-3.50087	3.56799	-6.46497
H	-4.61996	2.49459	-7.31915
H	-5.20633	3.50932	-5.99306
H	-0.30912	-0.25462	-1.13462

Thermal correction to Energy= 0.206458

Thermal correction to Enthalpy= 0.207402

Thermal correction to Gibbs Free Energy= 0.154946

Sum of electronic and zero-point Energies=-739.945598

Sum of electronic and thermal Energies= -739.933175

Sum of electronic and thermal Enthalpies= -739.932231

Sum of electronic and thermal Free Energies= -739.984688

SCF Done: E(wB97XD) = -740.196781389

TS2a

Number of Negative Frequencies = 1

Co	0.4114	0.15182	0.14931
P	-0.39993	-1.9747	-0.34919
P	1.71628	1.84279	-0.98342
N	2.19905	-0.74594	0.27258
C	1.00839	-2.83301	0.55515
H	0.74644	-2.75385	1.61676
H	1.12404	-3.89591	0.32214
C	2.29708	-2.09099	0.38063
C	3.52947	-2.74498	0.38514
H	3.55081	-3.82881	0.45933
C	4.71922	-2.01856	0.31234
C	4.60103	-0.62683	0.242
H	5.48793	-0.00057	0.19933
C	3.34539	-0.02426	0.22204
C	3.17168	1.46082	0.14039
C	-0.18989	0.68976	4.31947
C	0.9932	-0.3438	4.32131
B	0.36892	0.13822	2.11939
O	1.41587	-0.32874	2.9389
O	-0.66692	0.60133	2.95845
C	-0.47477	-2.92448	-2.01627
H	-1.45512	-2.64083	-2.4232
C	-0.40532	-4.45945	-1.97826
H	-0.4997	-4.86505	-2.99505
H	0.55533	-4.81242	-1.58498
H	-1.19989	-4.9078	-1.37565
C	0.58804	-2.36858	-2.97275
H	0.48675	-1.28555	-3.08226
H	1.60261	-2.57722	-2.61094
H	0.49166	-2.82576	-3.96645
C	-1.91357	-2.67148	0.58772
H	-2.09528	-1.88051	1.32401
C	-1.73621	-3.98355	1.37408
H	-0.96476	-3.90787	2.14578
H	-2.67688	-4.23111	1.88456
H	-1.48382	-4.83171	0.72986
C	-3.15416	-2.77255	-0.31775
H	-3.08848	-3.62787	-0.99912

H	-4.05478	-2.91274	0.29356
H	-3.30603	-1.87556	-0.92666
C	0.54324	-1.77711	4.64821
H	1.36634	-2.46518	4.4274
H	0.27377	-1.89449	5.70413
H	-0.31807	-2.06802	4.0382
C	-1.34307	0.36551	5.27099
H	-1.00512	0.35769	6.31456
H	-2.12369	1.12815	5.17661
H	-1.79326	-0.60402	5.04403
C	2.1859	0.03039	5.20332
H	1.89338	0.09601	6.25846
H	2.96359	-0.73616	5.11582
H	2.62277	0.98591	4.90334
C	0.27188	2.13909	4.54649
H	1.09621	2.39802	3.87472
H	-0.56291	2.81386	4.33069
H	0.59472	2.31223	5.57958
H	4.10768	1.95844	-0.13396
H	2.86821	1.81851	1.13197
C	2.569	1.95564	-2.6969
H	1.7493	2.25889	-3.36371
C	3.72032	2.96172	-2.86441
H	4.55179	2.74227	-2.18402
H	4.12007	2.90339	-3.8862
H	3.41271	3.99585	-2.69448
C	3.04197	0.56335	-3.14606
H	3.92235	0.24009	-2.57822
H	2.26674	-0.19361	-3.01521
H	3.32557	0.58274	-4.20647
C	1.32993	3.65645	-0.55807
H	0.5715	3.54287	0.22453
C	0.67092	4.38916	-1.74073
H	0.24801	5.34207	-1.398
H	1.39342	4.62355	-2.52943
H	-0.1429	3.81649	-2.19429
C	2.46607	4.50207	0.0445
H	3.29736	4.65272	-0.65111
H	2.07818	5.49491	0.30917
H	2.87027	4.05958	0.96015
C	6.06548	-2.69753	0.2903
H	6.44664	-2.7821	-0.73615
H	6.80696	-2.13143	0.86477
H	6.01076	-3.70919	0.70478
Si	-1.97568	1.39913	-0.09327
C	-2.29074	1.04976	-1.96484
H	-2.32724	-0.03648	-2.09843
H	-1.41373	1.40069	-2.5164
C	-2.22262	3.26824	0.19047
H	-1.64091	3.90566	-0.47906
H	-3.28043	3.51652	0.03947
H	-1.9662	3.52868	1.22465
C	-3.32194	0.62674	0.9939
H	-4.23446	1.23055	0.9129
H	-3.58213	-0.4017	0.74009
H	-2.98074	0.64723	2.03325
C	-3.54926	1.6828	-2.4978
C	-4.80626	1.06327	-2.40088
C	-3.53367	2.94131	-3.10754
C	-5.9666	1.66968	-2.88448
H	-4.86585	0.08096	-1.93825
C	-4.66745	3.57184	-3.60238
C	-5.90041	2.92755	-3.48717
H	-6.92052	1.15753	-2.79394
H	-4.56942	4.5473	-4.06811
H	-6.79806	3.406	-3.86822
F	-2.34159	3.58536	-3.23544
H	-0.54229	1.32637	0.59783

Thermal correction to Energy= 0.970931

Thermal correction to Enthalpy=0.971876

Thermal correction to Gibbs Free Energy= 0.828314

Sum of electronic and zero-point Energies=-2817.416638

Sum of electronic and thermal Energies=-2817.362003

Sum of electronic and thermal Enthalpies=-2817.361059

Sum of electronic and thermal Free Energies= -2817.504620

SCF Done: E(wB97XD) = -2819.340233

TS2a-1

Number of Negative Frequencies = 1

Co	0.42128	0.15445	0.14352
P	-0.405	-1.97069	-0.34601
P	1.73603	1.81768	-0.99783
N	2.2043	-0.7544	0.2769
C	0.9965	-2.8301	0.56945
H	0.73192	-2.74022	1.62937
H	1.10556	-3.89575	0.34627
C	2.29117	-2.09945	0.39392
C	3.51886	-2.76265	0.40628

H	3.53159	-3.84613	0.48703
C	4.71429	-2.04603	0.33286
C	4.60724	-0.65378	0.25232
H	5.49915	-0.0348	0.20726
C	3.3564	-0.0422	0.22344
C	3.19431	1.44368	0.12359
C	-0.20726	0.69848	4.30743
C	0.98111	-0.32918	4.31981
B	0.37113	0.14597	2.11284
O	1.41399	-0.31462	2.94037
O	-0.6735	0.60543	2.94292
C	-0.46741	-2.91973	-2.01189
H	-1.45026	-2.64611	-2.4188
C	-0.38273	-4.45381	-1.9681
H	-0.49252	-4.8655	-2.98086
H	0.58918	-4.79449	-1.59176
H	-1.16087	-4.90657	-1.34763
C	0.5913	-2.35752	-2.96897
H	0.4895	-1.27366	-3.07182
H	1.60703	-2.56796	-2.61127
H	0.49242	-2.80957	-3.96483
C	-1.92875	-2.66213	0.57813
H	-2.13801	-1.85554	1.28931
C	-1.74294	-3.95108	1.40015
H	-0.99356	-3.84282	2.18973
H	-2.69148	-4.20699	1.8913
H	-1.45691	-4.80805	0.78149
C	-3.15129	-2.80881	-0.34544
H	-3.06192	-3.68745	-0.9939
H	-4.05758	-2.94481	0.25845
H	-3.31511	-1.93811	-0.9857
C	0.53587	-1.76391	4.64641
H	1.36357	-2.4486	4.43244
H	0.25989	-1.88041	5.70072
H	-0.32	-2.05993	4.03133
C	-1.36607	0.37008	5.25051
H	-1.03649	0.36733	6.29677
H	-2.15049	1.12759	5.14711
H	-1.80859	-0.60278	5.02283
C	2.16598	0.05243	5.20922
H	1.8665	0.1174	6.26246
H	2.94821	-0.71012	5.12736
H	2.59979	1.00992	4.91107
C	0.24571	2.15041	4.53557
H	1.07366	2.41247	3.86944
H	-0.59087	2.82078	4.31302
H	0.56037	2.32646	5.57071
H	4.13266	1.92985	-0.16375
H	2.89903	1.81884	1.11131
C	2.58428	1.91674	-2.7162
H	1.75679	2.18643	-3.38835
C	3.71365	2.94263	-2.9109
H	4.54163	2.77131	-2.21263
H	4.12713	2.85352	-3.92502
H	3.37984	3.9754	-2.7867
C	3.08492	0.5246	-3.13546
H	3.97691	0.23523	-2.56757
H	2.32803	-0.24605	-2.97969
H	3.35948	0.5244	-4.19838
C	1.35422	3.63406	-0.5837
H	0.62397	3.52378	0.22567
C	0.6474	4.33824	-1.75597
H	0.22434	5.29418	-1.42225
H	1.33712	4.55781	-2.57735
H	-0.17334	3.73835	-2.16353
C	2.4988	4.50163	-0.03081
H	3.30232	4.65951	-0.75628
H	2.10644	5.49045	0.24257
H	2.94181	4.07032	0.87208
C	6.0555	-2.73519	0.31975
H	6.44531	-2.81782	-0.70361
H	6.79608	-2.17734	0.90347
H	5.98946	-3.74822	0.7291
Si	-1.95866	1.42665	-0.08554
C	-2.22963	1.20446	-1.98562
H	-2.18048	0.13417	-2.20471
H	-1.37481	1.66354	-2.49477
C	-2.20019	3.27687	0.31182
H	-1.53865	3.95258	-0.23634
H	-3.23196	3.5685	0.08096
H	-2.03826	3.44397	1.38395
C	-3.32858	0.59408	0.9226
H	-4.2009	1.25971	0.94393
H	-3.65879	-0.3637	0.52041
H	-2.97921	0.45296	1.9495
C	-3.51744	1.79574	-2.49742
C	-4.71082	1.06585	-2.48436
C	-3.60569	3.10426	-3.00028

C	-5.92612	1.56638	-2.93162
C	-4.8114	3.6406	-3.45521
C	-5.97683	2.87234	-3.4216
H	-6.80581	0.93188	-2.89209
H	-4.83846	4.65654	-3.83941
H	-6.91904	3.28095	-3.77541
H	-0.51847	1.33951	0.58966
F	-4.68458	-0.21145	-2.01594
H	-2.70163	3.70736	-3.0376

Thermal correction to Energy= 0.970850

Thermal correction to Enthalpy=0.971794

Thermal correction to Gibbs Free Energy= 0.829154

Sum of electronic and zero-point Energies=-2817.416501

Sum of electronic and thermal Energies=-2817.362006

Sum of electronic and thermal Enthalpies=-2817.361062

Sum of electronic and thermal Free Energies=-2817.503701

SCF Done: E(wB97XD) = -2819.340505

Int3a

Number of Negative Frequencies = 0

Co	0.3681	0.10215	-0.11444
P	-0.37952	-1.97639	-0.22157
P	1.72523	1.72704	-1.07421
N	2.22068	-0.77028	0.18028
C	1.23986	-2.92657	-0.32804
H	1.21616	-3.92674	0.11216
H	1.42618	-3.06874	-1.40209
C	2.38494	-2.11338	0.20221
C	3.59278	-2.70266	0.57476
H	3.67048	-3.78646	0.59014
C	4.70311	-1.91005	0.88152
C	4.5453	-0.52837	0.749
H	5.38354	0.14022	0.92163
C	3.30719	0.00891	0.39854
C	3.10311	1.4797	0.19064
C	-0.46273	0.27432	4.30471
C	1.10221	0.06269	4.2465
B	0.27655	0.50535	2.13684
O	1.42739	0.53202	2.91136
O	-0.84272	0.21101	2.90087
C	-1.27135	-2.73907	-1.71785
H	-2.31019	-2.4034	-1.61185
C	-1.25752	-4.27702	-1.79021
H	-1.8817	-4.61731	-2.6268
H	-0.24407	-4.65701	-1.97263
H	-1.63186	-4.75677	-0.88374
C	-0.71897	-2.15936	-3.02963
H	-0.6487	-1.07054	-2.99432
H	0.28218	-2.55065	-3.24912
H	-1.36513	-2.44328	-3.87177
C	-1.26968	-2.7596	1.30073
H	-1.2191	-1.93337	2.01669
C	-0.55655	-3.96802	1.93274
H	0.48423	-3.75841	2.19941
H	-1.07723	-4.25459	2.85551
H	-0.56592	-4.84609	1.27404
C	-2.76277	-3.08687	1.11685
H	-2.9285	-3.95336	0.46808
H	-3.19588	-3.33062	2.09554
H	-3.32863	-2.24557	0.71562
C	1.52804	-1.40904	4.33091
H	2.59205	-1.48481	4.08726
H	1.37275	-1.81777	5.33425
H	0.97531	-2.02314	3.61606
C	-1.23613	-0.7963	5.0724
H	-0.94806	-0.80288	6.13019
H	-2.30829	-0.58486	5.0162
H	-1.06726	-1.79518	4.6618
C	1.90339	0.88984	5.25268
H	1.64748	0.61112	6.28207
H	2.97367	0.70358	5.11003
H	1.72982	1.95981	5.1279
C	-0.87035	1.66819	4.80386
H	-0.34206	2.45651	4.25992
H	-1.94319	1.80514	4.63251
H	-0.67399	1.78963	5.87508
H	4.04775	1.97707	-0.05563
H	2.72589	1.92401	1.1196
C	2.57497	1.47009	-2.75661
H	1.82169	1.85101	-3.46203
C	3.88839	2.22979	-3.00291
H	4.67025	1.92064	-2.2998
H	4.26026	2.01353	-4.01231
H	3.77157	3.3137	-2.92489
C	2.76122	-0.02544	-3.06019
H	3.56623	-0.45778	-2.45417
H	1.84657	-0.58857	-2.85814
H	3.03291	-0.16303	-4.1143

C	1.46499	3.60578	-0.95163
H	0.68845	3.67162	-0.18325
C	0.88157	4.19065	-2.24833
H	0.5582	5.22475	-2.07564
H	1.62377	4.21321	-3.05445
H	0.00666	3.6407	-2.60093
C	2.65251	4.45447	-0.45818
H	3.50593	4.43037	-1.14297
H	2.33237	5.50171	-0.37525
H	3.00371	4.14393	0.52989
C	6.0104	-2.51935	1.31834
H	6.85921	-1.87929	1.0536
H	6.0321	-2.65601	2.40766
H	6.16813	-3.50459	0.86518
Si	-1.72228	1.12774	-0.62516
C	-2.15129	1.31785	-2.5405
H	-2.22585	0.30293	-2.94563
H	-1.29826	1.7909	-3.04095
C	-1.95218	2.90485	0.09596
H	-1.37932	3.6762	-0.42793
H	-3.00776	3.19676	0.03161
H	-1.6694	2.9291	1.15568
C	-3.31986	0.28911	0.04455
H	-4.18445	0.95389	-0.07424
H	-3.5581	-0.64016	-0.48617
H	-3.21705	0.05718	1.11186
C	-3.42008	2.05314	-2.86155
C	-4.66056	1.40291	-2.98153
C	-3.45115	3.44076	-3.04609
C	-5.84081	2.09336	-3.25731
H	-4.68767	0.32279	-2.85643
C	-4.60438	4.16121	-3.32407
C	-5.81815	3.47918	-3.42856
H	-6.77711	1.54927	-3.34056
H	-4.53629	5.23691	-3.45677
H	-6.73204	4.02676	-3.6456
F	-2.28158	4.13409	-2.9603
H	0.2509	1.23309	1.12478

Thermal correction to Energy= 0.972796

Thermal correction to Enthalpy= 0.973740

Thermal correction to Gibbs Free Energy= 0.830451

Sum of electronic and zero-point Energies= -2817.439404

Sum of electronic and thermal Energies= -2817.384694

Sum of electronic and thermal Enthalpies= -2817.383749

Sum of electronic and thermal Free Energies= -2817.527038

SCF Done: E(wB97XD) = -2819.3711

TS4a

Number of Negative Frequencies = 1

Co	0.26409	0.02901	-0.2485
P	-0.56034	-2.03494	-0.14298
P	1.59974	1.74823	-0.88243
N	2.06781	-0.86676	0.15316
C	1.02869	-3.02184	-0.28433
H	1.00237	-4.00999	0.18237
H	1.16651	-3.19092	-1.36111
C	2.20329	-2.22095	0.19014
C	3.39917	-2.84472	0.5415
H	3.4388	-3.93059	0.56328
C	4.54403	-2.09096	0.81556
C	4.41702	-0.7054	0.69416
H	5.27553	-0.05907	0.85612
C	3.18948	-0.12642	0.37218
C	3.04378	1.35886	0.24717
C	-0.04101	0.11219	4.60657
C	1.47537	0.36916	4.25799
B	0.15905	0.7458	2.41473
O	1.38173	1.0512	2.97239
O	-0.66008	0.06666	3.28813
C	-1.54664	-2.87692	-1.52792
H	-2.57107	-2.51698	-1.38974
C	-1.56973	-4.41528	-1.49258
H	-2.2249	-4.79335	-2.28849
H	-0.57408	-4.83811	-1.6715
H	-1.93967	-4.81774	-0.54607
C	-1.05961	-2.3907	-2.90204
H	-0.98192	-1.30252	-2.93549
H	-0.0721	-2.80159	-3.14745
H	-1.75031	-2.72108	-3.68856
C	-1.37479	-2.67167	1.4741
H	-1.19568	-1.83778	2.15783
C	-0.73694	-3.92056	2.10396
H	0.32687	-3.77821	2.32573
H	-1.23889	-4.14542	3.05454
H	-0.83198	-4.81042	1.4707
C	-2.90238	-2.83496	1.38107
H	-3.19762	-3.69661	0.77297
H	-3.30878	-2.99574	2.38814

H	-3.38916	-1.94824	0.97026
C	2.27615	-0.91898	4.0297
H	3.25269	-0.66334	3.6078
H	2.43652	-1.46349	4.96654
H	1.77158	-1.58246	3.3215
C	-0.32942	-1.20161	5.33089
H	0.16419	-1.22244	6.30942
H	-1.40736	-1.30051	5.49493
H	0.00526	-2.06686	4.75431
C	2.22053	1.28234	5.23056
H	2.26201	0.83638	6.23107
H	3.24913	1.42716	4.88397
H	1.74881	2.26449	5.30593
C	-0.70141	1.27847	5.35554
H	-0.51637	2.23179	4.85072
H	-1.78326	1.116	5.37982
H	-0.3399	1.35348	6.38673
H	3.99282	1.82459	-0.03787
H	2.756	1.76061	1.22587
C	2.3117	1.66797	-2.63805
H	1.51856	2.12258	-3.24677
C	3.61617	2.44077	-2.88833
H	4.44018	2.04881	-2.27996
H	3.91446	2.33579	-3.93972
H	3.52228	3.5099	-2.68076
C	2.45609	0.20608	-3.09263
H	3.28086	-0.29331	-2.56999
H	1.54106	-0.35908	-2.89304
H	2.67335	0.16632	-4.16796
C	1.36049	3.60405	-0.52836
H	0.60911	3.58478	0.27062
C	0.75285	4.36312	-1.71906
H	0.48031	5.37855	-1.40506
H	1.46829	4.46206	-2.5433
H	-0.15315	3.89595	-2.1062
C	2.5803	4.37004	0.01817
H	3.40515	4.41544	-0.70047
H	2.28346	5.40436	0.23567
H	2.96571	3.94316	0.94839
C	5.84454	-2.73878	1.21758
H	6.70437	-2.12928	0.92059
H	5.89747	-2.86906	2.30695
H	5.95483	-3.7307	0.76675
Si	-1.77784	1.09414	-0.94721
C	-1.85847	1.51154	-2.88033
H	-1.70748	0.56515	-3.41248
H	-0.99793	2.14274	-3.12809
C	-2.20424	2.75943	-0.06112
H	-1.4679	3.55735	-0.1916
H	-3.16789	3.14713	-0.41663
H	-2.30577	2.57714	1.01737
C	-3.4819	0.22489	-0.65979
H	-4.29901	0.86523	-1.01548
H	-3.59661	-0.74514	-1.15767
H	-3.63909	0.07074	0.41545
C	-3.12473	2.14454	-3.37775
C	-4.21089	1.38822	-3.85348
C	-3.31386	3.53145	-3.38126
C	-5.39666	1.98068	-4.29046
H	-4.11132	0.30559	-3.8785
C	-4.47711	4.15545	-3.81148
C	-5.53586	3.36981	-4.27029
H	-6.21017	1.35598	-4.64997
H	-4.53484	5.23917	-3.78329
H	-6.45397	3.84102	-4.60981
F	-2.29579	4.32719	-2.95048
H	-0.22557	1.19321	1.35832

Thermal correction to Energy= 0.972373

Thermal correction to Enthalpy= 0.973317

Thermal correction to Gibbs Free Energy= 0.832373

Sum of electronic and zero-point Energies= -2817.437421

Sum of electronic and thermal Energies= -2817.383360

Sum of electronic and thermal Enthalpies= -2817.382415

Sum of electronic and thermal Free Energies= -2817.523360

SCF Done: E(wB97XD) = -2819.3683

Int5a

Number of Negative Frequencies = 0

Co	0.3771	0.09315	-0.15644
P	-0.37952	-1.98239	-0.22757
P	1.72223	1.72704	-1.06521
N	2.21768	-0.77328	0.16828
C	1.23986	-2.93257	-0.34004
H	1.21916	-3.93274	0.09716
H	1.42318	-3.06874	-1.41409
C	2.38194	-2.11938	0.19321
C	3.58678	-2.70866	0.57176
H	3.66448	-3.79246	0.58714

C	4.69711	-1.91605	0.88452
C	4.5393	-0.53437	0.755
H	5.37754	0.13422	0.93363
C	3.30419	0.00591	0.39554
C	3.10311	1.4767	0.19364
C	-0.45373	0.26832	4.34671
C	1.11121	0.07469	4.2795
B	0.27355	0.55035	2.19084
O	1.42739	0.57702	2.95336
O	-0.83672	0.22301	2.94287
C	-1.28035	-2.75107	-1.71485
H	-2.31919	-2.4154	-1.60285
C	-1.26652	-4.28902	-1.78121
H	-1.8937	-4.63231	-2.6148
H	-0.25607	-4.66901	-1.96663
H	-1.64086	-4.76577	-0.87174
C	-0.73697	-2.17436	-3.03263
H	-0.6667	-1.08554	-2.99732
H	0.26418	-2.56565	-3.25512
H	-1.38613	-2.46128	-3.86877
C	-1.25768	-2.7596	1.30673
H	-1.1981	-1.93337	2.0197
C	-0.54755	-3.97102	1.93274
H	0.49623	-3.76441	2.19341
H	-1.06224	-4.25459	2.86151
H	-0.56292	-4.84909	1.27704
C	-2.75377	-3.07787	1.13185
H	-2.9285	-3.94136	0.48308
H	-3.18088	-3.32462	2.11354
H	-3.31963	-2.23357	0.73962
C	1.55204	-1.39404	4.32791
H	2.61605	-1.45481	4.07526
H	1.40875	-1.82677	5.32525
H	0.99931	-1.99914	3.60406
C	-1.21513	-0.8233	5.0994
H	-0.92706	-0.84188	6.15719
H	-2.29029	-0.62086	5.0462
H	-1.03726	-1.81318	4.6738
C	1.90939	0.88684	5.30068
H	1.65648	0.58712	6.32407
H	2.97967	0.71258	5.15203
H	1.72382	1.95981	5.1969
C	-0.87635	1.65019	4.86686
H	-0.35406	2.45051	4.33192
H	-1.94919	1.77814	4.69851
H	-0.67699	1.75963	5.93808
H	4.04775	1.97107	-0.05263
H	2.72889	1.92101	1.1256
C	2.56297	1.47309	-2.75361
H	1.80969	1.85701	-3.45303
C	3.87639	2.23279	-2.99991
H	4.66125	1.92064	-2.2998
H	4.24526	2.01653	-4.01231
H	3.76257	3.3167	-2.91889
C	2.74922	-0.02244	-3.06019
H	3.55723	-0.45778	-2.46017
H	1.83457	-0.58557	-2.85514
H	3.01491	-0.15703	-4.1173
C	1.46199	3.60578	-0.94263
H	0.68545	3.67162	-0.17125
C	0.87557	4.19365	-2.23633
H	0.5552	5.22775	-2.06064
H	1.61777	4.21621	-3.04245
H	0.00066	3.6437	-2.58893
C	2.64951	4.45147	-0.44918
H	3.50293	4.42737	-1.13397
H	2.33237	5.49872	-0.36325
H	3.00371	4.14093	0.53889
C	6.0044	-2.52535	1.32734
H	6.85321	-1.88529	1.06859
H	6.0201	-2.66501	2.41666
H	6.16213	-3.50759	0.87118
Si	-1.71328	1.12774	-0.66116
C	-2.15129	1.32685	-2.5735
H	-2.22585	0.31193	-2.98163
H	-1.30126	1.8029	-3.07396
C	-1.94918	2.89885	0.07196
H	-1.38232	3.6762	-0.44893
H	-3.00776	3.18776	0.01661
H	-1.6604	2.9201	1.13168
C	-3.31086	0.28611	0.01455
H	-4.17845	0.94789	-0.10724
H	-3.5461	-0.64916	-0.51017
H	-3.20805	0.06018	1.08186
C	-3.42308	2.06214	-2.88255
C	-4.66356	1.40891	-2.99953
C	-3.45715	3.44976	-3.05809
C	-5.84681	2.10236	-3.26331

H	-4.69067	0.32879	-2.88043
C	-4.61338	4.17021	-3.32707
C	-5.82715	3.48818	-3.42856
H	-6.78311	1.55527	-3.34656
H	-4.54529	5.24591	-3.45377
H	-6.74104	4.03576	-3.6366
F	-2.28758	4.14309	-2.9753
H	0.2299	1.20009	1.14878

Thermal correction to Energy= 0.972366

Thermal correction to Enthalpy= 0.973311

Thermal correction to Gibbs Free Energy= 0.825295

Sum of electronic and zero-point Energies= -2817.447890

Sum of electronic and thermal Energies= -2817.392761

Sum of electronic and thermal Enthalpies= -2817.391816

Sum of electronic and thermal Free Energies= -2817.539832

SCF Done: E(wB97XD) = -2819.3718264

TS6a

Number of Negative Frequencies = 1

Co	0.13826	-0.67388	-1.87651
P	-0.3785	-2.24103	-0.39407
P	1.15118	1.33652	-1.3741
N	2.11074	-1.33223	-1.45036
C	1.0918	-3.3851	-0.68647
H	1.25726	-4.13408	0.09459
H	0.84651	-3.92457	-1.60961
C	2.32266	-2.55687	-0.9171
C	3.60205	-2.99921	-0.57758
H	3.72749	-3.99066	-0.15135
C	4.71042	-2.17069	-0.77678
C	4.46594	-0.8917	-1.28725
H	5.28399	-0.19085	-1.42886
C	3.16666	-0.4982	-1.60766
C	2.82941	0.88218	-2.09107
C	-1.81173	-3.46185	-0.61189
H	-2.70346	-2.83464	-0.50885
C	-1.9142	-4.60741	0.41014
H	-2.74994	-5.26487	0.13737
H	-1.00927	-5.22675	0.42623
H	-2.10016	-4.25233	1.42661
C	-1.82099	-4.04291	-2.03817
H	-1.63432	-3.27898	-2.79588
H	-1.06542	-4.82997	-2.15323
H	-2.79584	-4.50137	-2.24344
C	-0.30767	-1.84161	1.46657
H	0.37671	-0.98453	1.46791
C	0.28414	-2.88222	2.43102
H	1.30805	-3.1564	2.15495
H	0.32586	-2.46036	3.44431
H	-0.30745	-3.80002	2.48526
C	-1.67156	-1.31501	1.94556
H	-2.42398	-2.10981	1.99792
H	-1.5806	-0.88394	2.95052
H	-2.05313	-0.53263	1.28014
H	2.67642	0.85363	-3.17636
H	3.63702	1.59008	-1.87741
C	0.83109	3.03964	-2.15671
H	-0.24754	3.18029	-2.02215
C	1.54602	4.25352	-1.54088
H	2.63623	4.15743	-1.60349
H	1.26831	5.15677	-2.10003
H	1.2764	4.42291	-0.49559
C	1.10773	3.0113	-3.67283
H	2.18502	3.03663	-3.87558
H	0.69092	2.13343	-4.16908
H	0.67129	3.90362	-4.13858
C	1.54572	1.60409	0.46716
H	1.64402	0.55991	0.79259
C	0.32921	2.18598	1.20723
H	0.48891	2.13956	2.29218
H	0.15557	3.23694	0.95033
H	-0.58478	1.63065	0.97557
C	2.85134	2.31127	0.86861
H	2.85389	3.3743	0.61758
H	2.98544	2.23315	1.95611
H	3.7288	1.85054	0.40326
C	6.10919	-2.63494	-0.45788
H	6.10816	-3.41439	0.31068
H	6.59029	-3.05503	-1.35099
H	6.73657	-1.80769	-0.10998
Si	-1.98204	0.14169	-2.3122
C	-2.68583	1.61753	-1.29162
H	-1.98618	2.44893	-1.15427
H	-3.58519	2.01955	-1.7793
H	-2.98811	1.28266	-0.28995
C	-3.55109	-0.97455	-2.37722
H	-3.42482	-1.86844	-2.99733
H	-3.89398	-1.29675	-1.38561
H	-4.36941	-0.38804	-2.81877

C	-1.78959	0.74103	-4.14091
H	-1.63095	1.83137	-4.14412
C	-0.3894	0.19327	-6.17853
C	0.70253	-0.35413	-6.84275
C	1.63031	-1.07822	-6.09185
C	1.44527	-1.23609	-4.71612
C	0.3439	-0.66092	-4.05689
C	-0.61144	0.06591	-4.80484
H	0.80324	-0.21517	-7.91444
H	2.49044	-1.52651	-6.58482
H	2.16145	-1.82616	-4.14868
H	-2.70722	0.58711	-4.72548
H	-0.19086	-1.58329	-2.97353
F	-1.28188	0.91107	-6.91232

Thermal correction to Energy= 0.763976

Thermal correction to Enthalpy= 0.764920

Thermal correction to Gibbs Free Energy= 0.649306

Sum of electronic and zero-point Energies= -2405.747212

Sum of electronic and thermal Energies= -2405.704304

Sum of electronic and thermal Enthalpies= -2405.703359

Sum of electronic and thermal Free Energies= -2405.818974

SCF Done: E(wB97XD) = -2407.43193117

Int7a

Number of Negative Frequencies = 0

Co	0.45076	-1.07586	-1.98062
P	0.03944	-2.59658	-0.30968
P	1.42686	1.10658	-1.42134
N	2.39528	-1.6455	-1.57127
C	1.4276	-3.73865	-0.85422
H	1.64026	-4.5763	-0.18201
H	1.10132	-4.14759	-1.81792
C	2.64384	-2.88134	-1.06909
C	3.92853	-3.30305	-0.73744
H	4.07687	-4.30735	-0.35083
C	5.01738	-2.43598	-0.89715
C	4.73865	-1.15091	-1.36263
H	5.53626	-0.42154	-1.4737
C	3.43072	-0.77898	-1.6887
C	3.09056	0.60667	-2.14945
C	-1.49641	-3.70418	-0.42256
H	-2.32553	-2.99623	-0.29333
C	-1.64914	-4.81246	0.63182
H	-2.56094	-5.38867	0.42808
H	-0.80902	-5.51678	0.60361
H	-1.73079	-4.42366	1.64915
C	-1.62363	-4.32349	-1.82856
H	-1.41931	-3.60493	-2.62513
H	-0.93251	-5.16659	-1.94854
H	-2.63831	-4.71526	-1.9691
C	0.39976	-2.32236	1.5418
H	1.18676	-1.55975	1.47899
C	0.97283	-3.47475	2.38374
H	1.8976	-3.87295	1.95342
H	1.21816	-3.10822	3.38919
H	0.27159	-4.30523	2.50244
C	-0.80819	-1.65655	2.22327
H	-1.63426	-2.36081	2.37386
H	-0.52621	-1.27032	3.20909
H	-1.18772	-0.81595	1.63173
H	2.92554	0.58686	-3.23236
H	3.91093	1.3002	-1.93875
C	1.22249	2.85591	-2.17487
H	0.14545	3.0452	-2.07374
C	1.96638	4.02992	-1.51622
H	3.05417	3.89047	-1.55518
H	1.73958	4.95512	-2.06174
H	1.68028	4.18753	-0.47371
C	1.55543	2.84737	-3.6815
H	2.64053	2.85764	-3.83673
H	1.15309	1.98124	-4.21098
H	1.15226	3.75233	-4.15338
C	1.84826	1.33791	0.4258
H	1.88448	0.28685	0.74224
C	0.66512	1.97853	1.17165
H	0.81434	1.90627	2.25672
H	0.55495	3.04181	0.93084
H	-0.28023	1.48129	0.92918
C	3.18967	1.96363	0.83847
H	3.25254	3.02813	0.60031
H	3.31989	1.86504	1.92493
H	4.04011	1.46011	0.36619
C	6.42511	-2.87682	-0.5815
H	6.83652	-3.48096	-1.40126
H	7.08999	-2.01906	-0.4374
H	6.45869	-3.49325	0.32308
Si	-1.92384	-0.26968	-2.69521
C	-2.56004	0.89018	-1.33326

H	-1.83562	1.67031	-1.08099
H	-3.49057	1.37517	-1.65863
H	-2.78578	0.33468	-0.41455
C	-3.41978	-1.33921	-3.19973
H	-3.15926	-2.0231	-4.01672
H	-3.8071	-1.94202	-2.36935
H	-4.23877	-0.69888	-3.55242
C	-1.26184	0.59617	-4.2369
H	-0.9385	1.6101	-3.96241
C	0.09582	-0.23984	-6.18015
C	1.11187	-0.95595	-6.7989
C	1.97036	-1.68403	-5.9768
C	1.79464	-1.67436	-4.58935
C	0.76371	-0.95328	-3.94654
C	-0.10701	-0.21461	-4.79896
H	1.20903	-0.93433	-7.87965
H	2.77519	-2.2673	-6.42196
H	2.48382	-2.27117	-3.9944
H	-2.05195	0.72327	-4.9864
H	-1.00575	-1.52016	-2.38105
F	-0.74527	0.48026	-6.9806

Thermal correction to Energy= 0.765635

Thermal correction to Enthalpy= 0.766579

Thermal correction to Gibbs Free Energy= 0.649624

Sum of electronic and zero-point Energies= -2405.753619

Sum of electronic and thermal Energies= -2405.710279

Sum of electronic and thermal Enthalpies= -2405.709335

Sum of electronic and thermal Free Energies= -2405.826289

SCF Done: E(wB97XD) = -2407.44223299

TS8a

Number of Negative Frequencies = 1

Co	0.48328	-1.05816	-1.99821
P	0.05423	-2.58502	-0.31462
P	1.44956	1.08182	-1.42923
N	2.41936	-1.66451	-1.58824
C	1.4206	-3.74381	-0.88352
H	1.6173	-4.59765	-0.22639
H	1.08387	-4.12612	-1.85409
C	2.64892	-2.90264	-1.0822
C	3.92444	-3.33005	-0.72385
H	4.05945	-4.33521	-0.33394
C	5.02055	-2.46804	-0.85904
C	4.75727	-1.17912	-1.32567
H	5.56084	-0.45332	-1.4147
C	3.45922	-0.801	-1.67965
C	3.12731	0.5898	-2.13474
C	-1.49486	-3.67948	-0.39315
H	-2.31685	-2.9588	-0.27969
C	-1.65456	-4.75722	0.69237
H	-2.57934	-5.32112	0.51296
H	-0.82796	-5.47734	0.67066
H	-1.71738	-4.34139	1.70045
C	-1.63717	-4.33884	-1.77963
H	-1.43317	-3.645	-2.59827
H	-0.95377	-5.19085	-1.87846
H	-2.65619	-4.72641	-1.90068
C	0.45505	-2.31122	1.52866
H	1.25609	-1.56433	1.4456
C	1.02564	-3.46996	2.36311
H	1.93061	-3.8898	1.91176
H	1.30485	-3.10104	3.35956
H	0.31143	-4.28476	2.5076
C	-0.724	-1.61847	2.23349
H	-1.56188	-2.30426	2.40218
H	-0.41197	-1.23955	3.21479
H	-1.09713	-0.76839	1.65152
H	2.97785	0.58025	-3.22032
H	3.94478	1.282	-1.90667
C	1.23829	2.82759	-2.18679
H	0.1603	3.0132	-2.08174
C	1.9813	4.00342	-1.53066
H	3.06798	3.86449	-1.56736
H	1.75514	4.92771	-2.07973
H	1.69307	4.16559	-0.48934
C	1.56826	2.81877	-3.69345
H	2.65331	2.82956	-3.85092
H	1.16466	1.9531	-4.22166
H	1.16527	3.72453	-4.16414
C	1.84035	1.32414	0.42114
H	1.87453	0.27587	0.7456
C	0.64422	1.96845	1.14288
H	0.76889	1.8951	2.23094
H	0.54167	3.03199	0.90029
H	-0.29638	1.4734	0.87848
C	3.17511	1.95488	0.8524
H	3.24225	3.01727	0.60773
H	3.28706	1.86406	1.94186

H	4.03254	1.44754	0.3983
C	6.41808	-2.91615	-0.51338
H	6.84823	-3.51166	-1.32949
H	7.08244	-2.06329	-0.34306
H	6.42734	-3.54414	0.38429
Si	-2.01902	-0.27647	-2.74213
C	-2.65767	0.84495	-1.35112
H	-1.92175	1.60561	-1.07383
H	-3.58256	1.35121	-1.66112
H	-2.8883	0.26096	-0.45203
C	-3.48393	-1.36062	-3.2891
H	-3.19559	-2.02427	-4.11244
H	-3.86817	-1.98542	-2.47338
H	-4.31219	-0.73183	-3.64182
C	-1.29556	0.62343	-4.23489
H	-0.97032	1.62252	-3.91062
C	0.06651	-0.17992	-6.18489
C	1.09668	-0.86417	-6.81679
C	1.98122	-1.57519	-6.00741
C	1.81494	-1.58044	-4.61838
C	0.77167	-0.89041	-3.96059
C	-0.1265	-0.16809	-4.80044
H	1.18333	-0.83217	-7.89822
H	2.79767	-2.13341	-6.46285
H	2.52139	-2.16373	-4.03235
H	-2.0579	0.78886	-5.00495
H	-1.00086	-1.60363	-2.43885
F	-0.79781	0.52181	-6.97663

Thermal correction to Energy= 0.764972

Thermal correction to Enthalpy= 0.765916

Thermal correction to Gibbs Free Energy= 0.647638

Sum of electronic and zero-point Energies= -2405.736611

Sum of electronic and thermal Energies= -2405.693217

Sum of electronic and thermal Enthalpies= -2405.692273

Sum of electronic and thermal Free Energies= -2405.810551

SCF Done: E(wB97XD) = -2407.41796192

Int9a

Number of Negative Frequencies = 0

Co	0.48076	-1.09086	-1.96262
P	0.05444	-2.60858	-0.30968
P	1.42386	1.11558	-1.41534
N	2.39528	-1.6485	-1.57127
C	1.4426	-3.75065	-0.84822
H	1.66426	-4.5823	-0.17001
H	1.11332	-4.16859	-1.80592
C	2.64984	-2.88734	-1.07209
C	3.93753	-3.30005	-0.74344
H	4.08887	-4.30435	-0.35683
C	5.02338	-2.42998	-0.90315
C	4.73565	-1.14491	-1.36563
H	5.53026	-0.41254	-1.4767
C	3.42772	-0.77898	-1.6917
C	3.08156	0.60667	-2.14945
C	-1.48741	-3.70418	-0.42556
H	-2.31353	-2.99023	-0.28733
C	-1.64614	-4.82146	0.61982
H	-2.56094	-5.39167	0.41008
H	-0.80902	-5.52878	0.58561
H	-1.72779	-4.43866	1.64015
C	-1.62063	-4.30249	-1.84056
H	-1.40731	-3.57193	-2.62513
H	-0.93551	-5.14859	-1.97254
H	-2.63831	-4.68526	-1.9871
C	0.40276	-2.33136	1.5448
H	1.19276	-1.56875	1.48499
C	0.96983	-3.48375	2.38974
H	1.8976	-3.88195	1.96542
H	1.20916	-3.11722	3.39819
H	0.26859	-4.31423	2.50544
C	-0.80819	-1.66255	2.21727
H	-1.63426	-2.36681	2.36786
H	-0.52921	-1.27332	3.20609
H	-1.18472	-0.82195	1.62273
H	2.91354	0.58686	-3.23236
H	3.90493	1.3002	-1.94475
C	1.23149	2.86791	-2.17187
H	0.15445	3.0632	-2.06774
C	1.98138	4.03892	-1.51622
H	3.06617	3.89647	-1.55518
H	1.75758	4.96712	-2.06174
H	1.69528	4.19953	-0.47371
C	1.56143	2.85337	-3.6785
H	2.64653	2.86064	-3.83673
H	1.15609	1.98724	-4.20498
H	1.16126	3.75833	-4.15338
C	1.85126	1.34391	0.4288
H	1.88748	0.29285	0.74524

C	0.67112	1.98753	1.17765
H	0.82034	1.91527	2.26272
H	0.56095	3.05081	0.93684
H	-0.27423	1.49029	0.93518
C	3.19567	1.96963	0.84147
H	3.25854	3.03413	0.60631
H	3.32889	1.86804	1.92793
H	4.04311	1.46611	0.36619
C	6.43111	-2.86482	-0.5875
H	6.84252	-3.47196	-1.40426
H	7.09599	-2.00706	-0.4494
H	6.46769	-3.47825	0.32008
Si	-1.98984	-0.24268	-2.71921
C	-2.59904	0.92018	-1.35426
H	-1.84762	1.67031	-1.08999
H	-3.51157	1.44117	-1.67663
H	-2.84278	0.35868	-0.44455
C	-3.46178	-1.32721	-3.23873
H	-3.17426	-2.0201	-4.03772
H	-3.8581	-1.92102	-2.40535
H	-4.28077	-0.70188	-3.61842
C	-1.25884	0.59617	-4.2369
H	-0.9205	1.6041	-3.95641
C	0.09282	-0.24884	-6.17415
C	1.10887	-0.96195	-6.7959
C	1.96736	-1.69603	-5.9768
C	1.78864	-1.69536	-4.58935
C	0.75771	-0.97728	-3.93754
C	-0.11001	-0.23261	-4.78996
H	1.20603	-0.93433	-7.87665
H	2.77219	-2.2763	-6.42496
H	2.47782	-2.29217	-3.9974
H	-2.03095	0.74427	-5.0014
H	-1.07475	-1.39416	-2.25205
F	-0.74527	0.47726	-6.9716

Thermal correction to Energy= 0.765802
Thermal correction to Enthalpy= 0.766746
Thermal correction to Gibbs Free Energy= 0.645130
Sum of electronic and zero-point Energies= -2405.739152
Sum of electronic and thermal Energies= -2405.694850
Sum of electronic and thermal Enthalpies= -2405.693906
Sum of electronic and thermal Free Energies= -2405.815522
SCF Done: E(wB97XD) = -2407.42108979

TS10a

Number of Negative Frequencies = 1

Co	0.84044	-1.42839	-2.54111
P	0.50582	-3.77333	-2.49722
P	1.65941	0.67809	-1.89407
N	2.54285	-2.1135	-1.13626
C	2.22155	-4.36672	-2.0279
H	2.22288	-5.37071	-1.59311
H	2.76506	-4.41461	-2.97705
C	2.95431	-3.39679	-1.13061
C	4.04884	-3.80965	-0.36536
H	4.34276	-4.85643	-0.36968
C	4.7709	-2.87768	0.38814
C	4.3335	-1.55013	0.35392
H	4.85043	-0.78932	0.93306
C	3.20881	-1.20226	-0.39963
C	2.6392	0.19614	-0.35345
C	0.05165	-4.98353	-3.8918
H	-0.9492	-4.64373	-4.18653
C	-0.03122	-6.46834	-3.48825
H	-0.28463	-7.06691	-4.37308
H	0.93205	-6.83792	-3.11588
H	-0.78868	-6.6734	-2.73028
C	0.96149	-4.86567	-5.12698
H	1.02351	-3.84579	-5.4951
H	1.97251	-5.23195	-4.91559
H	0.54841	-5.49888	-5.92345
C	-0.58414	-4.30562	-1.03986
H	-0.52124	-3.42674	-0.39598
C	-0.12008	-5.50801	-0.20075
H	0.88157	-5.35829	0.21583
H	-0.80519	-5.63002	0.64753
H	-0.11583	-6.45008	-0.75655
C	-2.0672	-4.42378	-1.43905
H	-2.26959	-5.31421	-2.04376
H	-2.67828	-4.49709	-0.53134
H	-2.40628	-3.54376	-1.99247
H	3.42173	0.91752	-0.10604
H	1.91728	0.23586	0.46932
C	2.96617	1.59101	-2.94569
H	3.34595	0.79551	-3.59049
C	2.35084	2.63497	-3.89325
H	2.03385	3.53596	-3.35718
H	3.10769	2.93995	-4.62717

H	1.50126	2.21972	-4.43652
C	4.1684	2.18427	-2.1903
H	3.87559	2.93939	-1.45301
H	4.76189	1.41978	-1.67762
H	4.83353	2.67742	-2.91142
C	0.50215	1.97333	-1.15109
H	-0.03591	1.36797	-0.41278
C	-0.5592	2.47711	-2.14383
H	-1.34534	3.00593	-1.58944
H	-0.14129	3.17611	-2.87332
H	-1.01811	1.64903	-2.68558
C	1.17673	3.1399	-0.41131
H	1.70497	3.80911	-1.09837
H	0.41209	3.73889	0.10016
H	1.88998	2.8043	0.34944
C	5.98129	-3.28881	1.18966
H	5.81546	-4.24399	1.69974
H	6.85591	-3.41703	0.53856
H	6.23775	-2.53783	1.94307
C	-0.3167	-1.13397	0.81805
C	-0.83862	-1.19201	-6.43991
C	-0.60157	0.34854	-6.08336
B	0.56411	-1.01013	-4.52088
O	-0.12633	-1.90045	-5.40851
O	0.17552	0.31306	-4.87329
C	0.21858	1.11479	-7.13671
H	-0.29053	1.1491	-8.10689
H	0.35835	2.14671	-6.79692
H	1.20873	0.67213	-7.26374
C	-0.24548	-1.62969	-7.79068
H	-0.36539	-2.7135	-7.8973
H	-0.76212	-1.14968	-8.62964
H	0.81937	-1.39903	-7.86358
C	-1.88663	1.13834	-5.79551
H	-1.61878	2.15602	-5.49368
H	-2.52532	1.20773	-6.68404
H	-2.46339	0.69503	-4.98195
C	-2.3066	-1.64788	-6.40661
H	-2.88747	-1.17486	-7.20684
H	-2.34561	-2.7321	-6.55695
H	-2.79127	-1.42705	-5.45456
C	4.10611	-1.19529	-5.99756
C	4.35889	-2.28044	-4.88775
B	2.23312	-1.35882	-4.62516

O	3.02605	-2.45805	-4.32362
O	2.91448	-0.5255	-5.50817
C	3.76014	-1.79356	-7.36831
H	3.43189	-0.99043	-8.0364
H	4.62897	-2.28126	-7.82327
H	2.94985	-2.52445	-7.29434
C	4.84976	-3.63491	-5.40344
H	5.82108	-3.53234	-5.90061
H	4.97751	-4.32579	-4.5631
H	4.14446	-4.08328	-6.10634
C	5.27779	-1.80976	-3.7505
H	5.24517	-2.54178	-2.93799
H	6.31674	-1.71688	-4.08675
H	4.9607	-0.84677	-3.34249
C	5.21159	-0.15137	-6.15979
H	6.14545	-0.61675	-6.49637
H	4.90982	0.58317	-6.91349
H	5.40644	0.38429	-5.22811
C	-0.94779	-1.10958	-1.71172
C	-2.02259	-0.99986	-2.62571
C	-1.30311	-0.98912	-0.33241
C	-3.34336	-0.73598	-2.2586
H	-1.81095	-1.13507	-3.67645
C	-2.63591	-0.70829	-0.00468
C	-3.66995	-0.57136	-0.91302
H	-4.1197	-0.65911	-3.01813
H	-4.67566	-0.35855	-0.56514
H	-0.09803	-0.14463	1.25479
F	-2.95268	-0.56823	1.323
H	0.62269	-1.50153	0.39906
Si	-0.72108	-2.2327	2.33854
H	-1.73524	-3.27302	1.99451
C	0.88191	-3.11719	2.85353
H	1.65095	-2.3889	3.14302
H	0.71412	-3.77572	3.71477
H	1.29625	-3.72707	2.04292
C	-1.30659	-1.22687	3.83263
H	-0.53846	-0.5041	4.13767
H	-2.22175	-0.67323	3.60915
H	-1.50032	-1.88045	4.69195

Thermal correction to Energy= 1.153138

Thermal correction to Enthalpy= 1.154082

Thermal correction to Gibbs Free Energy= 0.992420

Sum of electronic and zero-point Energies= -3227.886376

Sum of electronic and thermal Energies= -3227.821654

Sum of electronic and thermal Enthalpies= -3227.820710

Sum of electronic and thermal Free Energies= -3227.982371

SCF Done: E(wB97XD) = -3230.04404119

Int11a

Number of Negative Frequencies = 0

Co	0.82679	-1.45179	-2.51462
P	0.49753	-3.78433	-2.50015
P	1.65047	0.67599	-1.91061
N	2.56385	-2.09819	-1.20703
C	2.23161	-4.36011	-2.07958
H	2.27272	-5.37444	-1.67351
H	2.75884	-4.35845	-3.03813
C	2.95194	-3.39692	-1.16678
C	4.0066	-3.82134	-0.35465
H	4.27982	-4.86878	-0.34256
C	4.71542	-2.89445	0.42233
C	4.30391	-1.56162	0.35798
H	4.80762	-0.80293	0.95089
C	3.21518	-1.19741	-0.43939
C	2.6682	0.20982	-0.40019
C	0.01088	-4.98781	-3.88641
H	-1.01451	-4.68537	-4.12554
C	0.0144	-6.4804	-3.49968
H	-0.25767	-7.07867	-4.37912
H	1.00998	-6.81173	-3.18275
H	-0.69262	-6.72539	-2.70782
C	0.85205	-4.80137	-5.15953
H	0.82345	-3.7734	-5.51208
H	1.89597	-5.10339	-5.00491
H	0.44393	-5.44693	-5.95085
C	-0.53773	-4.32838	-1.00962
H	-0.4957	-3.4372	-0.38032
C	-0.00793	-5.50237	-0.17308
H	1.00723	-5.3221	0.20108
H	-0.65373	-5.6397	0.7026
H	0.00329	-6.45077	-0.72011
C	-2.02329	-4.51903	-1.37285
H	-2.19426	-5.42802	-1.95798
H	-2.60931	-4.60595	-0.4493
H	-2.41448	-3.66554	-1.93282
H	3.47044	0.92147	-0.18171
H	1.97739	0.2749	0.44683
C	2.92818	1.5919	-2.99763
H	3.28967	0.80262	-3.65972
C	2.28469	2.64507	-3.92237
H	1.97238	3.53571	-3.37387
H	3.02807	2.96112	-4.66664
H	1.43086	2.22385	-4.45859
C	4.14973	2.18856	-2.27384
H	3.87391	2.92956	-1.519
H	4.7706	1.42354	-1.79314
H	4.78317	2.69747	-3.01427
C	0.50348	1.96638	-1.15174
H	-0.00523	1.36774	-0.38523
C	-0.59656	2.43902	-2.11449
H	-1.35353	2.99615	-1.55297
H	-0.20964	3.09974	-2.89449
H	-1.0847	1.59033	-2.59881
C	1.18542	3.14657	-0.44432
H	1.68166	3.81898	-1.15673
H	0.43117	3.74317	0.08905
H	1.93162	2.82924	0.29071
C	5.85625	-3.32821	1.31056
H	5.47809	-3.7159	2.26432
H	6.44144	-4.13131	0.84597
H	6.52627	-2.49707	1.53335
C	-0.31288	-1.11436	0.77401
C	-0.85727	-1.12534	-6.44123
C	-0.65589	0.40207	-6.0329
B	0.51588	-0.99955	-4.51591
O	-0.283	-1.85737	-5.33924
O	0.23073	0.33937	-4.8989
C	0.03253	1.25648	-7.1098
H	-0.56157	1.30254	-8.0309
H	0.14751	2.27999	-6.7402
H	1.02737	0.87463	-7.35043
C	-0.08414	-1.53572	-7.70441
H	-0.17067	-2.62023	-7.84033
H	-0.47765	-1.05074	-8.60759
H	0.97807	-1.28901	-7.62081
C	-1.94826	1.11688	-5.60492
H	-1.69534	2.12789	-5.26725
H	-2.64984	1.20889	-6.44186
H	-2.44614	0.60682	-4.77943
C	-2.3159	-1.56663	-6.60598
H	-2.79451	-1.04753	-7.44212

H	-2.3447	-2.64016	-6.8228
H	-2.90559	-1.38987	-5.70565
C	4.17374	-1.23942	-5.97842
C	4.40551	-2.34406	-4.88426
B	2.28602	-1.41538	-4.62898
O	3.06733	-2.52297	-4.33683
O	2.97028	-0.58002	-5.50267
C	3.85736	-1.81452	-7.36459
H	3.53261	-1.00201	-8.02146
H	4.73705	-2.28551	-7.81611
H	3.05214	-2.55375	-7.31907
C	4.89639	-3.6934	-5.41157
H	5.87189	-3.59542	-5.89693
H	5.00427	-4.39976	-4.5801
H	4.19356	-4.12929	-6.13024
C	5.31114	-1.89043	-3.72776
H	5.27712	-2.63926	-2.92975
H	6.35378	-1.78528	-4.05265
H	4.97921	-0.93961	-3.30517
C	5.28142	-0.19449	-6.09658
H	6.2232	-0.65124	-6.42129
H	4.99321	0.55253	-6.84978
H	5.4522	0.32942	-5.15581
C	-0.98167	-1.1486	-1.75055
C	-2.07806	-1.09819	-2.64667
C	-1.31644	-0.98879	-0.36992
C	-3.39416	-0.83604	-2.26499
H	-1.88455	-1.28487	-3.69763
C	-2.63961	-0.70299	-0.02648
C	-3.69646	-0.6117	-0.91881
H	-4.18519	-0.8007	-3.00765
H	-4.69645	-0.39026	-0.56272
H	-0.0838	-0.12076	1.18861
F	-2.9331	-0.50948	1.29994
H	0.61652	-1.49343	0.34933
Si	-0.70416	-2.17791	2.32065
H	-1.75677	-3.19864	2.03137
C	0.88285	-3.10087	2.81817
H	1.69705	-2.39381	3.0231
H	0.72655	-3.69181	3.72844
H	1.22447	-3.78515	2.03384
C	-1.22619	-1.12601	3.80824
H	-0.42757	-0.42233	4.08489
H	-2.12338	-0.5426	3.58694
H	-1.42775	-1.75549	4.6824

Thermal correction to Energy= 1.155152

Thermal correction to Enthalpy= 1.156097

Thermal correction to Gibbs Free Energy= 0.995333

Sum of electronic and zero-point Energies= -3227.904533

Sum of electronic and thermal Energies= -3227.839789

Sum of electronic and thermal Enthalpies= -3227.838845

Sum of electronic and thermal Free Energies= -3227.999608

SCF Done: E(wB97XD) = -3230.07330293

TS12a

Number of Negative Frequencies = 1

Co	0.87813	-1.55001	-2.03843
P	0.59016	-3.87348	-1.94541
P	2.03229	0.57021	-1.44109
N	2.6927	-2.34682	-0.73251
C	2.3089	-4.54366	-1.72865
H	2.32534	-5.54161	-1.28082
H	2.73048	-4.61545	-2.73541
C	3.17269	-3.58934	-0.94918
C	4.44482	-3.98509	-0.52557
H	4.77424	-5.00268	-0.72042
C	5.28659	-3.08301	0.12454
C	4.79417	-1.78609	0.30343
H	5.4047	-1.02978	0.78947
C	3.50922	-1.45572	-0.12722
C	2.96355	-0.0738	0.05947
C	-0.11577	-5.02931	-3.27405
H	-1.1919	-5.0442	-3.07176
C	0.40054	-6.47755	-3.20627
H	-0.16154	-7.10272	-3.91201
H	1.45659	-6.53662	-3.49221
H	0.29604	-6.92522	-2.21339
C	0.08643	-4.45457	-4.6846
H	-0.31986	-3.44322	-4.77676
H	1.15013	-4.41232	-4.9348
H	-0.40997	-5.10168	-5.42051
C	-0.28542	-4.39483	-0.30941
H	-0.67179	-3.43682	0.04778
C	0.66027	-4.9385	0.77521
H	1.5051	-4.27339	0.97145
H	0.10115	-5.04479	1.71348
H	1.0544	-5.93022	0.52222
C	-1.48821	-5.34379	-0.45318

H	-1.19103	-6.34858	-0.77256	H	-3.20836	1.90669	-2.56361
H	-1.97556	-5.44582	0.52496	H	-4.06895	1.44849	-4.04517
H	-2.24092	-4.96389	-1.14617	H	-3.53276	0.20086	-2.89815
H	3.755	0.61009	0.37475	C	-3.14071	-0.61322	-5.65029
H	2.21902	-0.09266	0.86364	H	-3.64937	0.16457	-6.23297
C	3.40107	1.11931	-2.63775	H	-2.96621	-1.46792	-6.31286
H	3.54378	0.2192	-3.23562	H	-3.80702	-0.94016	-4.84867
C	2.8895	2.19393	-3.61228	C	3.01072	-1.36122	-5.8328
H	2.77316	3.16914	-3.12895	C	3.82756	-2.47413	-5.07369
H	3.60896	2.31903	-4.43098	B	2.0602	-1.7123	-3.70061
H	1.93181	1.90487	-4.05367	O	2.98351	-2.75594	-3.93378
C	4.7707	1.48753	-2.04329	O	2.14589	-0.83311	-4.79332
H	4.74479	2.38179	-1.41554	C	2.11746	-1.91295	-6.95404
H	5.19508	0.66767	-1.45452	H	1.51122	-1.09966	-7.36055
H	5.47137	1.69015	-2.86478	H	2.71282	-2.33433	-7.77198
C	1.42874	2.18876	-0.58975	H	1.43525	-2.68182	-6.58295
H	0.9679	1.79186	0.32027	C	4.04527	-3.77148	-5.85935
C	0.33029	2.95871	-1.33649	H	4.66796	-3.5974	-6.74511
H	-0.05828	3.75188	-0.68309	H	4.56427	-4.49974	-5.22607
H	0.71337	3.44047	-2.24191	H	3.10351	-4.21833	-6.1848
H	-0.48572	2.30194	-1.63284	C	5.18656	-2.00205	-4.52917
C	2.52582	3.16676	-0.1271	H	5.60152	-2.78593	-3.88701
H	2.99618	3.68434	-0.96804	H	5.90239	-1.80377	-5.33498
H	2.06423	3.9373	0.5046	H	5.08993	-1.09715	-3.92342
H	3.31385	2.69378	0.46654	C	3.85526	-0.21622	-6.39981
C	6.65094	-3.48756	0.62377	H	4.52967	-0.57786	-7.18566
H	6.58907	-3.89388	1.64222	H	3.19737	0.53844	-6.8433
H	7.09562	-4.26258	-0.00915	H	4.45757	0.27421	-5.63196
H	7.33618	-2.63413	0.65462	C	-1.24867	-1.46695	-1.54885
C	-0.92307	-0.02119	0.59708	C	-2.17691	-2.37633	-2.14339
C	-1.79809	-0.11103	-5.10671	C	-1.7551	-0.84314	-0.35595
C	-1.90709	1.08167	-4.07491	C	-3.49065	-2.58299	-1.72604
B	-0.68401	-0.64962	-3.1187	H	-1.88027	-2.88276	-3.05194
O	-1.20514	-1.16598	-4.32138	C	-3.08873	-1.04293	0.01355
O	-0.91416	0.73617	-3.08406	C	-3.98211	-1.88173	-0.62858
C	-1.56555	2.45984	-4.64835	H	-4.13302	-3.27322	-2.26833
H	-2.26823	2.73912	-5.4424	H	-5.00056	-1.976	-0.26626
H	-1.63473	3.21576	-3.85919	H	-1.23322	1.03397	0.57813
H	-0.5522	2.48807	-5.05586	F	-3.54551	-0.3845	1.12423
C	-0.85533	0.1828	-6.28054	H	0.10478	-0.06889	0.24219
H	-0.7504	-0.72597	-6.88116	Si	-0.95752	-0.58032	2.44155
H	-1.24753	0.97485	-6.92875	H	-1.66529	-1.88747	2.56063
H	0.13843	0.46101	-5.92293	C	0.80318	-0.86076	3.09839
C	-3.26634	1.15587	-3.35869	H	1.40056	0.05969	3.0883

H	0.76476	-1.21014	4.13792
H	1.33824	-1.62034	2.51615
C	-1.79413	0.71644	3.53856
H	-1.23504	1.66082	3.52733
H	-2.81248	0.92278	3.19588
H	-1.84683	0.3772	4.58018

Thermal correction to Energy= 1.153766

Thermal correction to Enthalpy= 1.154710

Thermal correction to Gibbs Free Energy= 0.995833

Sum of electronic and zero-point Energies= -3227.865109

Sum of electronic and thermal Energies= -3227.801200

Sum of electronic and thermal Enthalpies= -3227.800256

Sum of electronic and thermal Free Energies= -3227.959133

SCF Done: E(wB97XD) = -3230.02723874

TS2b-1

Number of Negative Frequencies = 1

Co	0.52391	-0.26643	-0.61241
P	0.36198	-2.55586	-0.34382
P	1.39103	1.77934	-1.01288
N	2.56978	-0.78281	-0.82616
C	1.81491	-2.98832	-1.43846
H	2.13815	-4.03076	-1.37476
H	1.45542	-2.80371	-2.46005
C	2.9424	-2.04057	-1.15119
C	4.28269	-2.41953	-1.23115
H	4.53192	-3.44176	-1.50238
C	5.29355	-1.48731	-0.98057
C	4.89031	-0.18691	-0.65796
H	5.62997	0.58351	-0.45726
C	3.53584	0.13563	-0.58316
C	3.05417	1.50883	-0.20948
H	-0.17385	-0.54641	-1.91038
C	0.76042	1.08464	3.54619
C	-0.59516	0.29818	3.50096
B	0.42705	0.18872	1.39514
O	-0.46617	-0.45312	2.26802
O	1.09156	1.19096	2.13735
C	-1.0115	-3.63301	-1.07669
H	-1.86299	-3.46923	-0.40498
C	-0.7035	-5.14128	-1.11258
H	-1.56704	-5.67813	-1.52585
H	0.15259	-5.36373	-1.76048
H	-0.49972	-5.56306	-0.12578
C	-1.43675	-3.16164	-2.47938
H	-1.75267	-2.11821	-2.48419
H	-0.62845	-3.2831	-3.21038
H	-2.27918	-3.77575	-2.82252
C	0.79274	-3.33695	1.33817
H	1.25914	-2.48765	1.84943
C	1.80987	-4.49091	1.33087
H	2.76813	-4.19024	0.895
H	2.00799	-4.80508	2.36431
H	1.4518	-5.37173	0.78806
C	-0.46813	-3.69469	2.14423
H	-1.00219	-4.55278	1.72245
H	-0.18206	-3.96322	3.16942
H	-1.15038	-2.84408	2.20636
C	-1.82489	1.20687	3.3487
H	-2.69524	0.58711	3.11215
H	-2.03751	1.76681	4.26671
H	-1.69471	1.91669	2.52634
C	0.67929	2.48955	4.14469
H	0.36882	2.45169	5.19597
H	1.66391	2.96837	4.10358
H	-0.02496	3.12178	3.59899
C	-0.81876	-0.67713	4.65751
H	-0.85816	-0.14754	5.6173
H	-1.77365	-1.19637	4.52279
H	-0.0304	-1.4319	4.71023
C	1.89991	0.30311	4.21918
H	1.98137	-0.70826	3.8088
H	2.84656	0.82065	4.03074
H	1.76183	0.22907	5.30385
H	3.80456	2.27144	-0.43707
H	2.84893	1.52899	0.86666
C	1.84818	2.30662	-2.78022
H	0.91914	2.73159	-3.16683
C	2.94531	3.37927	-2.87921
H	3.9208	2.99628	-2.55793
H	3.05557	3.69519	-3.92502
H	2.72274	4.27401	-2.29012
C	2.19321	1.09597	-3.66277
H	3.14803	0.64222	-3.37213
H	1.41571	0.33067	-3.59895
H	2.28662	1.416	-4.70901
C	0.67177	3.33956	-0.20602
H	0.01183	2.90681	0.55109

C	-0.19623	4.16695	-1.16901
H	-0.74844	4.92343	-0.59734
H	0.40935	4.69959	-1.91133
H	-0.92252	3.55058	-1.70212
C	1.68594	4.23236	0.52689
H	2.43171	4.66508	-0.14946
H	1.15594	5.06858	1.00194
H	2.20841	3.68603	1.31573
C	6.75179	-1.86815	-1.03416
H	7.14164	-2.06597	-0.02686
H	6.90823	-2.77247	-1.63066
H	7.35928	-1.06412	-1.46359
C	-1.33436	0.07499	-1.10898
C	-1.86333	0.92056	-2.0998
C	-2.35465	-0.53484	-0.32613
C	-3.2111	1.18999	-2.35505
C	-3.71575	-0.31589	-0.53867
H	-2.05959	-1.15486	0.51146
C	-4.15413	0.54567	-1.5437
H	-4.44236	-0.80311	0.10921
H	-5.21413	0.7276	-1.70378
F	-0.97683	1.53855	-2.96404
C	-3.62543	2.10289	-3.48568
H	-4.58874	2.57166	-3.24481
H	-2.90226	2.91671	-3.61629
Si	-3.82873	1.21582	-5.16432
H	-4.96057	0.24678	-5.03864
C	-2.27121	0.26575	-5.65862
H	-1.41238	0.93814	-5.76318
H	-2.01218	-0.48179	-4.90093
H	-2.41634	-0.25436	-6.61322
C	-4.27867	2.49104	-6.49497
H	-3.47631	3.22805	-6.62401
H	-4.44679	2.01036	-7.46627
H	-5.19257	3.03755	-6.23289

Thermal correction to Energy= 0.966465

Thermal correction to Enthalpy= 0.967409

Thermal correction to Gibbs Free Energy= 0.824274

Sum of electronic and zero-point Energies= -2817.405533

Sum of electronic and thermal Energies= -2817.350815

Sum of electronic and thermal Enthalpies= -2817.349871

Sum of electronic and thermal Free Energies= -2817.493006

SCF Done: E(wB97XD) =- -2819.32246483

TS2b

Number of Negative Frequencies = 1

B	-0.22644	1.56316	-1.67994
C	0.22447	0.76249	2.6343
C	-0.27261	0.89258	3.93106
C	-1.63751	0.72066	4.18647
C	-2.46093	0.45727	3.08602
C	-1.91504	0.33944	1.80819
C	-1.39822	3.14252	-2.97992
C	0.00076	2.84108	-3.6324
N	-0.58204	0.45082	1.58977
O	-1.497	2.10712	-1.96981
O	0.69793	2.14796	-2.56776
C	1.2224	-1.69153	-0.31783
C	1.67008	-2.19079	0.93099
C	2.26907	-3.44491	1.0839
H	2.58174	-3.77788	2.07204
C	2.45094	-4.27942	-0.01761
H	2.9153	-5.25643	0.09583
C	2.01178	-3.86999	-1.28272
C	1.42049	-2.60339	-1.36202
C	-0.06933	1.87337	-4.82492
H	0.94887	1.57458	-5.09389
H	-0.62368	0.96643	-4.57081
H	-0.53555	2.33622	-5.70235
C	0.80299	4.07727	-4.0464
H	1.78573	3.76664	-4.41729
H	0.29575	4.61841	-4.85456
H	0.95912	4.76913	-3.21628
C	-2.59544	3.02492	-3.92674
H	-3.52318	3.20583	-3.37247
H	-2.53582	3.76809	-4.73109
H	-2.66101	2.03277	-4.3793
C	-1.46504	4.49418	-2.2525
H	-1.47189	5.33611	-2.95413
H	-2.38686	4.5332	-1.66241
H	-0.62197	4.62151	-1.56799
H	1.50384	-1.60935	1.83106
H	0.41011	1.1335	4.74163
H	-3.53542	0.35996	3.21647
H	1.12147	-0.49984	-1.2516
C	-2.76034	0.14383	0.57853
C	1.67502	1.01489	2.29506
H	2.1208	1.72296	2.99892
C	-2.19619	0.81277	5.58417

H	-1.57966	1.45524	6.22125
H	-3.21715	1.20888	5.58243
H	-2.23217	-0.17819	6.05629
Co	0.20901	0.16344	-0.32014
H	2.25619	0.08879	2.36378
P	1.75236	1.55145	0.49707
C	1.32285	3.4162	0.6046
H	0.27422	3.41355	0.28809
C	2.11207	4.27172	-0.40072
H	1.66706	5.27403	-0.457
H	3.15558	4.40236	-0.09276
H	2.0936	3.83737	-1.40201
C	1.374	4.05553	2.00284
H	2.38594	4.06424	2.42424
H	1.04666	5.1011	1.9316
H	0.71077	3.55676	2.71609
C	3.59344	1.53104	0.08298
H	3.66346	2.19862	-0.78399
C	4.49536	2.08785	1.19848
H	5.53191	2.14786	0.8427
H	4.20088	3.08945	1.52595
H	4.49281	1.4316	2.07667
C	4.08471	0.14895	-0.37228
H	5.13761	0.22015	-0.67523
H	4.01514	-0.59922	0.42376
H	3.50507	-0.22003	-1.2212
H	-2.90649	1.12101	0.09775
H	-3.74375	-0.26218	0.83152
P	-1.80748	-0.84639	-0.69068
C	-1.95347	-2.63149	-0.04753
C	-2.91996	-0.67123	-2.20235
C	-4.37432	-1.14337	-2.0465
H	-4.86862	-0.69894	-1.1758
H	-4.95199	-0.85006	-2.9334
H	-4.45014	-2.23248	-1.95902
C	-1.83994	-3.68236	-1.16646
H	-2.77091	-3.76266	-1.73878
H	-1.02922	-3.46092	-1.86287
H	-1.64165	-4.66669	-0.72459
C	-2.2777	-1.25	-3.47641
H	-2.37798	-2.3382	-3.52807
H	-2.77647	-0.83508	-4.36219
H	-1.21202	-1.01249	-3.53756
H	-2.92193	0.4177	-2.3103
C	-3.16436	-2.93377	0.85205
H	-4.11905	-2.80338	0.3308
H	-3.11493	-3.97944	1.18402
H	-3.1786	-2.31126	1.75232
H	-1.0519	-2.72006	0.56797
C	2.17344	-4.73752	-2.50681
H	1.32246	-4.60609	-3.18565
H	2.18538	-5.79511	-2.21166
Si	3.77028	-4.40923	-3.50385
H	4.93314	-4.78723	-2.64254
C	3.77705	-5.52151	-5.03941
H	4.70343	-5.40004	-5.61369
H	3.69227	-6.5805	-4.76749
H	2.93984	-5.28129	-5.70644
C	3.94064	-2.59285	-3.99435
H	3.97521	-1.9487	-3.10917
H	4.86079	-2.4274	-4.56783
H	3.09297	-2.26425	-4.60583
F	0.98709	-2.24688	-2.62425

Thermal correction to Energy= 0.966205

Thermal correction to Enthalpy= 0.967149

Thermal correction to Gibbs Free Energy= 0.822648

Sum of electronic and zero-point Energies= -2817.406773

Sum of electronic and thermal Energies= -2817.351630

Sum of electronic and thermal Enthalpies= -2817.350686

Sum of electronic and thermal Free Energies= -2817.495187

SCF Done: E(wB97XD) = -2819.32553874

Int3b

Number of Negative Frequencies = 0

B	-0.20191	1.07088	-2.01177
C	0.70121	0.24491	2.62379
C	0.64001	0.0035	3.99461
C	-0.58135	-0.28458	4.61216
C	-1.719	-0.25819	3.79939
C	-1.61062	0.01096	2.43583
C	-0.91861	1.78218	-4.11942
C	0.59791	2.14837	-3.93184
N	-0.40937	0.20817	1.83662
O	-1.15136	0.8636	-3.02614
O	0.78077	1.95073	-2.50941
C	0.74552	-1.69892	-0.56391
C	0.57542	-2.54871	0.54573
C	1.05709	-3.86099	0.56922

H	0.90536	-4.47642	1.45505
C	1.71272	-4.39291	-0.53874
H	2.06296	-5.42269	-0.53089
C	1.91082	-3.60939	-1.68424
C	1.43312	-2.29359	-1.62519
C	1.55054	1.19061	-4.66306
H	2.57748	1.40058	-4.34597
H	1.33216	0.14912	-4.41803
H	1.49702	1.3196	-5.75018
C	0.96728	3.59381	-4.27302
H	2.03244	3.75616	-4.07507
H	0.78841	3.80726	-5.33384
H	0.40142	4.31146	-3.67409
C	-1.25702	1.07424	-5.43285
H	-2.32636	0.83896	-5.46163
H	-1.03076	1.71318	-6.29509
H	-0.70326	0.13842	-5.53702
C	-1.85826	2.98337	-3.92223
H	-1.76702	3.71123	-4.73632
H	-2.89378	2.62961	-3.89912
H	-1.65324	3.49377	-2.97572
H	0.05196	-2.19393	1.43068
H	1.5563	0.0375	4.57756
H	-2.70365	-0.41992	4.22947
H	0.22293	-0.342	-1.43309
C	-2.82012	0.22133	1.57181
C	1.98187	0.60428	1.92275
H	2.69391	1.06632	2.61331
C	-0.6669	-0.60582	6.08258
H	-0.51457	-1.6795	6.25633
H	0.09971	-0.07263	6.65462
H	-1.64727	-0.34447	6.49401
Co	-0.21973	0.32187	-0.17683
H	2.44325	-0.30263	1.51158
P	1.49584	1.6244	0.42558
C	0.95653	3.29364	1.15933
H	-0.09265	3.08293	1.39924
C	0.95974	4.38638	0.07691
H	0.429	5.2761	0.43938
H	1.97815	4.69758	-0.18354
H	0.46968	4.03979	-0.83799
C	1.63456	3.7671	2.45585
H	2.70218	3.96924	2.32934
H	1.16481	4.70057	2.79426

H	1.52273	3.03742	3.26468
C	3.12659	1.93636	-0.46061
H	2.82948	2.58417	-1.29145
C	4.22137	2.638	0.35912
H	5.13028	2.7323	-0.24974
H	3.93476	3.6453	0.67237
H	4.49323	2.06782	1.25582
C	3.66192	0.63125	-1.06845
H	4.54707	0.8476	-1.68069
H	3.97363	-0.07857	-0.29214
H	2.92098	0.14053	-1.70081
H	-3.06188	1.29162	1.62169
H	-3.68895	-0.31689	1.96308
P	-2.43828	-0.13139	-0.22986
C	-2.90152	-1.96973	-0.33708
C	-3.8082	0.85883	-1.11498
C	-5.07389	1.16344	-0.29258
H	-4.86709	1.73278	0.61865
H	-5.76063	1.76884	-0.89887
H	-5.61067	0.25237	-0.0072
C	-2.50612	-2.59806	-1.68449
H	-3.19138	-2.30787	-2.4862
H	-1.49365	-2.31078	-1.97471
H	-2.53512	-3.69224	-1.60382
C	-4.20713	0.26252	-2.47619
H	-4.82165	-0.63616	-2.35945
H	-4.81248	0.99486	-3.0273
H	-3.3319	0.02184	-3.07996
H	-3.29477	1.80661	-1.31641
C	-4.33319	-2.36869	0.05774
H	-5.06978	-2.03747	-0.68165
H	-4.40652	-3.46276	0.11858
H	-4.63423	-1.97009	1.03266
H	-2.21599	-2.38899	0.41407
C	2.58196	-4.16486	-2.91915
H	2.26509	-3.60435	-3.80565
H	2.2614	-5.2045	-3.07264
Si	4.48942	-4.17483	-2.87388
H	4.92346	-5.01331	-1.71375
C	5.14144	-4.97421	-4.46465
H	6.23643	-5.03453	-4.45766
H	4.7544	-5.99266	-4.58887
H	4.84677	-4.3966	-5.34951
C	5.19841	-2.43661	-2.66744

H	4.87889	-1.99117	-1.71954
H	6.29491	-2.45446	-2.67896
H	4.86207	-1.77386	-3.47335
F	1.65103	-1.53814	-2.76333

Thermal correction to Energy= 0.969738

Thermal correction to Enthalpy= 0.970682

Thermal correction to Gibbs Free Energy= 0.825743

Sum of electronic and zero-point Energies= -2817.426642

Sum of electronic and thermal Energies= -2817.371674

Sum of electronic and thermal Enthalpies= -2817.370730

Sum of electronic and thermal Free Energies= -2817.515669

SCF Done: E(wB97XD) = -2819.35406747

TS4b

Number of Negative Frequencies = 1

Co	0.83362	-0.44832	-0.71865
P	0.50671	-2.66359	-0.53906
P	1.78233	1.57142	-1.28712
N	2.78731	-0.94997	-0.5155
C	2.14145	-3.15933	-1.28234
H	2.43126	-4.20195	-1.13201
H	2.01338	-2.9882	-2.35996
C	3.19857	-2.21447	-0.78657
C	4.53616	-2.59203	-0.67515
H	4.81391	-3.6187	-0.89717
C	5.50993	-1.65987	-0.30613
C	5.07789	-0.34508	-0.10578
H	5.79116	0.43665	0.1402
C	3.72877	-0.01633	-0.22625
C	3.2264	1.39166	-0.10265
H	0.73988	-0.8587	-2.25419
C	0.18995	1.64822	2.93819
C	-0.38491	0.23224	3.32971
B	0.21326	0.23999	1.05142
O	-0.03028	-0.57334	2.18162
O	0.25162	1.58481	1.49686
C	-0.77436	-3.60875	-1.54936
H	-1.66372	-3.5997	-0.90778
C	-0.38462	-5.0736	-1.81826
H	-1.21946	-5.59679	-2.30157
H	0.47376	-5.1382	-2.49733
H	-0.13395	-5.62426	-0.90649
C	-1.14835	-2.89475	-2.85579
H	-1.4829	-1.87349	-2.66668
H	-0.29917	-2.84072	-3.54553
H	-1.95495	-3.45015	-3.3533
C	0.51408	-3.51948	1.17702
H	0.7121	-2.66867	1.83494
C	1.62506	-4.56203	1.39127
H	2.62474	-4.14483	1.23367
H	1.58551	-4.92544	2.42668
H	1.51342	-5.43614	0.7383
C	-0.84273	-4.10544	1.60469
H	-1.10349	-5.00821	1.04099
H	-0.79515	-4.38751	2.66432
H	-1.66417	-3.39289	1.49731
C	-1.9124	0.2189	3.49488
H	-2.25101	-0.81648	3.60888
H	-2.22057	0.77455	4.38803
H	-2.42142	0.64124	2.62606
C	-0.6886	2.84022	3.32885
H	-0.80765	2.90924	4.41696
H	-0.22005	3.76798	2.98265
H	-1.67855	2.77545	2.87202
C	0.2504	-0.40785	4.56825
H	0.06817	0.1952	5.46596
H	-0.19232	-1.39614	4.7336
H	1.32854	-0.54037	4.44993
C	1.61698	1.8821	3.46279
H	2.28637	1.06449	3.17713
H	2.01098	2.81185	3.0385
H	1.63771	1.97744	4.55408
H	4.03773	2.11344	-0.23363
H	2.78841	1.54162	0.88926
C	2.61718	1.75481	-2.98442
H	1.80211	2.13449	-3.61383
C	3.77684	2.76701	-3.0198
H	4.64495	2.3904	-2.46581
H	4.10202	2.92219	-4.0568
H	3.51512	3.74499	-2.60767
C	3.07422	0.42033	-3.59307
H	3.95648	0.02427	-3.07625
H	2.27715	-0.32542	-3.53699
H	3.35167	0.57232	-4.64449
C	1.08973	3.31192	-0.92687
H	0.20504	3.07726	-0.33214
C	0.63459	4.08749	-2.17669
H	0.06302	4.96733	-1.85371

H	1.47837	4.45524	-2.76994
H	-0.01442	3.49734	-2.82427
C	1.99784	4.20218	-0.0616
H	2.94403	4.44981	-0.55682
H	1.48217	5.14978	0.14274
H	2.22444	3.74166	0.90263
C	6.9553	-2.05236	-0.13381
H	7.15435	-2.36237	0.9008
H	7.22271	-2.89277	-0.78271
H	7.62695	-1.21729	-0.35826
C	-1.32556	0.04211	-0.37955
C	-1.89163	0.93869	-1.28832
C	-2.2745	-0.74877	0.29832
C	-3.25869	1.13567	-1.51855
C	-3.65208	-0.61087	0.11026
H	-1.92124	-1.4565	1.03687
C	-4.14465	0.33326	-0.78699
H	-4.34065	-1.23612	0.67457
H	-5.21585	0.45551	-0.93127
F	-1.07139	1.6939	-2.08705
C	-3.75001	2.1322	-2.54156
H	-4.75348	2.47885	-2.2607
H	-3.10383	3.01781	-2.56073
Si	-3.87202	1.44033	-4.31941
H	-4.79598	0.26539	-4.28493
C	-2.19642	0.88629	-4.9942
H	-1.51473	1.7384	-5.10173
H	-1.71194	0.16192	-4.33146
H	-2.31178	0.42225	-5.98145
C	-4.62981	2.76334	-5.44798
H	-3.99524	3.65716	-5.49342
H	-4.74755	2.389	-6.47211
H	-5.61928	3.07563	-5.09324

Thermal correction to Energy= 0.966011

Thermal correction to Enthalpy= 0.966955

Thermal correction to Gibbs Free Energy= 0.826801

Sum of electronic and zero-point Energies= -2817.407042

Sum of electronic and thermal Energies= -2817.352914

Sum of electronic and thermal Enthalpies= -2817.351970

Sum of electronic and thermal Free Energies= -2817.492124

SCF Done: E(wB97XD) = -2819.32640892

Int5b

Number of Negative Frequencies = 0

Co	-0.00273	-0.77362	-0.01467
P	-2.15706	-0.67343	0.0013
P	2.14696	-0.67825	-0.0968
N	-0.01042	1.16936	-0.02681
C	-2.37523	1.08159	0.63082
H	-3.2904	1.59178	0.30677
H	-2.42797	1.00762	1.72595
C	-1.14753	1.87941	0.25279
C	-1.17542	3.27156	0.23068
H	-2.10747	3.7823	0.45905
C	-0.02315	4.00999	-0.05527
C	1.13734	3.27535	-0.32326
H	2.06673	3.78954	-0.55541
C	1.12152	1.88371	-0.31964
C	2.35768	1.09208	-0.68683
H	0.01034	-2.31911	-0.02688
C	-3.24222	-1.74369	1.11477
H	-3.51677	-2.5938	0.47358
C	-4.53233	-1.09661	1.64711
H	-5.10788	-1.83295	2.22319
H	-4.30798	-0.26498	2.3257
H	-5.18093	-0.71542	0.85451
C	-2.39039	-2.28745	2.27474
H	-1.48008	-2.76381	1.90152
H	-2.08836	-1.47913	2.95358
H	-2.96655	-3.01428	2.86282
C	-2.99175	-0.65478	-1.69226
H	-2.4559	0.16344	-2.19531
C	-4.49526	-0.34839	-1.74739
H	-4.76218	0.56321	-1.20099
H	-4.81065	-0.20953	-2.79001
H	-5.0883	-1.17469	-1.3394
C	-2.66318	-1.95532	-2.44549
H	-3.15155	-2.8209	-1.97979
H	-3.01997	-1.89668	-3.48206
H	-1.58584	-2.14375	-2.45006
H	3.26634	1.6009	-0.34237
H	2.42743	1.03824	-1.78221
C	3.11104	-0.78246	1.52369
H	2.99342	-1.83434	1.81746
C	4.61099	-0.45576	1.48249
H	4.80077	0.55018	1.08836
H	5.02896	-0.48845	2.49753
H	5.17543	-1.16824	0.8751

C	2.38981	0.07448	2.57954
H	2.45432	1.14344	2.34011
H	1.33	-0.19023	2.64532
H	2.85115	-0.07091	3.56492
C	3.06372	-1.71666	-1.3765
H	2.32825	-1.72895	-2.19298
C	3.20527	-3.16399	-0.87636
H	3.55221	-3.81264	-1.69064
H	3.93802	-3.24094	-0.06338
H	2.24571	-3.54786	-0.51569
C	4.38599	-1.17895	-1.94857
H	4.70285	-1.80664	-2.79206
H	4.29431	-0.15388	-2.32399
C	-0.03395	5.51707	-0.09985
H	-0.15916	5.88284	-1.12831
H	-0.85551	5.92963	0.495
H	0.90496	5.93614	0.27896
H	5.19559	-1.19393	-1.21292

Thermal correction to Energy= 0.575103

Thermal correction to Enthalpy= 0.576047

Thermal correction to Gibbs Free Energy= 0.480981

Sum of electronic and zero-point Energies=-1667.007495

Sum of electronic and thermal Energies=-1666.976026

Sum of electronic and thermal Enthalpies=-1666.975081

Sum of electronic and thermal Free Energies=-1667.070148

SCF Done: E(wB97XD) = -1668.433234

TS6b

Number of Negative Frequencies = 1

Co	0.91488	-0.50448	-1.15243
P	0.49294	-2.6609	-1.02905
P	1.71714	1.47576	-1.69089
N	2.7746	-0.93827	-0.6772
C	2.26422	-3.27489	-1.17911
H	2.46207	-4.25359	-0.73287
H	2.44877	-3.38015	-2.25753
C	3.2146	-2.23057	-0.65149
C	4.50322	-2.56246	-0.24181
H	4.79818	-3.60845	-0.22622
C	5.42025	-1.56667	0.11343
C	4.98328	-0.24357	0.00043
H	5.66022	0.57718	0.22269
C	3.67708	0.04644	-0.39293
C	3.19308	1.46653	-0.53484
C	-3.63284	0.07011	-0.63148
C	-3.35944	1.61712	-0.50273
B	-1.48721	0.42722	0.15169
O	-2.53621	-0.49552	0.12393
O	-1.92766	1.6729	-0.31353
H	-0.50339	-0.16649	-1.62839
C	0.98401	1.17276	3.03824
C	0.76542	-0.37093	3.27078
B	-0.20926	0.28948	1.26552
O	-0.19957	-0.7029	2.24342
O	0.64836	1.33332	1.63622
C	-0.35694	-3.46882	-2.50661
H	-1.42157	-3.27608	-2.32284
C	-0.15321	-4.98494	-2.65724
H	-0.69775	-5.35174	-3.5375
H	0.90425	-5.23702	-2.80452
H	-0.51428	-5.54636	-1.79082
C	0.02318	-2.72959	-3.80007
H	-0.13966	-1.65318	-3.69494
H	1.07736	-2.88889	-4.0621
H	-0.5788	-3.10317	-4.63917
C	-0.20982	-3.53769	0.50604
H	-0.06138	-2.78069	1.28218
C	0.50453	-4.82292	0.95376
H	1.55428	-4.6462	1.21264
H	0.01383	-5.21525	1.85447
H	0.47235	-5.61482	0.1969
C	-1.7302	-3.74628	0.39521
H	-1.99448	-4.50609	-0.34996
H	-2.11775	-4.09117	1.36262
H	-2.23889	-2.80971	0.15326
C	0.15909	-0.73928	4.62728
H	0.018	-1.82382	4.68268
H	0.82191	-0.4425	5.44901
H	-0.81516	-0.26848	4.77638
C	-0.0011	2.05371	3.82436
H	0.20688	2.04039	4.89999
H	0.08603	3.08514	3.46828
H	-1.03468	1.73143	3.66508
C	2.02503	-1.21527	3.03051
H	2.77493	-1.05016	3.8124
H	1.75033	-2.27524	3.04578
H	2.47591	-0.99703	2.05978
C	2.41075	1.66711	3.28051

H	3.13924	1.13781	2.66203
H	2.47508	2.73581	3.04805
H	2.69354	1.53643	4.33182
H	4.01998	2.13643	-0.79776
H	2.77463	1.81056	0.41805
C	2.44081	1.61595	-3.43505
H	1.546	1.70396	-4.06623
C	3.34617	2.826	-3.71335
H	4.21653	2.84554	-3.0461
H	3.72873	2.77688	-4.74161
H	2.81842	3.77713	-3.60662
C	3.15145	0.30979	-3.82989
H	4.07014	0.1626	-3.24927
H	2.50606	-0.55745	-3.66444
H	3.43338	0.34207	-4.89052
C	0.85295	3.11314	-1.3456
H	0.21449	2.84981	-0.4968
C	-0.08095	3.48395	-2.50816
H	-0.73831	4.30835	-2.20602
H	0.47297	3.81373	-3.39551
H	-0.71866	2.6402	-2.7869
C	1.73401	4.29857	-0.91849
H	2.40245	4.64226	-1.71424
H	1.09247	5.14613	-0.64306
H	2.34717	4.05982	-0.04348
C	-3.99253	2.24289	0.75159
H	-3.74022	1.66781	1.64816
H	-3.59636	3.25566	0.87859
H	-5.08376	2.3069	0.6743
C	-3.73532	2.44578	-1.73206
H	-4.81385	2.3954	-1.92465
H	-3.47263	3.49548	-1.56228
H	-3.20635	2.10629	-2.62521
C	-3.53084	-0.44689	-2.07428
H	-3.57959	-1.54126	-2.06271
H	-4.35397	-0.077	-2.69599
H	-2.57813	-0.15854	-2.52605
C	-4.94299	-0.40855	-0.00206
H	-5.80819	0.05338	-0.49275
H	-5.03074	-1.49421	-0.11776
H	-4.98423	-0.18172	1.06583
C	6.80904	-1.90658	0.59163
H	6.81249	-2.11958	1.66927
H	7.20078	-2.79575	0.0857
H	7.50609	-1.07992	0.41983

Thermal correction to Energy= 0.962771

Thermal correction to Enthalpy= 0.963716

Thermal correction to Gibbs Free Energy= 0.828395

Sum of electronic and zero-point Energies= -2489.173829

Sum of electronic and thermal Energies= -2489.122083

Sum of electronic and thermal Enthalpies= -2489.121138

Sum of electronic and thermal Free Energies= -2489.256459

SCF Done: E(wB97XD) = -2491.076863

Int7b

Number of Negative Frequencies = 0

Co	-0.6992	0.07697	-0.0913
P	-0.56255	2.28551	0.17235
P	-1.12522	-2.0431	-0.66123
N	-2.62505	0.42453	-0.61885
C	-2.38182	2.70639	0.21399
H	-2.67653	2.63731	1.2721
H	-2.6174	3.72478	-0.1156
C	-3.17645	1.67609	-0.54038
C	-4.44694	1.96653	-1.02874
H	-4.81373	2.98798	-0.9643
C	-5.25295	0.96248	-1.57673
C	-4.74068	-0.33836	-1.51521
H	-5.34528	-1.18061	-1.8425
C	-3.46662	-0.58264	-1.0136
C	-2.98965	-1.98813	-0.76423
C	3.89078	0.64851	-1.57606
C	3.77539	-0.911	-1.35991
B	1.68825	0.03804	-1.39989
O	2.54724	1.10952	-1.2665
O	2.35839	-1.15153	-1.5925
H	0.51224	0.16917	-1.62496
C	1.57006	-0.54211	3.53903
C	0.09726	-0.98911	3.87169
B	0.23205	-0.36603	1.60222
O	-0.4964	-1.11027	2.56263
O	1.43783	0.03435	2.21444
C	0.0579	3.27603	-1.32426
H	1.10105	2.95868	-1.42535
C	0.01882	4.80886	-1.20522
H	0.35775	5.25487	-2.14983
H	-0.99597	5.18406	-1.02195
H	0.66828	5.19048	-0.41509

C	-0.69351	2.84405	-2.59642
H	-0.6886	1.75946	-2.72584
H	-1.7385	3.17492	-2.57861
H	-0.2209	3.29619	-3.47715
C	0.12735	3.14098	1.71132
H	-0.08248	2.38643	2.47755
C	-0.53449	4.45351	2.16231
H	-1.61923	4.35625	2.28054
H	-0.12838	4.74511	3.13974
H	-0.34578	5.28222	1.4734
C	1.65858	3.26799	1.62813
H	1.96837	4.00902	0.88297
H	2.05412	3.5948	2.59917
H	2.11701	2.30918	1.38079
C	-0.714	0.06275	4.64568
H	-1.76216	-0.2524	4.67417
H	-0.3607	0.17863	5.67793
H	-0.67497	1.04128	4.15645
C	2.15328	0.52053	4.47611
H	2.24008	0.13904	5.50151
H	3.15644	0.79801	4.13614
H	1.5445	1.42754	4.49482
C	-0.02558	-2.336	4.59301
H	0.45115	-2.30448	5.57973
H	-1.08448	-2.57462	4.73905
H	0.42546	-3.1466	4.01646
C	2.55634	-1.71656	3.44507
H	2.20148	-2.48018	2.74652
H	3.5209	-1.35114	3.0823
H	2.71868	-2.18763	4.42157
H	-3.42255	-2.69855	-1.47841
H	-3.3489	-2.2817	0.23154
C	-0.61691	-2.54272	-2.41961
H	0.47308	-2.42563	-2.41707
C	-0.95546	-3.97745	-2.85337
H	-2.03004	-4.18443	-2.7898
H	-0.66025	-4.12118	-3.90229
H	-0.43067	-4.73141	-2.26147
C	-1.19968	-1.55135	-3.44561
H	-2.28652	-1.66347	-3.54044
H	-0.99081	-0.51263	-3.1812
H	-0.76478	-1.74471	-4.43404
C	-0.79558	-3.51708	0.46638
H	-0.94392	-3.04285	1.44426
C	0.68292	-3.93088	0.38988
H	0.93208	-4.59199	1.22972
H	0.90915	-4.47967	-0.53312
H	1.344	-3.06008	0.42808
C	-1.73439	-4.73273	0.38542
H	-1.61984	-5.29723	-0.5434
H	-1.50653	-5.41832	1.21299
H	-2.7878	-4.45311	0.48059
C	4.0758	-1.34289	0.07875
H	3.4735	-0.77441	0.78996
H	3.8217	-2.40155	0.19126
H	5.13798	-1.22051	0.32289
C	4.57286	-1.76381	-2.3473
H	5.64803	-1.57772	-2.24586
H	4.39363	-2.82482	-2.1396
H	4.28184	-1.57052	-3.38168
C	4.16522	1.04583	-3.03421
H	4.03863	2.128	-3.13702
H	5.1839	0.78891	-3.34191
H	3.4615	0.55755	-3.71635
C	4.87525	1.36142	-0.64919
H	5.89794	1.00291	-0.81169
H	4.85916	2.43806	-0.85396
H	4.61462	1.215	0.40197
C	-6.60319	1.25998	-2.17605
H	-6.52785	1.44457	-3.25706
H	-7.29692	0.42303	-2.03927
H	-7.05378	2.15235	-1.72811

Thermal correction to Energy= 0.963954

Thermal correction to Enthalpy= 0.964898

Thermal correction to Gibbs Free Energy= 0.828136

Sum of electronic and zero-point Energies= -2489.181721

Sum of electronic and thermal Energies= -2489.129455

Sum of electronic and thermal Enthalpies= -2489.128511

Sum of electronic and thermal Free Energies= -2489.265273

SCF Done: E(wB97XD) = -2491.091817

TS8b

Number of Negative Frequencies = 1

Co	-0.3062	0.09307	-0.12325
P	-0.16247	2.28043	0.12074
P	-0.67172	-2.02466	-0.62093
N	-2.30637	0.36479	-0.32929
C	-1.97799	2.68779	0.35012

H	-2.15307	2.70976	1.43503	H	-1.81244	1.20015	3.95871
H	-2.26164	3.67415	-0.03319	C	0.70218	0.43801	5.21857
C	-2.85649	1.59947	-0.21946	H	0.45645	0.01516	6.20054
C	-4.19624	1.834	-0.5264	H	1.77658	0.64991	5.20246
H	-4.58882	2.84437	-0.44767	H	0.17254	1.38748	5.10871
C	-5.02976	0.78156	-0.91668	C	-1.60445	-2.20088	4.60178
C	-4.46934	-0.49895	-0.91458	H	-1.45621	-2.1721	5.68826
H	-5.08164	-1.36579	-1.14846	H	-2.67022	-2.37065	4.41289
C	-3.12367	-0.68344	-0.59845	H	-1.05187	-3.05457	4.20147
C	-2.53428	-2.06279	-0.43943	C	1.26043	-1.77943	4.20725
C	4.04978	0.69206	-2.16204	H	1.05179	-2.49867	3.40993
C	4.07242	-0.87199	-1.99558	H	2.30398	-1.46458	4.10642
B	2.12859	-0.01593	-1.1096	H	1.14396	-2.28493	5.17298
O	2.95511	1.07848	-1.27474	H	-3.03664	-2.79356	-1.08291
O	2.68655	-1.16351	-1.64041	H	-2.71008	-2.3632	0.60234
H	0.2216	0.28164	-1.61399	C	-0.35394	-2.53654	-2.41559
C	0.35752	-0.54083	4.09366	H	0.70152	-2.27659	-2.55616
C	-1.16878	-0.8868	3.95038	C	-0.54804	-4.02026	-2.76341
B	-0.29223	-0.29031	1.85663	H	-1.56423	-4.36797	-2.53925
O	-1.32424	-1.00029	2.51475	H	-0.38775	-4.16609	-3.84003
O	0.65142	0.0888	2.82364	H	0.1575	-4.6699	-2.23956
C	0.31535	3.27507	-1.41945	C	-1.18878	-1.66379	-3.36921
H	1.30243	2.871	-1.66959	H	-2.25381	-1.92545	-3.32624
C	0.42751	4.79891	-1.25515	H	-1.07957	-0.60269	-3.13146
H	0.65933	5.25474	-2.22711	H	-0.85704	-1.82352	-4.40333
H	-0.51217	5.24624	-0.90721	C	-0.12225	-3.43624	0.50516
H	1.21974	5.09121	-0.56177	H	-0.30785	-2.97287	1.4801
C	-0.64002	2.94822	-2.58083	C	1.39091	-3.67513	0.38336
H	-0.75612	1.86878	-2.70558	H	1.72918	-4.34866	1.18179
H	-1.63177	3.39144	-2.42325	H	1.6562	-4.13997	-0.57327
H	-0.24375	3.36595	-3.51542	H	1.94474	-2.73564	0.46005
C	0.65876	3.08919	1.61587	C	-0.91848	-4.75283	0.48105
H	0.43376	2.3476	2.38954	H	-0.73427	-5.34534	-0.41821
C	0.12649	4.45058	2.09626	H	-0.61981	-5.36787	1.34069
H	-0.96036	4.45355	2.23591	H	-1.99852	-4.5896	0.56259
H	0.57654	4.68644	3.06995	C	4.94146	-1.34877	-0.82327
H	0.37822	5.26967	1.41778	H	4.69855	-0.80631	0.09546
C	2.18907	3.09456	1.46249	H	4.74735	-2.41156	-0.64765
H	2.52688	3.81593	0.70947	H	6.0099	-1.22416	-1.03237
H	2.65258	3.3733	2.41766	C	4.42123	-1.65805	-3.25865
H	2.56181	2.10898	1.17357	H	5.43772	-1.42524	-3.5981
C	-2.09818	0.24655	4.41407	H	4.37476	-2.73157	-3.04733
H	-3.12134	0.0167	4.09882	H	3.72501	-1.4448	-4.07319
H	-2.09233	0.36631	5.50352	C	3.66032	1.14922	-3.57482

H	3.47924	2.22884	-3.56196
H	4.45375	0.94478	-4.30249
H	2.74223	0.657	-3.90975
C	5.31555	1.41575	-1.70674
H	6.17998	1.11141	-2.30917
H	5.18426	2.4963	-1.82681
H	5.53605	1.2195	-0.65521
C	-6.46711	1.01505	-1.30897
H	-6.89018	1.87963	-0.78668
H	-6.55141	1.21147	-2.38627
H	-7.09006	0.14209	-1.08764

Thermal correction to Energy= 0.964626

Thermal correction to Enthalpy= 0.965570

Thermal correction to Gibbs Free Energy= 0.830531

Sum of electronic and zero-point Energies= -2489.161214

Sum of electronic and thermal Energies= -2489.109302

Sum of electronic and thermal Enthalpies= -2489.108358

Sum of electronic and thermal Free Energies= -2489.243397

SCF Done: E(wB97XD) = -2491.062552

cat1'

Number of Negative Frequencies =0

B	-1.42714	-2.00354	1.19522
B	-2.18584	-0.64847	-1.09533
B	-1.82435	0.68416	1.3064
C	1.44748	0.34105	2.50591
H	0.54936	0.84203	2.84555
C	2.62782	0.40258	3.24206
C	3.77638	-0.24137	2.77084
C	3.64983	-0.91298	1.54665
C	2.43648	-0.94087	0.85512
C	2.26369	-1.63524	-0.4501
C	3.29741	-2.33499	-1.07628
C	3.10334	-2.97354	-2.30974
C	1.82445	-2.86377	-2.86362
C	0.82898	-2.15568	-2.19237
H	-0.17858	-2.06579	-2.58687
C	-2.84447	-3.65544	1.95701
C	-1.51195	-3.66362	2.7998
C	-4.28611	-0.50936	-2.08018
C	-3.2792	-1.16536	-3.09371
C	-2.41789	2.66434	2.38456
C	-3.57557	1.60276	2.51823
Ir	-0.61277	-0.49039	0.18618
N	1.33825	-0.31779	1.34593
N	1.03203	-1.55259	-1.01201
O	-2.48015	-2.83528	0.82092
O	-0.9025	-2.40649	2.42772
O	-3.39629	0.05375	-1.09264
O	-2.15656	-1.47659	-2.23971
O	-1.55589	2.06302	1.3917
O	-2.91813	0.37891	2.12269
C	5.11876	-0.23512	3.5181
C	4.25289	-3.7452	-2.97568
C	-3.29537	-5.02332	1.44285
H	-3.5258	-5.70063	2.27424
H	-4.20394	-4.90735	0.84246
H	-2.53451	-5.48989	0.81234
C	-4.00992	-2.95232	2.66923
H	-4.83797	-2.84177	1.9615
H	-4.36769	-3.52921	3.52994
H	-3.71996	-1.95032	2.99577
C	-1.70428	-3.67961	4.31648
H	-2.22243	-4.59045	4.64027
H	-0.7274	-3.65286	4.81194
H	-2.27696	-2.81266	4.65344
C	-0.53721	-4.77752	2.38455
H	0.43064	-4.59694	2.86433
H	-0.8917	-5.76915	2.68814
H	-0.38273	-4.77957	1.30078
C	-4.13212	1.42287	3.93265
H	-4.90182	0.64367	3.9248
H	-3.35599	1.11884	4.63968
H	-4.5946	2.34828	4.29741
C	-4.73239	1.83869	1.53513
H	-5.40732	0.9774	1.5758
H	-5.30576	2.73817	1.78825
H	-4.36376	1.91671	0.50974
C	-1.58926	2.8214	3.66976
H	-0.69139	3.4069	3.44376
H	-2.14671	3.33946	4.4584
H	-1.27608	1.84586	4.05671
C	-2.85076	4.0425	1.88141
H	-3.5492	4.51976	2.57977
H	-1.97269	4.69069	1.78531
H	-3.32788	3.9758	0.90117
C	-2.7782	-0.18637	-4.16786

H	-1.94082	-0.64398	-4.70614
H	-3.55868	0.05774	-4.89749
H	-2.42155	0.74387	-3.71465
C	-3.76384	-2.45763	-3.75302
H	-4.65953	-2.28122	-4.36104
H	-2.98303	-2.85152	-4.41375
H	-3.99174	-3.22314	-3.00789
C	-5.15292	0.61085	-2.65776
H	-5.80077	0.23664	-3.46001
H	-5.7948	1.01988	-1.87031
H	-4.5455	1.42896	-3.05312
C	-5.16977	-1.53925	-1.35867
H	-5.71105	-1.03396	-0.55208
H	-5.9031	-1.99391	-2.03499
H	-4.55678	-2.32473	-0.90794
H	1.57608	-3.32376	-3.81252
H	2.62723	0.95431	4.17408
H	4.268	-2.38972	-0.59879
H	4.51202	-1.42005	1.13161
C	5.04193	0.55186	4.84004
H	4.30471	0.12278	5.52787
H	6.01576	0.52361	5.34083
H	4.78693	1.60478	4.67485
C	6.19931	0.41797	2.62306
H	6.33427	-0.12392	1.68084
H	5.93617	1.4535	2.37963
H	7.16427	0.42571	3.14341
C	5.52725	-1.6924	3.84204
H	6.48671	-1.70506	4.37244
H	4.77984	-2.17809	4.47932
H	5.64038	-2.2987	2.93689
C	4.69906	-4.8969	-2.04275
H	5.52459	-5.45365	-2.50151
H	5.04588	-4.52673	-1.07212
H	3.87636	-5.59706	-1.85959
C	3.83505	-4.35029	-4.32927
H	3.53011	-3.57811	-5.04464
H	4.6816	-4.89038	-4.76713
H	3.00995	-5.06309	-4.22082
C	5.44373	-2.78695	-3.21722
H	5.15758	-1.9608	-3.8778
H	5.81948	-2.3551	-2.28355
H	6.27197	-3.32856	-3.68915

Thermal correction to Energy= 0.988315

Thermal correction to Enthalpy= 0.989259

Thermal correction to Gibbs Free Energy= 0.845661

Sum of electronic and zero-point Energies= -2147.542123

Sum of electronic and thermal Energies= -2147.488383

Sum of electronic and thermal Enthalpies=-2147.487438

Sum of electronic and thermal Free Energies= -2147.631037

SCF Done: E(wB97XD) = -2148.408633

TS2a′

Number of Negative Frequencies = 1

B	1.66523	1.18573	0.96275
B	2.17415	-0.29757	-1.47112
B	1.93928	-1.42556	1.15771
C	-1.4174	-1.14974	2.25893
H	-0.64637	-1.89049	2.44342
C	-2.61961	-1.15191	2.96027
C	-3.60278	-0.19731	2.67295
C	-3.27464	0.74974	1.6964
C	-2.04537	0.70857	1.03159
C	-1.67384	1.6748	-0.02743
C	-2.39212	2.85262	-0.2553
C	-2.03787	3.73983	-1.2778
C	-0.91774	3.37962	-2.03725
C	-0.2198	2.21042	-1.75368
H	0.67525	1.93334	-2.30023
C	3.11753	2.89065	1.53922
C	2.0087	2.74006	2.64582
C	4.14374	-0.78067	-2.59202
C	3.41898	0.41825	-3.31366
C	2.48648	-2.82689	2.92788
C	3.79265	-2.31658	2.21752
Ir	0.66539	-0.35664	-0.08816
N	-1.1348	-0.25667	1.29998
N	-0.5818	1.362	-0.77479
O	2.57284	2.10302	0.45153
O	1.40526	1.46767	2.30594
O	3.10355	-1.30578	-1.73961
O	2.39083	0.76801	-2.36004
O	1.46748	-2.47858	1.95716
O	3.2917	-1.22219	1.41283
H	0.58547	-1.99452	-0.41435
C	-2.24987	-1.03145	-2.18821
C	3.34842	4.31596	1.03598
H	3.71606	4.96518	1.84003

H	4.10107	4.30453	0.24065	H	6.14559	0.05418	-2.24719
H	2.43466	4.7522	0.62506	H	4.95313	0.44601	-0.97939
C	4.45684	2.24886	1.92905	Si	-0.75618	-2.07325	-1.49976
H	5.11755	2.25178	1.05585	C	0.11419	-2.80269	-3.02545
H	4.95487	2.79756	2.73663	H	0.3633	-2.02108	-3.75241
H	4.31452	1.20847	2.23263	H	1.05167	-3.29171	-2.73964
C	2.52394	2.66784	4.08348	H	-0.52362	-3.53991	-3.52925
H	3.03674	3.59473	4.36762	C	-1.51099	-3.53335	-0.53354
H	1.68143	2.52452	4.76876	H	-2.16364	-3.19157	0.2773
H	3.21474	1.83256	4.2203	H	-2.10875	-4.17429	-1.19251
C	0.89963	3.79956	2.54514	H	-0.71907	-4.1466	-0.08751
H	0.07766	3.51559	3.21066	H	-0.55921	4.00111	-2.84904
H	1.2537	4.79381	2.84024	H	-2.77713	-1.92	3.70776
H	0.5042	3.85857	1.52611	C	-4.98403	-0.17205	3.34378
C	4.87591	-1.78285	3.15501	H	-3.23192	3.0852	0.38803
H	5.72125	-1.41214	2.56578	H	-4.0006	1.50445	1.42032
H	4.50773	-0.95916	3.77158	C	-2.84833	5.02342	-1.5119
H	5.24732	-2.57437	3.81705	C	-2.2923	5.8521	-2.68545
C	4.40053	-3.34651	1.25364	H	-2.89824	6.75527	-2.81681
H	5.18218	-2.85852	0.66387	H	-1.2592	6.17098	-2.50687
H	4.84742	-4.19138	1.78978	H	-2.32176	5.29488	-3.62863
H	3.6481	-3.7263	0.55656	C	-4.31542	4.64837	-1.8313
C	2.1644	-2.06298	4.2214	H	-4.3761	4.02815	-2.73263
H	1.16204	-2.34371	4.56275	H	-4.7839	4.09398	-1.0111
H	2.87329	-2.30194	5.02207	H	-4.90824	5.55494	-2.00153
H	2.17195	-0.98194	4.05198	C	-2.80552	5.89567	-0.23419
C	2.42382	-4.33347	3.17803	H	-1.77665	6.17837	0.01529
H	3.21983	-4.65297	3.86136	H	-3.38393	6.81463	-0.38613
H	1.46321	-4.59207	3.63683	H	-3.22918	5.37536	0.63142
H	2.51458	-4.89923	2.24788	C	-6.07611	-0.26416	2.24952
C	2.72156	0.01467	-4.62126	H	-7.06822	-0.32324	2.71273
H	2.08207	0.83934	-4.9547	H	-6.07065	0.61234	1.59284
H	3.44253	-0.20045	-5.4181	H	-5.93069	-1.1478	1.61949
H	2.0916	-0.86722	-4.47745	C	-5.14791	1.15006	4.1307
C	4.28828	1.65394	-3.55813	H	-6.14011	1.19307	4.59564
H	5.13009	1.42193	-4.22187	H	-4.39672	1.23294	4.92447
H	3.68895	2.43623	-4.0374	H	-5.04622	2.02591	3.48067
H	4.67909	2.05794	-2.62198	C	-5.17335	-1.34812	4.32009
C	4.63022	-1.89885	-3.51577	H	-5.08992	-2.31553	3.81216
H	5.39516	-1.53428	-4.21228	H	-4.4431	-1.32364	5.13691
H	5.07571	-2.69852	-2.91463	H	-6.17085	-1.2949	4.7698
H	3.81075	-2.33272	-4.09416	H	-2.59352	-0.34054	-1.41689
C	5.29124	-0.32668	-1.67577	H	-1.86348	-0.42608	-3.01651
H	5.62695	-1.18239	-1.08217	C	-3.40336	-1.87308	-2.65599

C	-3.51132	-2.36767	-3.96605
C	-4.44037	-2.22434	-1.78325
C	-4.58722	-3.16196	-4.36763
H	-2.73224	-2.1141	-4.68097
C	-5.52521	-3.00776	-2.15008
C	-5.59834	-3.48464	-3.46076
H	-4.63654	-3.52443	-5.39073
H	-6.29035	-3.23315	-1.41374
H	-6.43934	-4.10069	-3.76591
F	-4.38486	-1.7678	-0.49924

Thermal correction to Energy= 1.195987

Thermal correction to Enthalpy= 1.196931

Thermal correction to Gibbs Free Energy= 1.026142

Sum of electronic and zero-point Energies= -2887.485226

Sum of electronic and thermal Energies= -2887.418268

Sum of electronic and thermal Enthalpies= -2887.417323

Sum of electronic and thermal Free Energies= -2887.588112

SCF Done: E(wB97XD) = -2888.626867

Int3a′

Number of Negative Frequencies = 0

B	1.68131	1.15536	0.98234
B	2.16624	-0.30613	-1.48792
B	1.94171	-1.40562	1.1781
C	-1.42947	-1.18565	2.23585
H	-0.66522	-1.93408	2.41588
C	-2.6303	-1.18307	2.94043
C	-3.60512	-0.21517	2.66508
C	-3.27011	0.7372	1.6961
C	-2.04213	0.68941	1.02864
C	-1.65929	1.66388	-0.01676
C	-2.38633	2.83405	-0.25621
C	-2.02346	3.72855	-1.26759
C	-0.88667	3.38282	-2.00789
C	-0.17841	2.22368	-1.71461
H	0.7268	1.95772	-2.24962
C	3.12205	2.87517	1.54526
C	2.01647	2.72112	2.6546
C	4.14505	-0.78765	-2.59394
C	3.42863	0.41839	-3.31373
C	2.49328	-2.78154	2.96272
C	3.79707	-2.3008	2.22524
Ir	0.65481	-0.37533	-0.10734
N	-1.13984	-0.28792	1.28396
N	-0.54502	1.36659	-0.74174
O	2.5828	2.07344	0.46353
O	1.42133	1.44156	2.32312
O	3.09712	-1.31388	-1.74992
O	2.39451	0.76558	-2.36366
O	1.46594	-2.43395	2.0015
O	3.29855	-1.21665	1.40219
H	0.76718	-1.98982	-0.3203
C	-2.28944	-1.0109	-2.24225
C	3.33878	4.29823	1.03131
H	3.70234	4.95612	1.82985
H	4.09027	4.28879	0.23488
H	2.4209	4.72332	0.61801
C	4.46893	2.24788	1.93461
H	5.12772	2.25125	1.05999
H	4.96397	2.80709	2.73734
H	4.3378	1.20765	2.24578
C	2.5349	2.66051	4.09142
H	3.04135	3.59223	4.36952
H	1.69633	2.5136	4.77887
H	3.23385	1.83109	4.23175
C	0.9	3.77256	2.55089
H	0.08115	3.48505	3.21866
H	1.24652	4.76998	2.84087
H	0.5023	3.82301	1.53246
C	4.89656	-1.76038	3.13884
H	5.73759	-1.408	2.53188
H	4.54396	-0.9236	3.74596
H	5.26977	-2.54503	3.80825
C	4.3821	-3.35695	1.2744
H	5.16386	-2.88884	0.66891
H	4.82275	-4.19655	1.82103
H	3.61807	-3.73846	0.59096
C	2.19691	-1.99236	4.24662
H	1.19565	-2.25766	4.60449
H	2.91367	-2.22594	5.04254
H	2.21267	-0.91491	4.05959
C	2.41422	-4.2826	3.23625
H	3.21397	-4.60223	3.91419
H	1.45591	-4.52094	3.71121
H	2.48492	-4.86483	2.3144
C	2.73907	0.02605	-4.62801
H	2.10413	0.85507	-4.9593
H	3.46419	-0.18716	-5.42049

H	2.10401	-0.85468	-4.49415
C	4.30335	1.65277	-3.54331
H	5.14855	1.42205	-4.20165
H	3.70964	2.4399	-4.02165
H	4.68806	2.04959	-2.6022
C	4.63126	-1.90238	-3.52055
H	5.40122	-1.53699	-4.21106
H	5.07024	-2.70755	-2.92196
H	3.81277	-2.32918	-4.1055
C	5.28859	-0.34394	-1.66812
H	5.61943	-1.20508	-1.07963
H	6.14633	0.04002	-2.23335
H	4.94997	0.42442	-0.96724
Si	-0.76664	-2.00779	-1.54297
C	0.10519	-2.74708	-3.06872
H	0.3456	-1.9698	-3.80304
H	1.04755	-3.22695	-2.78206
H	-0.52795	-3.49385	-3.56361
C	-1.48623	-3.49074	-0.57828
H	-2.14942	-3.1698	0.23228
H	-2.06752	-4.14241	-1.24294
H	-0.68187	-4.08891	-0.13263
H	-0.52137	4.00885	-2.81344
H	-2.79201	-1.95435	3.68219
C	-4.98406	-0.18369	3.33887
H	-3.24187	3.05299	0.36926
H	-3.98738	1.50224	1.43132
C	-2.84308	5.00582	-1.51057
C	-2.28191	5.83936	-2.67823
H	-2.89404	6.73741	-2.81577
H	-1.25348	6.16862	-2.48907
H	-2.29539	5.28194	-3.62165
C	-4.30424	4.62199	-1.84452
H	-4.35171	4.00035	-2.74668
H	-4.77715	4.064	-1.02937
H	-4.90005	5.52459	-2.02006
C	-2.81903	5.87881	-0.23181
H	-1.79485	6.17028	0.02685
H	-3.40414	6.79131	-0.38946
H	-3.24555	5.35371	0.62951
C	-6.07831	-0.26235	2.24732
H	-7.07112	-0.31707	2.71129
H	-6.06829	0.61763	1.59532
H	-5.94041	-1.14343	1.61208
C	-5.13654	1.13518	4.13346
H	-6.12686	1.1822	4.60201
H	-4.38211	1.20885	4.92508
H	-5.03136	2.01394	3.48793
C	-5.18012	-1.36354	4.30954
H	-5.1055	-2.32881	3.79623
H	-4.44684	-1.34874	5.12391
H	-6.17565	-1.30547	4.76301
H	-2.62677	-0.30514	-1.47972
H	-1.91677	-0.41656	-3.0858
C	-3.44469	-1.86317	-2.67974
C	-3.57203	-2.3734	-3.98183
C	-4.46451	-2.21307	-1.78586
C	-4.65059	-3.17764	-4.35726
H	-2.80722	-2.12133	-4.713
C	-5.55005	-3.0075	-2.12643
C	-5.64403	-3.49663	-3.42991
H	-4.71471	-3.55111	-5.37616
H	-6.30047	-3.2298	-1.37412
H	-6.48555	-4.12066	-3.71421
F	-4.38868	-1.744	-0.50639

Thermal correction to Energy= 1.197293

Thermal correction to Enthalpy= 1.198238

Thermal correction to Gibbs Free Energy= 1.026961

Sum of electronic and zero-point Energies= -2887.487864

Sum of electronic and thermal Energies= -2887.420405

Sum of electronic and thermal Enthalpies= -2887.419460

Sum of electronic and thermal Free Energies= -2887.590737SCF Done: E(wB97XD) = -2888.634733

TS4a′

Number of Negative Frequencies = 1

B	0.99652	1.68487	-1.4572
B	-1.20248	1.93369	0.26075
B	-1.90713	-0.45649	-1.3519
C	1.02898	-2.34237	-1.88029
H	0.06998	-2.27953	-2.37804
C	1.97645	-3.29496	-2.23693
C	3.18991	-3.3745	-1.54199
C	3.35504	-2.4642	-0.49355
C	2.36436	-1.52731	-0.17717
C	2.5016	-0.5527	0.92303
C	3.59013	-0.56368	1.80069
C	3.72805	0.38476	2.81794

C	2.71211	1.34395	2.89332
C	1.63847	1.30049	2.01327
H	0.84903	2.03927	2.04671
C	1.6527	3.34949	-2.92111
C	2.84134	2.97678	-1.96248
C	-2.7668	3.62991	0.41405
C	-1.54153	3.98352	1.33219
C	-3.03642	-1.87213	-2.82089
C	-3.9225	-0.59694	-2.50836
Ir	-0.16702	0.25832	-0.34785
N	1.20988	-1.46317	-0.8835
N	1.50864	0.36943	1.05242
O	0.52575	2.70892	-2.27098
O	2.38385	1.72157	-1.38962
O	-2.55233	2.22834	0.14875
O	-0.5531	3.02228	0.88827
O	-1.96206	-1.76219	-1.8511
O	-3.01984	0.2495	-1.76327
H	-0.57839	0.60808	-1.86834
C	-1.21471	-2.97767	1.21366
C	1.35897	4.84417	-3.04616
H	2.21355	5.37954	-3.47713
H	0.49803	4.99569	-3.7057
H	1.12273	5.28807	-2.07634
C	1.77768	2.7213	-4.31744
H	0.82738	2.84465	-4.84647
H	2.56692	3.19663	-4.9107
H	1.99001	1.64963	-4.2495
C	4.18546	2.73728	-2.64851
H	4.5393	3.64674	-3.14872
H	4.93377	2.45075	-1.90176
H	4.12412	1.93511	-3.38789
C	3.00939	3.96064	-0.79534
H	3.71482	3.53595	-0.07332
H	3.40168	4.92747	-1.1303
H	2.05742	4.12277	-0.28077
C	-4.38056	0.191	-3.74103
H	-4.93687	1.07507	-3.41345
H	-3.53641	0.53488	-4.34349
H	-5.0435	-0.41073	-4.374
C	-5.14388	-0.8819	-1.62354
H	-5.57345	0.07325	-1.30514
H	-5.91268	-1.43703	-2.17354
H	-4.88453	-1.45079	-0.72963
C	-2.38368	-1.84248	-4.21195
H	-1.69043	-2.68609	-4.30218
H	-3.12658	-1.93078	-5.01205
H	-1.81617	-0.9196	-4.36858
C	-3.73339	-3.21812	-2.60943
H	-4.58821	-3.32886	-3.28707
H	-3.03023	-4.03153	-2.81971
H	-4.08238	-3.33376	-1.58151
C	-1.80215	3.7241	2.82418
H	-0.85876	3.81273	3.37453
H	-2.50859	4.44723	3.2466
H	-2.19791	2.7172	2.98926
C	-0.9699	5.38945	1.1461
H	-1.71062	6.1542	1.40903
H	-0.1004	5.52618	1.79888
H	-0.64895	5.55592	0.11524
C	-4.14021	3.79962	1.06429
H	-4.32209	4.84663	1.33586
H	-4.91781	3.49348	0.35709
H	-4.24356	3.18269	1.9602
C	-2.73781	4.35078	-0.94371
H	-3.4989	3.90203	-1.58951
H	-2.95258	5.42137	-0.84548
H	-1.76881	4.22313	-1.4355
Si	-1.31372	-1.09043	1.50102
C	-0.50927	-0.86001	3.23047
H	0.52477	-1.22433	3.25554
H	-0.49886	0.1869	3.558
H	-1.07557	-1.43393	3.97543
C	-3.17067	-0.61252	1.83662
H	-3.68789	-0.52295	0.87899
H	-3.14914	0.40041	2.25525
H	2.72978	2.13111	3.63714
H	1.74856	-3.96489	-3.05704
C	4.29676	-4.38516	-1.88036
H	4.34402	-1.33251	1.6886
H	4.27463	-2.47691	0.07773
C	4.93093	0.34242	3.77193
C	4.88688	1.4831	4.80643
H	5.76048	1.41721	5.46426
H	4.90794	2.46887	4.32792
H	3.99323	1.42678	5.43821
C	4.93098	-1.00614	4.53122
H	4.01388	-1.12744	5.11833

H	5.00671	-1.85977	3.84924
H	5.78418	-1.05192	5.21844
C	6.23901	0.4751	2.95577
H	6.26819	1.425	2.41021
H	7.10632	0.44068	3.62568
H	6.35091	-0.33396	2.22604
C	4.56476	-5.28373	-0.64917
H	5.35678	-6.00711	-0.87636
H	4.88634	-4.70135	0.22091
H	3.66551	-5.84176	-0.36523
C	5.58971	-3.61981	-2.25143
H	6.38995	-4.32836	-2.49604
H	5.42874	-2.97406	-3.12198
H	5.94338	-2.98979	-1.42826
C	3.91101	-5.29	-3.06597
H	3.01263	-5.88066	-2.85378
H	3.73473	-4.7118	-3.98008
H	4.72577	-5.99272	-3.27245
H	-0.16431	-3.29595	1.1926
H	-1.71147	-3.53453	2.01792
H	-1.66843	-3.25994	0.25989
C	-3.90996	-1.52726	2.76793
C	-4.51468	-2.70631	2.31664
C	-4.02835	-1.27844	4.14635
C	-5.19698	-3.593	3.13742
C	-4.7021	-2.15052	5.00407
H	-3.58293	-0.36999	4.54486
C	-5.29021	-3.31269	4.50243
H	-5.64096	-4.48219	2.70083
H	-4.77164	-1.91736	6.06329
H	-5.82008	-3.9953	5.16081
F	-4.43967	-3.00457	0.9876

Thermal correction to Energy= 1.195956

Thermal correction to Enthalpy= 1.196900

Thermal correction to Gibbs Free Energy= 1.027596

Sum of electronic and zero-point Energies= -2887.478415

Sum of electronic and thermal Energies= -2887.411711

Sum of electronic and thermal Enthalpies= -2887.410767

Sum of electronic and thermal Free Energies= -2887.580071

SCF Done: E(wB97XD) = -2888.623755

Int5a′

Number of Negative Frequencies = 0

B	0.96291	1.6883	-1.48075
B	-1.21382	1.90415	0.27115
B	-1.87803	-0.41805	-1.39529
C	1.01191	-2.39363	-1.81754
H	0.04498	-2.33814	-2.30141
C	1.9517	-3.35805	-2.16534
C	3.17551	-3.4243	-1.48772
C	3.35947	-2.48879	-0.4631
C	2.37683	-1.54187	-0.15328
C	2.53044	-0.54634	0.92643
C	3.62216	-0.55124	1.7999
C	3.76725	0.41277	2.80302
C	2.75616	1.37778	2.86823
C	1.68173	1.32717	1.99142
H	0.89408	2.07019	2.01326
C	1.60656	3.3804	-2.91262
C	2.80247	2.99102	-1.97038
C	-2.79528	3.58179	0.4498
C	-1.55412	3.95995	1.33536
C	-3.03198	-1.82634	-2.85015
C	-3.87164	-0.51434	-2.58561
Ir	-0.16247	0.24045	-0.34166
N	1.21208	-1.49079	-0.84548
N	1.54686	0.38449	1.04498
O	0.48571	2.73024	-2.26317
O	2.35033	1.71976	-1.42413
O	-2.56983	2.17885	0.19526
O	-0.56412	3.00932	0.87379
O	-1.98867	-1.73983	-1.84533
O	-2.93159	0.32939	-1.88361
H	-0.36996	0.61861	-1.91231
C	-1.21572	-2.97478	1.20897
C	1.31146	4.87657	-3.01327
H	2.16257	5.41786	-3.44242
H	0.44559	5.03598	-3.66313
H	1.083	5.30521	-2.03514
C	1.72077	2.77136	-4.31877
H	0.76653	2.89948	-4.83743
H	2.5048	3.25694	-4.9121
H	1.93723	1.70016	-4.26812
C	4.14259	2.77024	-2.66917
H	4.48912	3.69147	-3.15312
H	4.89718	2.47433	-1.93281
H	4.08096	1.98232	-3.42354
C	2.97295	3.95038	-0.78368

H	3.68179	3.51292	-0.07299
H	3.36307	4.92438	-1.10233
H	2.02333	4.10264	-0.26245
C	-4.31986	0.23508	-3.84386
H	-4.85264	1.14621	-3.54994
H	-3.4722	0.53251	-4.46627
H	-5.00396	-0.37541	-4.44716
C	-5.08823	-0.72642	-1.67222
H	-5.47736	0.25175	-1.37628
H	-5.88437	-1.27268	-2.19067
H	-4.83176	-1.27718	-0.76534
C	-2.33594	-1.84015	-4.21853
H	-1.66228	-2.70247	-4.27151
H	-3.0543	-1.9233	-5.0415
H	-1.74109	-0.934	-4.37333
C	-3.78983	-3.13953	-2.64457
H	-4.6329	-3.2208	-3.34104
H	-3.11805	-3.98471	-2.83089
H	-4.1664	-3.23016	-1.62417
C	-1.77571	3.70717	2.8345
H	-0.82144	3.80999	3.36137
H	-2.48033	4.42393	3.26974
H	-2.15595	2.69636	3.01499
C	-1.0092	5.37197	1.12682
H	-1.75611	6.12825	1.39826
H	-0.12897	5.52893	1.76248
H	-0.70972	5.53484	0.08891
C	-4.15665	3.74183	1.12902
H	-4.34468	4.79059	1.39426
H	-4.94511	3.42064	0.4414
H	-4.23335	3.1342	2.0329
C	-2.80383	4.28732	-0.91611
H	-3.56906	3.81706	-1.54304
H	-3.03553	5.3551	-0.82582
H	-1.84296	4.169	-1.42323
Si	-1.28242	-1.08915	1.52013
C	-0.46015	-0.8882	3.24573
H	0.57218	-1.25866	3.2534
H	-0.43883	0.15503	3.58468
H	-1.02069	-1.46701	3.98996
C	-3.1342	-0.60037	1.87154
H	-3.64648	-0.47071	0.91673
H	-3.10335	0.39552	2.32811
H	2.78144	2.175	3.60258
H	1.70989	-4.04739	-2.96514
C	4.27266	-4.44661	-1.81762
H	4.37121	-1.3261	1.69916
H	4.2873	-2.49104	0.09442
C	4.97255	0.37825	3.75258
C	4.93558	1.53213	4.77265
H	5.81163	1.47226	5.42779
H	4.9588	2.51172	4.28164
H	4.04435	1.48765	5.40868
C	4.97256	-0.9603	4.52941
H	4.05734	-1.07056	5.11981
H	5.04426	-1.82297	3.85849
H	5.82812	-0.99874	5.21414
C	6.27749	0.49858	2.92934
H	6.30727	1.44183	2.37237
H	7.14763	0.46939	3.5943
H	6.38357	-0.31946	2.20879
C	4.56527	-5.30877	-0.56612
H	5.34995	-6.04142	-0.78899
H	4.90958	-4.70198	0.27885
H	3.67097	-5.85491	-0.24584
C	5.55814	-3.69411	-2.23737
H	6.35342	-4.41023	-2.47609
H	5.38016	-3.07487	-3.12375
H	5.9292	-3.03945	-1.4408
C	3.86202	-5.38604	-2.96742
H	2.96692	-5.96786	-2.7198
H	3.66877	-4.83566	-3.89515
H	4.67147	-6.09644	-3.1684
H	-0.17148	-3.31301	1.1764
H	-1.71777	-3.52953	2.01136
H	-1.68303	-3.23661	0.25622
C	-3.88848	-1.54121	2.76647
C	-4.51907	-2.68509	2.26642
C	-3.99601	-1.35085	4.15498
C	-5.21451	-3.59405	3.04945
C	-4.68427	-2.24695	4.97691
H	-3.53166	-0.46941	4.59082
C	-5.2972	-3.37271	4.42609
H	-5.67975	-4.45407	2.57648
H	-4.74469	-2.05955	6.04574
H	-5.83722	-4.07347	5.05657
F	-4.45671	-2.92362	0.92494

Thermal correction to Energy= 1.198863

Thermal correction to Enthalpy= 1.199807

Thermal correction to Gibbs Free Energy= 1.023427

Sum of electronic and zero-point Energies= -2887.496458

Sum of electronic and thermal Energies= -2887.428373

Sum of electronic and thermal Enthalpies= -2887.427429

Sum of electronic and thermal Free Energies= -2887.603809

SCF Done: E(wB97XD) = -2888.63228

Int6a′

Number of Negative Frequencies = 0

B	-2.1663	-1.26499	0.02643
B	-0.37781	-1.11606	1.85283
C	1.49987	1.93298	1.77344
H	0.60834	2.24827	2.29651
C	2.74431	2.44141	2.12888
C	3.89104	2.01059	1.45436
C	3.68518	1.0657	0.44302
C	2.40716	0.59806	0.12273
C	2.17551	-0.41078	-0.93891
C	3.19234	-0.85555	-1.7896
C	2.95174	-1.82782	-2.76821
C	1.64581	-2.32522	-2.82829
C	0.6722	-1.83407	-1.96194
H	-0.34118	-2.21544	-1.97375
C	-4.17323	-2.42071	0.09763
C	-3.12796	-3.22865	-0.76104
C	-0.56456	-2.34966	3.80849
C	0.82668	-2.6247	3.13277
Ir	-0.68229	0.18897	0.19764
N	1.3139	1.0384	0.79435
N	0.91496	-0.88974	-1.04441
O	-3.53035	-1.13318	0.22827
O	-1.8835	-2.55215	-0.45046
O	-1.28809	-1.67027	2.74955
O	0.91387	-1.54523	2.17196
C	-0.73543	1.82373	-1.32683
C	-0.34005	1.66475	-2.66521
C	-0.21206	2.76118	-3.52377
H	0.09985	2.61668	-4.55548
C	-0.49616	4.04445	-3.05764
H	-0.41017	4.9241	-3.68803
C	-0.91113	4.18396	-1.73897
C	-1.05765	3.11491	-0.85057
C	5.3051	2.51669	1.77511
C	4.08072	-2.29063	-3.70181
C	-5.53573	-2.21272	-0.56722
H	-6.04188	-3.17058	-0.73847
H	-6.1711	-1.60738	0.08724
H	-5.44627	-1.68865	-1.52177
C	-4.36782	-2.97818	1.51497
H	-4.98077	-2.27453	2.08741
H	-4.87807	-3.94813	1.50466
H	-3.41028	-3.07661	2.03117
C	-2.9816	-4.70493	-0.3909
H	-3.91879	-5.24851	-0.55991
H	-2.20687	-5.16746	-1.0123
H	-2.69321	-4.82838	0.65552
C	-3.35201	-3.09349	-2.27537
H	-2.50199	-3.53653	-2.80617
H	-4.26009	-3.61264	-2.60054
H	-3.42868	-2.04284	-2.57274
C	2.03464	-2.55005	4.06683
H	2.95276	-2.71704	3.49311
H	2.11254	-1.57239	4.54843
H	1.97982	-3.31998	4.84586
C	0.86287	-3.93662	2.33276
H	1.78405	-3.96446	1.74134
H	0.84796	-4.81645	2.98597
H	0.0178	-3.99434	1.63978
C	-0.48282	-1.3789	4.99591
H	-1.49607	-1.07973	5.2816
H	-0.00234	-1.84048	5.86593
H	0.07034	-0.47324	4.72978
C	-1.34602	-3.59785	4.2202
H	-0.80359	-4.16474	4.98664
H	-2.31391	-3.30496	4.64037
H	-1.53317	-4.25801	3.36992
H	-0.1249	0.67133	-3.05021
H	4.18397	-0.43009	-1.70071
H	4.53708	0.67783	-0.09818
H	1.36176	-3.08861	-3.54254
H	2.79215	3.16553	2.93278
C	6.19837	1.32071	2.18289
H	6.27909	0.57642	1.38348
H	7.21106	1.67094	2.41442
H	5.80081	0.81711	3.07107
C	5.30418	3.53851	2.92802
H	6.32856	3.87476	3.12173

H	4.70636	4.4249	2.68783
H	4.91851	3.10455	3.85755
C	5.8957	3.19886	0.51745
H	5.2807	4.05008	0.20479
H	6.90606	3.56822	0.72901
H	5.96496	2.50702	-0.3288
C	3.60453	-3.3668	-4.69568
H	2.80288	-2.99727	-5.34513
H	4.43837	-3.66646	-5.3398
H	3.24479	-4.26576	-4.18227
C	5.23333	-2.8844	-2.85683
H	5.64944	-2.15125	-2.15759
H	4.89068	-3.74684	-2.27404
H	6.0474	-3.21821	-3.51097
C	4.60224	-1.07766	-4.50899
H	3.80506	-0.63936	-5.11981
H	4.99637	-0.28953	-3.85855
H	5.41101	-1.39133	-5.17957
F	-1.17754	5.4395	-1.29019
H	-1.83497	0.4538	-0.90647
C	-1.5029	3.35675	0.57594
H	-0.66477	3.77764	1.15221
Si	-2.10719	1.70161	1.37671
H	-2.28212	4.13136	0.60676
C	-1.94761	1.83803	3.2798
H	-2.5243	2.69709	3.64742
H	-2.36333	0.93328	3.73751
H	-0.92312	1.94372	3.65704
C	-3.98932	1.69327	1.08659
H	-4.23325	1.64779	0.01959
H	-4.47457	0.83932	1.56571
H	-4.41877	2.61774	1.49623

Thermal correction to Energy= 0.991660

Thermal correction to Enthalpy= 0.992604

Thermal correction to Gibbs Free Energy= 0.846101

Sum of electronic and zero-point Energies= -2475.786232

Sum of electronic and thermal Energies= -2475.730331

Sum of electronic and thermal Enthalpies= -2475.729387

Sum of electronic and thermal Free Energies= -2475.874917

SCF Done: E(wB97XD) = -2475.5532

TS7a′

Number of Negative Frequencies = 1

B	-2.19875	-1.1184	-0.09407
B	-0.31483	-1.15368	1.69653
C	1.47082	2.19964	1.60293
H	0.58231	2.56402	2.09716
C	2.70711	2.77948	1.86672
C	3.85022	2.31158	1.21179
C	3.64876	1.25681	0.31481
C	2.38273	0.70472	0.09905
C	2.15796	-0.39604	-0.86726
C	3.20925	-1.07132	-1.49507
C	2.96976	-2.07876	-2.43724
C	1.62581	-2.35719	-2.70603
C	0.62328	-1.66809	-2.0291
H	-0.4201	-1.90981	-2.17836
C	-4.21739	-2.25514	-0.11924
C	-3.14935	-3.06713	-0.94169
C	-0.42173	-2.5659	3.53728
C	0.91849	-2.80944	2.75223
Ir	-0.71096	0.30631	0.20096
N	1.29192	1.18523	0.74616
N	0.86862	-0.71274	-1.12413
O	-3.57207	-0.97035	0.02677
O	-1.9117	-2.40667	-0.57318
O	-1.18442	-1.76413	2.59971
O	0.9779	-1.65026	1.88555
C	-0.89153	2.07353	-1.14923
C	-0.43933	2.07111	-2.47918
C	-0.3634	3.25018	-3.22343
H	-0.00753	3.22888	-4.25107
C	-0.75464	4.46066	-2.64995
H	-0.71589	5.40019	-3.19228
C	-1.21615	4.44397	-1.34028
C	-1.31219	3.28788	-0.55951
C	5.25306	2.89891	1.425
C	4.13955	-2.81866	-3.10358
C	-5.55878	-2.04475	-0.82344
H	-6.06211	-3.00175	-1.00716
H	-6.21119	-1.43636	-0.18893
H	-5.4397	-1.52347	-1.77633
C	-4.45162	-2.8078	1.29478
H	-5.08275	-2.1037	1.84641
H	-4.95932	-3.77888	1.27435
H	-3.50811	-2.90384	1.83842
C	-3.03387	-4.54786	-0.57879
H	-3.9717	-5.07813	-0.78281

H	-2.24489	-5.01586	-1.17779
H	-2.78173	-4.68202	0.47566
C	-3.31179	-2.91615	-2.46227
H	-2.44358	-3.35924	-2.9628
H	-4.20867	-3.4274	-2.82859
H	-3.37201	-1.8622	-2.75206
C	2.18198	-2.84375	3.61245
H	3.05933	-2.98312	2.97146
H	2.31645	-1.91281	4.16859
H	2.1511	-3.67539	4.32673
C	0.87191	-4.04454	1.83901
H	1.75754	-4.04047	1.19466
H	0.86933	-4.97878	2.41197
H	-0.01159	-4.01999	1.19393
C	-0.23527	-1.72548	4.80922
H	-1.21847	-1.43567	5.19297
H	0.28815	-2.28534	5.59255
H	0.32503	-0.80981	4.59891
C	-1.22098	-3.82702	3.86773
H	-0.65626	-4.48642	4.53782
H	-2.15177	-3.54973	4.37374
H	-1.48104	-4.38827	2.96704
H	-0.14488	1.13295	-2.94208
H	4.23048	-0.82211	-1.23609
H	4.4923	0.86899	-0.24137
H	1.33518	-3.11617	-3.42223
H	2.74977	3.59657	2.57635
C	6.19701	1.78817	1.94499
H	6.27825	0.95491	1.23875
H	7.20419	2.1928	2.0992
H	5.84271	1.38449	2.9001
C	5.24658	4.05087	2.44755
H	6.26383	4.43782	2.57159
H	4.61527	4.88424	2.11929
H	4.89684	3.72085	3.43231
C	5.78817	3.44359	0.07858
H	5.13628	4.23146	-0.31467
H	6.78971	3.86753	0.21693
H	5.86151	2.65875	-0.6818
C	3.65451	-3.87674	-4.11257
H	3.07073	-3.4291	-4.92502
H	4.51766	-4.37741	-4.56442
H	3.04085	-4.64757	-3.63272
C	4.97634	-3.53089	-2.01376
H	5.38837	-2.8244	-1.28519
H	4.3694	-4.26054	-1.46608
H	5.8174	-4.06373	-2.47295
C	5.03047	-1.801	-3.85568
H	4.46306	-1.28069	-4.63552
H	5.44705	-1.04384	-3.18282
H	5.87071	-2.31762	-4.33428
F	-1.59137	5.62778	-0.78575
H	-1.85887	0.75278	-0.86755
C	-1.81976	3.37153	0.86576
H	-1.10697	3.95418	1.47073
Si	-2.09384	1.61284	1.63908
H	-2.74728	3.9605	0.89939
C	-1.59989	1.68961	3.48999
H	-2.07449	2.55134	3.9792
H	-1.96157	0.7837	3.98907
H	-0.52243	1.74837	3.6807
C	-3.98567	1.41719	1.72319
H	-4.44251	1.41956	0.72939
H	-4.28188	0.4866	2.21654
H	-4.39982	2.25769	2.29841

Thermal correction to Energy= 0.989611

Thermal correction to Enthalpy= 0.990555

Thermal correction to Gibbs Free Energy= 0.843356

Sum of electronic and zero-point Energies= -2475.773720

Sum of electronic and thermal Energies= -2475.717860

Sum of electronic and thermal Enthalpies= -2475.716916

Sum of electronic and thermal Free Energies= -2475.864114

SCF Done: E(wB97XD) = -2476.669873

Int8a′

Number of Negative Frequencies = 0

Ir	-0.73235	0.31249	0.14963
C	1.54687	2.32165	1.45988
C	2.64911	3.16862	1.35504
C	3.45769	1.75307	-0.37405
C	2.34082	0.93246	-0.20077
C	2.1622	-0.34412	-0.94244
C	3.21943	-0.97739	-1.59992
C	1.76173	-2.77111	-2.14972
C	0.74807	-2.08975	-1.47905
N	1.37117	1.23974	0.69399
N	0.92968	-0.89947	-0.89476
O	-3.67179	-0.54819	-0.67686

O	-2.01681	-1.00663	-2.17095
C	3.63666	2.90965	0.39894
C	3.04191	-2.21472	-2.23514
C	-4.34599	-0.78044	-1.93788
C	-3.22563	-1.47294	-2.80543
H	-0.25517	-2.49055	-1.41963
H	0.76558	2.51568	2.18412
B	-2.29317	-0.47857	-0.89657
C	4.85594	3.81587	0.17302
C	4.2129	-2.8889	-2.96633
C	-5.59506	-1.6265	-1.68784
H	-6.27752	-1.08381	-1.02548
H	-6.12495	-1.82766	-2.6267
H	-5.3572	-2.58341	-1.21714
C	-4.76422	0.59616	-2.48044
H	-5.41983	1.08027	-1.75029
H	-3.89455	1.24293	-2.63027
H	-5.30846	0.51283	-3.42777
C	-3.22763	-3.00658	-2.72235
H	-4.10198	-3.44344	-3.21796
H	-2.33045	-3.38838	-3.22228
H	-3.20518	-3.3483	-1.68493
C	-3.19152	-1.04579	-4.27618
H	-2.37084	-1.56117	-4.78739
H	-4.12486	-1.30923	-4.78843
H	-3.0249	0.02867	-4.37706
C	-0.76942	1.8077	-1.2344
C	-1.12848	3.12954	-0.86071
C	-0.36814	1.56682	-2.56405
C	-1.05678	4.12472	-1.83985
C	-0.31538	2.59647	-3.50701
H	-0.12975	0.5571	-2.87446
C	-0.66265	3.9	-3.15176
H	-0.00829	2.37833	-4.52774
H	-0.63593	4.725	-3.85665
F	-1.39319	5.39645	-1.48781
H	2.70367	4.02859	2.01134
H	1.52891	-3.72987	-2.5971
C	3.81139	-4.24589	-3.57457
H	3.00529	-4.14136	-4.30941
H	4.67181	-4.68662	-4.08993
H	3.48631	-4.95696	-2.80659
C	5.3742	-3.12533	-1.97135
H	5.73896	-2.18896	-1.53602

H	5.06103	-3.77897	-1.14935
H	6.21735	-3.60358	-2.48364
C	4.69449	-1.96381	-4.1101
H	3.89352	-1.78493	-4.83613
H	5.02915	-0.99073	-3.73526
H	5.53668	-2.42559	-4.63887
C	4.81676	4.3607	-1.27529
H	5.6826	5.00867	-1.45608
H	4.84192	3.55508	-2.01656
H	3.90835	4.94724	-1.45037
C	4.87586	5.01227	1.14289
H	4.92363	4.68973	2.18937
H	5.76036	5.62794	0.94686
H	3.99498	5.65169	1.01901
C	6.1523	2.99711	0.38316
H	6.20556	2.59638	1.402
H	6.22495	2.15496	-0.31341
H	7.02936	3.6352	0.22354
C	-1.5867	3.45266	0.54197
H	-0.76205	3.90229	1.11614
H	-2.37717	4.21366	0.52455
Si	-2.149	1.79411	1.35518
C	-4.05422	1.83503	1.22718
H	-4.52845	0.9117	1.57075
H	-4.43133	2.66315	1.84428
H	-4.38019	2.00609	0.1969
C	-1.83511	1.90228	3.24429
H	-2.31538	1.0581	3.75187
H	-0.77651	1.86448	3.52078
H	-2.26792	2.82953	3.6459
H	4.18531	1.50075	-1.13594
H	4.19795	-0.51228	-1.59734

Thermal correction to Energy= 0.789421

Thermal correction to Enthalpy= 0.790365

Thermal correction to Gibbs Free Energy= 0.665814

Sum of electronic and zero-point Energies= -2064.101962

Sum of electronic and thermal Energies= -2064.056902

Sum of electronic and thermal Enthalpies= -2064.055958

Sum of electronic and thermal Free Energies= -2064.180509

SCF Done: E(wB97XD) = -2064.74881

TS9a′

Number of Negative Frequencies = 0

Ir	-0.73235	0.31249	0.14963

C	1.54687	2.32165	1.45988	H	-2.37084	-1.56117	-4.78739
C	2.64911	3.16862	1.35504	H	-4.12486	-1.30923	-4.78843
C	3.45769	1.75307	-0.37405	H	-3.0249	0.02867	-4.37706
C	2.34082	0.93246	-0.20077	C	-3.59037	-4.27461	1.02062
C	2.1622	-0.34412	-0.94244	H	-3.05024	-4.9479	0.34674
C	3.21943	-0.97739	-1.59992	H	-4.3837	-4.85187	1.50996
C	1.76173	-2.77111	-2.14972	H	-4.04997	-3.48505	0.42548
C	0.74807	-2.08975	-1.47905	C	-4.47474	-1.902	2.45871
B	0.32662	-1.31807	2.53498	H	-5.34396	-2.56429	2.37189
N	1.37117	1.23974	0.69399	H	-4.74004	-1.08124	3.13249
N	0.92968	-0.89947	-0.89476	H	-4.25174	-1.47713	1.4768
O	1.32338	-2.25307	2.36114	C	-3.61645	-3.19344	4.42927
O	0.65736	-0.37395	3.47753	H	-4.01301	-2.38319	5.04924
O	-3.67179	-0.54819	-0.67686	H	-4.38348	-3.97386	4.36224
O	-2.01681	-1.00663	-2.17095	H	-2.74142	-3.60742	4.93617
O	-2.19383	-1.67701	3.20402	C	-1.87963	-4.83899	2.75958
O	-1.60372	-2.91264	1.37001	H	-1.32097	-5.4104	2.01179
C	3.63666	2.90965	0.39894	H	-1.16039	-4.45628	3.49047
C	3.04191	-2.21472	-2.23514	H	-2.5686	-5.5198	3.27092
C	1.81235	-0.85659	4.21893	C	2.89264	-3.23217	3.90608
C	2.44031	-1.93088	3.23854	H	3.26215	-3.92316	3.14134
C	-2.62855	-3.69817	2.05405	H	3.70585	-3.04762	4.61749
C	-3.27102	-2.64804	3.04285	H	2.0728	-3.7244	4.43366
C	-4.34599	-0.78044	-1.93788	C	1.25686	-1.45507	5.52031
C	-3.22563	-1.47294	-2.80543	H	2.05665	-1.81967	6.1739
B	-1.2896	-1.84877	2.18546	H	0.69915	-0.67973	6.05401
H	-0.25517	-2.49055	-1.41963	H	0.56516	-2.27856	5.31858
H	0.76558	2.51568	2.18412	C	2.7163	0.33121	4.54932
B	-2.29317	-0.47857	-0.89657	H	2.17381	1.03133	5.19271
C	4.85594	3.81587	0.17302	H	3.61196	0.00126	5.08854
C	4.2129	-2.8889	-2.96633	H	3.02793	0.87058	3.65266
C	-5.59506	-1.6265	-1.68784	C	3.5766	-1.39059	2.36352
H	-6.27752	-1.08381	-1.02548	H	4.48053	-1.20859	2.95533
H	-6.12495	-1.82766	-2.6267	H	3.8137	-2.13051	1.59264
H	-5.3572	-2.58341	-1.21714	H	3.2953	-0.4613	1.86597
C	-4.76422	0.59616	-2.48044	C	-0.76942	1.8077	-1.2344
H	-5.41983	1.08027	-1.75029	C	-1.12848	3.12954	-0.86071
H	-3.89455	1.24293	-2.63027	C	-0.36814	1.56682	-2.56405
H	-5.30846	0.51283	-3.42777	C	-1.05678	4.12472	-1.83985
C	-3.22763	-3.00658	-2.72235	C	-0.31538	2.59647	-3.50701
H	-4.10198	-3.44344	-3.21796	H	-0.12975	0.5571	-2.87446
H	-2.33045	-3.38838	-3.22228	C	-0.66265	3.9	-3.15176
H	-3.20518	-3.3483	-1.68493	H	-0.00829	2.37833	-4.52774
C	-3.19152	-1.04579	-4.27618	H	-0.63593	4.725	-3.85665

F	-1.39319	5.39645	-1.48781
H	2.70367	4.02859	2.01134
H	1.52891	-3.72987	-2.5971
C	3.81139	-4.24589	-3.57457
H	3.00529	-4.14136	-4.30941
H	4.67181	-4.68662	-4.08993
H	3.48631	-4.95696	-2.80659
C	5.3742	-3.12533	-1.97135
H	5.73896	-2.18896	-1.53602
H	5.06103	-3.77897	-1.14935
H	6.21735	-3.60358	-2.48364
C	4.69449	-1.96381	-4.1101
H	3.89352	-1.78493	-4.83613
H	5.02915	-0.99073	-3.73526
H	5.53668	-2.42559	-4.63887
C	4.81676	4.3607	-1.27529
H	5.6826	5.00867	-1.45608
H	4.84192	3.55508	-2.01656
H	3.90835	4.94724	-1.45037
C	4.87586	5.01227	1.14289
H	4.92363	4.68973	2.18937
H	5.76036	5.62794	0.94686
H	3.99498	5.65169	1.01901
C	6.1523	2.99711	0.38316
H	6.20556	2.59638	1.402
H	6.22495	2.15496	-0.31341
H	7.02936	3.6352	0.22354
C	-1.5867	3.45266	0.54197
H	-0.76205	3.90229	1.11614
H	-2.37717	4.21366	0.52455
Si	-2.149	1.79411	1.35518
C	-4.05422	1.83503	1.22718
H	-4.52845	0.9117	1.57075
H	-4.43133	2.66315	1.84428
H	-4.38019	2.00609	0.1969
C	-1.83511	1.90228	3.24429
H	-2.31538	1.0581	3.75187
H	-0.77651	1.86448	3.52078
H	-2.26792	2.82953	3.6459
H	4.18531	1.50075	-1.13594
H	4.19795	-0.51228	-1.59734

Thermal correction to Energy= 1.176316

Thermal correction to Enthalpy=1.177260

Thermal correction to Gibbs Free Energy= 1.010090

Sum of electronic and zero-point Energies= -2886.256188

Sum of electronic and thermal Energies= -2886.190275

Sum of electronic and thermal Enthalpies= -2886.189331

Sum of electronic and thermal Free Energies= -2886.356501

SCF Done: E(wB97XD) = -2887.38528876

Int10a′

Number of Negative Frequencies = 0

Ir	0.55052	0.11454	0.79793
B	2.10392	0.40598	-0.62274
B	1.60786	-1.69979	0.69148
O	1.84614	0.31359	-1.98929
O	3.46552	0.56623	-0.39382
C	-3.6644	1.64637	0.69806
C	-2.77291	2.57645	2.69044
C	-1.596	1.88153	2.42626
H	-0.75376	1.94904	3.09649
C	-3.28066	-0.27842	-1.55668
N	-0.99952	-0.41494	-0.8331
N	-1.42674	1.09519	1.35598
O	2.17075	-2.43646	1.7341
O	1.77383	-2.41029	-0.50655
C	-2.25239	0.06465	-0.67282
C	-2.46203	0.96296	0.48979
O	-0.5705	-1.25109	3.36696
O	-1.10888	-2.50826	1.54739
C	-3.04436	-1.12069	-2.65037
C	-3.85348	2.47671	1.80871
C	2.2365	-3.75499	-0.21169
C	2.91025	-3.55992	1.19409
C	-1.97421	-2.98084	2.61199
C	-1.2759	-2.40014	3.89804
C	3.07907	0.41589	-2.73708
C	4.12659	0.88848	-1.64159
C	-1.7365	-1.59598	-2.78594
C	3.3735	-0.96846	-3.32538
H	3.44354	-1.71848	-2.53745
H	2.54839	-1.25614	-3.98674
H	4.29735	-0.97342	-3.9142
C	2.85388	1.41344	-3.87989
H	3.77039	1.56068	-4.46378
H	2.08427	1.02225	-4.554
H	2.51299	2.38168	-3.51018
C	4.3849	2.403	-1.64423

H	5.01765	2.65879	-0.78862	C	1.70084	3.05436	0.7861
H	4.90308	2.72209	-2.55619	C	0.95261	2.14027	0.00208
H	3.45515	2.97034	-1.55274	C	0.2003	2.64566	-1.07655
C	5.46789	0.15162	-1.66724	C	0.12808	4.01054	-1.35539
H	6.01063	0.34825	-2.5998	C	0.83218	4.9207	-0.56158
H	6.08853	0.50079	-0.83675	C	1.60164	4.41635	0.47647
H	5.3425	-0.9283	-1.5601	H	-0.32736	1.9513	-1.72245
C	3.18363	-4.22188	-1.31814	H	-0.46962	4.37082	-2.18966
H	3.55243	-5.23208	-1.10408	H	0.80653	5.99133	-0.74031
H	2.6504	-4.25566	-2.2735	H	-4.27806	0.10531	-1.38766
H	4.04501	-3.56209	-1.43268	H	-4.46237	1.54046	-0.02582
C	0.99872	-4.66226	-0.18962	H	-1.45541	-2.25596	-3.59782
H	0.28056	-4.3303	0.56149	H	-2.8169	3.19037	3.5807
H	0.506	-4.60836	-1.16726	C	-5.18482	4.06718	3.2946
H	1.2661	-5.7094	-0.00329	H	-6.1487	4.57734	3.39668
C	2.77143	-4.74738	2.14596	H	-5.04106	3.45169	4.18946
H	3.30013	-5.62435	1.75496	H	-4.40586	4.83756	3.27773
H	3.21067	-4.49353	3.11664	C	-5.42714	4.15992	0.80267
H	1.72535	-5.02079	2.31008	H	-4.62443	4.90006	0.71119
C	4.38462	-3.13913	1.11024	H	-5.48412	3.60858	-0.14211
H	5.01782	-3.9564	0.74652	H	-6.37328	4.69754	0.93143
H	4.51173	-2.2741	0.45293	C	-6.33545	2.19037	2.10272
H	4.73203	-2.85219	2.10571	H	-6.18766	1.5098	2.94857
C	-3.36666	-2.38547	2.35125	H	-7.29072	2.70732	2.24494
H	-3.6969	-2.68294	1.35109	H	-6.41841	1.58419	1.19444
H	-4.10525	-2.7416	3.07796	C	-4.73301	-0.17682	-4.2565
H	-3.34424	-1.29212	2.38909	H	-5.54725	-0.41473	-4.95287
C	-2.05273	-4.50651	2.5463	H	-5.13116	0.50996	-3.50187
H	-2.50583	-4.81198	1.59781	H	-3.95118	0.35143	-4.81359
H	-1.06379	-4.96706	2.61275	C	-3.70658	-2.40699	-4.75148
H	-2.67326	-4.90286	3.3597	H	-3.32709	-3.35891	-4.36495
C	-0.21867	-3.33353	4.50155	H	-4.54755	-2.63338	-5.418
H	0.32892	-2.79034	5.27778	H	-2.92075	-1.94429	-5.35858
H	-0.66856	-4.22308	4.95528	C	-5.31317	-2.18992	-2.84267
H	0.50374	-3.64252	3.74388	H	-4.95134	-3.11682	-2.38358
C	-2.22951	-1.92039	4.99486	H	-5.7232	-1.55938	-2.04514
H	-2.82992	-2.75052	5.38576	H	-6.13526	-2.44464	-3.52049
H	-1.65103	-1.50267	5.82495	F	2.30116	5.30702	1.23227
H	-2.9052	-1.14216	4.6325	C	2.60493	2.58588	1.89379
C	-4.18145	-1.47649	-3.62069	H	2.66667	3.33484	2.69245
C	-5.18297	3.21951	2.00843	H	3.62501	2.47641	1.49681
C	-0.75651	-1.22999	-1.86642	Si	2.02775	0.87387	2.55155
H	0.25794	-1.59618	-1.94276	C	1.2547	1.24622	4.27767
B	-0.37734	-1.40952	2.00158	H	0.78514	2.23737	4.33661

H 0.52523 0.49217 4.5826
H 2.06833 1.2509 5.01563
C 3.62333 -0.05981 3.00065
H 3.4185 -1.00145 3.51895
H 4.22244 -0.27709 2.11222
H 4.22185 0.57857 3.66769

Thermal correction to Energy= 1.177363
Thermal correction to Enthalpy= 1.178308
Thermal correction to Gibbs Free Energy= 1.013713
Sum of electronic and zero-point Energies= -2886.286525
Sum of electronic and thermal Energies= -2886.220663
Sum of electronic and thermal Enthalpies= -2886.219719
Sum of electronic and thermal Free Energies= -2886.384313
SCF Done: E(wB97XD) = -2887.424276

TS11a′

Number of Negative Frequencies = 1

Ir 0.51672 0.03113 0.82888
B 2.02017 0.4329 -0.58865
B 1.63137 -1.74001 0.67763
O 1.77204 0.30807 -1.96002
O 3.38338 0.63468 -0.36949
C -3.60409 1.74814 0.63748
C -2.74952 2.56397 2.6933
C -1.62328 1.77502 2.47998
H -0.82161 1.74784 3.20309
C -3.302 -0.28482 -1.54176
N -1.02384 -0.46053 -0.81947
N -1.45404 1.00869 1.39743
O 2.21934 -2.46055 1.71742
O 1.79791 -2.45148 -0.51967
C -2.26405 0.0494 -0.66738
C -2.45591 0.96352 0.48503
O -0.66448 -1.28946 3.36528
O -0.98366 -2.67778 1.5872
C -3.08453 -1.14316 -2.62722
C -3.77467 2.58843 1.74435
C 2.28256 -3.79024 -0.22691
C 2.95223 -3.59024 1.18205
C -1.87089 -3.16828 2.62544
C -1.30233 -2.46776 3.91527
C 3.00275 0.43729 -2.7046
C 4.02082 0.98671 -1.61954
C -1.78562 -1.6412 -2.76392
C 3.36306 -0.95109 -3.24541
H 3.475 -1.66636 -2.43175
H 2.5492 -1.30367 -3.88881
H 4.28282 -0.93022 -3.84035
C 2.7465 1.38627 -3.88034
H 3.66317 1.55895 -4.45724
H 2.00578 0.93861 -4.5523
H 2.35599 2.34968 -3.54454
C 4.17942 2.51497 -1.64083
H 4.77888 2.8205 -0.77717
H 4.69154 2.85356 -2.54855
H 3.21361 3.02246 -1.5738
C 5.40648 0.33643 -1.64206
H 5.92967 0.55052 -2.58173
H 6.009 0.7384 -0.82077
H 5.34787 -0.7473 -1.51652
C 3.23818 -4.23919 -1.33401
H 3.61938 -5.24636 -1.12561
H 2.70613 -4.27332 -2.29051
H 4.09158 -3.56817 -1.44573
C 1.06067 -4.71935 -0.21346
H 0.32904 -4.39615 0.52856
H 0.57936 -4.68016 -1.19673
H 1.34614 -5.75926 -0.01865
C 2.7978 -4.76941 2.14232
H 3.31222 -5.65823 1.75816
H 3.24216 -4.51423 3.11008
H 1.74785 -5.02239 2.30697
C 4.42945 -3.17922 1.10251
H 5.05922 -4.00398 0.75033
H 4.56525 -2.321 0.43769
H 4.77271 -2.88432 2.09803
C -3.28677 -2.70364 2.24918
H -3.52961 -3.08375 1.25184
H -4.0397 -3.07598 2.95251
H -3.34898 -1.61067 2.21801
C -1.82406 -4.69668 2.64383
H -2.20261 -5.09081 1.69458
H -0.80721 -5.07016 2.78252
H -2.45166 -5.09681 3.44917
C -0.20552 -3.2753 4.62311
H 0.24311 -2.65197 5.4034
H -0.60327 -4.18106 5.0948
H 0.58729 -3.55251 3.9241

C	-2.35566	-2.0218	4.92997
H	-2.90329	-2.88259	5.33228
H	-1.8656	-1.51371	5.76675
H	-3.07473	-1.32632	4.48978
C	-4.2334	-1.49027	-3.5856
C	-5.02147	3.47724	1.86307
C	-0.79752	-1.2878	-1.84583
H	0.21138	-1.66919	-1.9217
B	-0.37542	-1.50048	2.02435
C	1.53555	3.03253	0.87169
C	0.88683	2.10812	0.01737
C	0.13127	2.60135	-1.06337
C	-0.03446	3.96852	-1.28403
C	0.56768	4.88857	-0.42171
C	1.33841	4.39599	0.6225
H	-0.31233	1.89697	-1.75884
H	-0.62486	4.32188	-2.12649
H	0.46863	5.96176	-0.55393
H	-4.29337	0.11348	-1.3653
H	-4.36189	1.72659	-0.13523
H	-1.51824	-2.31192	-3.57184
H	-2.79234	3.16467	3.59342
C	-5.01545	4.31253	3.15768
H	-5.92288	4.92442	3.20418
H	-4.99841	3.68	4.05248
H	-4.15759	4.99247	3.20006
C	-5.0642	4.44603	0.65698
H	-4.16875	5.07619	0.6266
H	-5.12911	3.91048	-0.29646
H	-5.94067	5.10042	0.73178
C	-6.28867	2.58961	1.85994
H	-6.28141	1.8885	2.70219
H	-7.18532	3.21437	1.94626
H	-6.37848	2.00571	0.93761
C	-4.77026	-0.18861	-4.22925
H	-5.59464	-0.41859	-4.91405
H	-5.14771	0.51321	-3.4781
H	-3.98582	0.32092	-4.80003
C	-3.78153	-2.43826	-4.71174
H	-3.4098	-3.39077	-4.31827
H	-4.63079	-2.66042	-5.36753
H	-2.99508	-1.99068	-5.32958
C	-5.37013	-2.17968	-2.79284
H	-5.01863	-3.10593	-2.32544
H	-5.76753	-1.53499	-2.00186
H	-6.19944	-2.43107	-3.46463
F	1.93541	5.30052	1.44487
C	2.44128	2.58318	1.98686
H	2.40465	3.29084	2.82472
H	3.47883	2.60117	1.62217
Si	2.03608	0.78776	2.5528
C	1.36695	0.9749	4.34618
H	0.55287	1.70124	4.44289
H	1.00403	0.02303	4.74319
H	2.18854	1.33528	4.98068
C	3.72649	-0.03472	2.8553
H	3.6242	-1.00799	3.34407
H	4.28039	-0.16759	1.92296
H	4.3146	0.62014	3.51403

Thermal correction to Energy= 1.176131

Thermal correction to Enthalpy= 1.177075

Thermal correction to Gibbs Free Energy= 1.014072

Sum of electronic and zero-point Energies= -2886.286616

Sum of electronic and thermal Energies= -2886.221463

Sum of electronic and thermal Enthalpies= -2886.220519

Sum of electronic and thermal Free Energies= -2886.383522

SCF Done: E(wB97XD) = -2887.421571

Int12a′

Number of Negative Frequencies =0

Ir	0.54452	0.10254	0.80693
B	2.07992	0.48998	-0.63474
B	1.62886	-1.68479	0.67948
O	1.83714	0.34359	-2.00429
O	3.45052	0.60823	-0.40282
C	-3.6704	1.64937	0.69806
C	-2.77291	2.59745	2.67844
C	-1.593	1.91153	2.41126
H	-0.74476	1.99104	3.07549
C	-3.27766	-0.26942	-1.55968
N	-1.00252	-0.42094	-0.8241
N	-1.42374	1.12519	1.34098
O	2.17674	-2.43046	1.7251
O	1.79783	-2.39829	-0.51855
C	-2.25239	0.07065	-0.67282
C	-2.46503	0.97496	0.48379
O	-0.5855	-1.23909	3.38196
O	-1.10588	-2.49926	1.55639

C	-3.04136	-1.1177	-2.65037
C	-3.85948	2.48571	1.80571
C	2.2455	-3.74599	-0.22069
C	2.91325	-3.55692	1.18809
C	-1.97421	-2.97784	2.61799
C	-1.2819	-2.39714	3.90704
C	3.07307	0.42189	-2.74608
C	4.12359	0.90048	-1.65059
C	-1.7365	-1.60198	-2.77994
C	3.3585	-0.97446	-3.31338
H	3.41354	-1.71248	-2.51345
H	2.53639	-1.26214	-3.97774
H	4.28835	-0.99742	-3.8932
C	2.86588	1.40444	-3.90389
H	3.78539	1.53068	-4.48778
H	2.09327	1.01325	-4.575
H	2.53699	2.38468	-3.54918
C	4.4029	2.412	-1.68023
H	5.03565	2.67079	-0.82462
H	4.92708	2.70709	-2.59519
H	3.48215	2.99434	-1.60074
C	5.45589	0.14562	-1.65824
H	6.00463	0.32125	-2.5908
H	6.07653	0.50079	-0.82775
H	5.3155	-0.9313	-1.5361
C	3.19263	-4.22488	-1.32114
H	3.55243	-5.23808	-1.10408
H	2.6624	-4.25566	-2.2795
H	4.06001	-3.57109	-1.43568
C	0.99872	-4.64426	-0.20162
H	0.28056	-4.3063	0.54949
H	0.509	-4.58736	-1.17926
H	1.2571	-5.6914	-0.01229
C	2.76543	-4.74438	2.13996
H	3.29113	-5.62435	1.74896
H	3.20467	-4.49353	3.11064
H	1.71935	-5.0118	2.29809
C	4.39062	-3.14213	1.10724
H	5.02082	-3.9624	0.74652
H	4.52074	-2.2771	0.44993
H	4.73503	-2.85519	2.10571
C	-3.36666	-2.38547	2.35425
H	-3.6939	-2.67994	1.35109
H	-4.10525	-2.7476	3.07796
H	-3.34724	-1.29212	2.39509
C	-2.04673	-4.50351	2.5493
H	-2.49683	-4.80898	1.59781
H	-1.05779	-4.96106	2.61575
H	-2.66726	-4.90286	3.3597
C	-0.21567	-3.32453	4.50455
H	0.32892	-2.78134	5.28378
H	-0.65956	-4.22008	4.95528
H	0.50974	-3.62452	3.74388
C	-2.23851	-1.92939	5.00386
H	-2.83292	-2.76552	5.39176
H	-1.66303	-1.51167	5.83695
H	-2.9202	-1.15416	4.6445
C	-4.17845	-1.47349	-3.62069
C	-5.19197	3.22251	2.00843
C	-0.75951	-1.23899	-1.85442
H	0.25494	-1.60818	-1.92476
B	-0.38634	-1.39452	2.01658
C	1.73384	3.03336	0.7681
C	1.02161	2.11627	-0.04892
C	0.2453	2.62766	-1.10955
C	0.13108	3.99554	-1.35239
C	0.81118	4.9027	-0.53758
C	1.59864	4.39834	0.48847
H	-0.25236	1.9303	-1.77345
H	-0.48162	4.35882	-2.17466
H	0.75853	5.97633	-0.69231
H	-4.27506	0.12331	-1.39966
H	-4.47437	1.53146	-0.01682
H	-1.45541	-2.26496	-3.58882
H	-2.8169	3.21737	3.5657
C	-5.19382	4.07018	3.2946
H	-6.1607	4.57434	3.39968
H	-5.04406	3.45469	4.18946
H	-4.41786	4.84356	3.27473
C	-5.43914	4.15992	0.80267
H	-4.63943	4.90306	0.71119
H	-5.49312	3.60858	-0.14211
H	-6.38828	4.69454	0.93143
C	-6.33845	2.18737	2.10272
H	-6.18766	1.5068	2.94857
H	-7.29672	2.70132	2.24794
H	-6.42141	1.58119	1.19444
C	-4.73001	-0.17682	-4.2595

H	-5.54125	-0.41473	-4.95587
H	-5.12816	0.51296	-3.50787
H	-3.94818	0.35143	-4.81659
C	-3.70358	-2.40699	-4.75148
H	-3.32409	-3.35891	-4.36195
H	-4.54155	-2.63338	-5.418
H	-2.91475	-1.94429	-5.35558
C	-5.31017	-2.18692	-2.84267
H	-4.94834	-3.11383	-2.38358
H	-5.7202	-1.55638	-2.04814
H	-6.13226	-2.44164	-3.52349
F	2.27416	5.29202	1.25927
C	2.62893	2.56188	1.88179
H	2.69667	3.31984	2.67145
H	3.64601	2.43441	1.48781
Si	2.02775	0.86187	2.55455
C	1.2547	1.25522	4.27767
H	0.79114	2.24937	4.32761
H	0.51923	0.50717	4.5826
H	2.06533	1.2599	5.01863
C	3.62333	-0.07181	3.01265
H	3.4185	-1.01345	3.52795
H	4.22244	-0.28909	2.12422
H	4.21885	0.56657	3.67969

Thermal correction to Energy= 1.178467

Thermal correction to Enthalpy= 1.179411

Thermal correction to Gibbs Free Energy= 1.010337

Sum of electronic and zero-point Energies= -2886.321047

Sum of electronic and thermal Energies= -2886.254744

Sum of electronic and thermal Enthalpies= -2886.253800

Sum of electronic and thermal Free Energies= -2886.422874

SCF Done: E(wB97XD) = -2887.441017

TS13a′

Number of Negative Frequencies =1

B	-2.80222	-0.12482	-1.36373
B	-1.60865	-1.8352	0.47816
B	0.21484	-1.39237	-1.6916
C	0.7411	2.06351	-2.15731
H	1.11843	1.15567	-2.61389
C	1.12967	3.32101	-2.60522
C	0.64973	4.47347	-1.96987
C	-0.20497	4.26244	-0.88331
C	-0.56018	2.9723	-0.47229
C	-1.44695	2.71111	0.68136
C	-1.95354	3.73384	1.49024
C	-2.81565	3.46674	2.55845
C	-3.13473	2.11904	2.76116
C	-2.58857	1.13763	1.94246
H	-2.82454	0.0891	2.07907
C	-4.75424	-0.70459	-2.44515
C	-4.94554	0.65488	-1.6806
C	-2.08374	-4.05573	0.91792
C	-3.01145	-3.14936	1.81019
C	1.7326	-1.91063	-3.37104
C	0.90057	-3.18608	-2.97691
Ir	-0.86716	-0.1204	-0.40673
N	-0.09461	1.87866	-1.12391
N	-1.7516	1.41023	0.92774
O	-3.5109	-1.1973	-1.88409
O	-3.57116	1.03013	-1.3855
O	-1.07852	-3.11689	0.47497
O	-2.81125	-1.84067	1.21605
O	1.42783	-1.00148	-2.28026
O	-0.18074	-2.61397	-2.21048
H	-1.2532	-0.4993	-1.92586
C	2.23766	1.15766	1.17266
C	-5.84757	-1.74553	-2.2072
H	-6.81862	-1.38505	-2.56758
H	-5.60485	-2.66344	-2.75255
H	-5.93987	-1.99798	-1.14844
C	-4.52127	-0.52627	-3.95325
H	-4.19503	-1.48201	-4.37505
H	-5.43372	-0.21247	-4.47278
H	-3.73774	0.21324	-4.14771
C	-5.59244	1.77675	-2.4909
H	-6.6098	1.50376	-2.79571
H	-5.65588	2.68313	-1.87924
H	-5.01286	2.01594	-3.38595
C	-5.65895	0.49409	-0.33013
H	-5.56634	1.42978	0.23084
H	-6.7251	0.27287	-0.45388
H	-5.19754	-0.30243	0.26172
C	0.30235	-3.96084	-4.15286
H	-0.27305	-4.81137	-3.77328
H	-0.37285	-3.339	-4.74544
H	1.08945	-4.35083	-4.80944
C	1.65895	-4.15154	-2.05356

H	0.94953	-4.88277	-1.65467
H	2.44913	-4.69035	-2.58897
H	2.10315	-3.62643	-1.20416
C	1.24264	-1.24559	-4.66694
H	1.74017	-0.2766	-4.78522
H	1.47319	-1.85293	-5.54911
H	0.16204	-1.07266	-4.63928
C	3.246	-2.11289	-3.43089
H	3.51236	-2.86672	-4.18149
H	3.73307	-1.1728	-3.71304
H	3.64503	-2.42184	-2.46235
C	-2.56134	-3.07411	3.27625
H	-3.11144	-2.27143	3.78025
H	-2.76888	-4.01001	3.80777
H	-1.49289	-2.86365	3.35817
C	-4.50266	-3.48192	1.74587
H	-4.69586	-4.49651	2.11436
H	-5.06543	-2.78404	2.37636
H	-4.88642	-3.40324	0.72618
C	-1.39151	-5.20637	1.65017
H	-2.12637	-5.90054	2.07616
H	-0.77357	-5.76718	0.94065
H	-0.73841	-4.84562	2.44635
C	-2.78641	-4.58528	-0.34257
H	-2.03519	-5.03628	-0.99808
H	-3.53664	-5.34635	-0.09791
H	-3.26344	-3.77366	-0.8989
Si	0.97347	-0.27847	1.32841
C	0.34721	-0.10606	3.13293
H	-0.04901	0.90358	3.29865
H	-0.44786	-0.81614	3.38114
H	1.1667	-0.25485	3.84703
C	2.04728	-1.90182	1.21584
H	2.70008	-1.76481	0.35022
H	1.34723	-2.7134	0.9961
H	-3.80226	1.80701	3.55504
H	1.80571	3.37915	-3.44965
C	1.02213	5.89969	-2.40465
H	-1.66961	4.75779	1.28241
H	-0.60626	5.11596	-0.35129
C	-3.36006	4.60928	3.42962
C	-4.30254	4.09447	4.53401
H	-4.66473	4.93882	5.13077
H	-5.17894	3.58427	4.11837
H	-3.79349	3.40309	5.21482
C	-2.17754	5.34679	4.10273
H	-1.60169	4.66725	4.74079
H	-1.49057	5.7771	3.36624
H	-2.55143	6.16625	4.72807
C	-4.14766	5.60118	2.54022
H	-4.99101	5.10442	2.04742
H	-4.54468	6.4214	3.15011
H	-3.51704	6.04178	1.76085
C	1.752	6.61307	-1.24105
H	2.02172	7.63469	-1.53415
H	1.1266	6.67855	-0.34447
H	2.67151	6.08262	-0.96946
C	-0.26585	6.6795	-2.76178
H	-0.01472	7.70025	-3.07377
H	-0.80425	6.1952	-3.58423
H	-0.95032	6.75131	-1.91
C	1.9491	5.90608	-3.63509
H	2.9	5.40051	-3.43249
H	1.48108	5.42474	-4.50122
H	2.17897	6.9396	-3.91633
C	2.85174	-2.23718	2.43826
C	4.2129	-1.88731	2.65047
C	2.22422	-2.92924	3.48522
C	4.83562	-2.22814	3.87174
C	2.83341	-3.27928	4.67849
C	4.1674	-2.91817	4.87736
H	5.87333	-1.94409	4.01745
H	2.26132	-3.82062	5.42591
H	4.67047	-3.17544	5.80535
F	0.91897	-3.29005	3.32457
C	6.90921	0.09375	0.90505
C	6.0988	-0.45047	-0.33442
B	5.13173	-1.1675	1.61753
O	6.37788	-0.70238	1.99252
O	4.87622	-0.9393	0.28308
C	5.7262	0.60182	-1.3778
H	6.62485	1.03025	-1.8374
H	5.12721	0.13846	-2.16765
H	5.1358	1.41035	-0.94122
C	6.76315	-1.65644	-1.01396
H	6.06173	-2.09234	-1.73179
H	7.67177	-1.36707	-1.55284
H	7.02308	-2.42951	-0.28404

C	6.60242	1.56102	1.24036
H	7.04369	1.80011	2.21318
H	7.02268	2.244	0.49411
H	5.52414	1.73642	1.30681
C	8.42166	-0.12013	0.84169
H	8.86017	0.42658	-0.00139
H	8.88198	0.25304	1.76247
H	8.67789	-1.17748	0.74345
H	3.01251	1.0758	1.94696
H	2.73241	1.14374	0.19563
H	1.75305	2.13445	1.29749

Thermal correction to Energy= 1.379473

Thermal correction to Enthalpy= 1.380417

Thermal correction to Gibbs Free Energy= 1.191299

Sum of electronic and zero-point Energies= -3297.986354

Sum of electronic and thermal Energies= -3297.909864

Sum of electronic and thermal Enthalpies= -3297.908920

Sum of electronic and thermal Free Energies= -3298.098038

SCF Done: E(wB97XD) = -3299.354935

Int14a′

Number of Negative Frequencies =0

B	-2.91897	-0.04196	-1.33012
B	-1.69178	-1.83487	0.41841
B	0.32204	-1.38253	-1.64355
C	0.47892	2.06612	-2.37526
H	0.83495	1.16648	-2.86441
C	0.80094	3.3312	-2.85198
C	0.36468	4.47311	-2.16771
C	-0.38285	4.24302	-1.00903
C	-0.67906	2.94486	-0.57666
C	-1.46612	2.66204	0.64044
C	-1.9242	3.67152	1.49382
C	-2.69922	3.38857	2.62142
C	-2.98932	2.03534	2.83486
C	-2.49405	1.06624	1.97286
H	-2.71627	0.01642	2.11325
C	-4.85465	-0.50768	-2.50365
C	-5.08281	0.72927	-1.561
C	-2.17774	-4.04518	0.88913
C	-3.10691	-3.12495	1.76281
C	1.87693	-1.81602	-3.31752
C	1.19085	-3.16232	-2.85772
Ir	-0.94303	-0.13543	-0.48561
N	-0.25398	1.8613	-1.27034
N	-1.73314	1.35257	0.9021
O	-3.60247	-1.04605	-2.0057
O	-3.72023	1.08481	-1.19556
O	-1.16819	-3.11754	0.43843
O	-2.90192	-1.82666	1.14731
O	1.46325	-0.88136	-2.28774
O	0.10012	-2.68991	-2.04153
H	-1.25404	-0.70501	-1.96965
C	2.30908	1.14277	0.77621
C	-5.92188	-1.59913	-2.41936
H	-6.90002	-1.21796	-2.73582
H	-5.65113	-2.42824	-3.08232
H	-6.01159	-1.99406	-1.40572
C	-4.61802	-0.11917	-3.97147
H	-4.26571	-1.00028	-4.51699
H	-5.53473	0.23966	-4.45282
H	-3.85318	0.65868	-4.05392
C	-5.74184	1.93995	-2.21927
H	-6.75428	1.69923	-2.56255
H	-5.81752	2.75631	-1.49163
H	-5.16222	2.30163	-3.07182
C	-5.81268	0.36893	-0.2587
H	-5.77742	1.23096	0.41755
H	-6.86322	0.11629	-0.438
H	-5.32421	-0.47185	0.24175
C	0.60079	-4.00523	-3.99221
H	0.11443	-4.88984	-3.56769
H	-0.15068	-3.45404	-4.56145
H	1.38334	-4.34675	-4.68093
C	2.08444	-4.05043	-1.98196
H	1.47068	-4.8499	-1.55492
H	2.89291	-4.50748	-2.56314
H	2.52042	-3.49094	-1.15229
C	1.33681	-1.28042	-4.65364
H	1.74391	-0.27943	-4.83062
H	1.62832	-1.91666	-5.49595
H	0.24445	-1.20615	-4.63829
C	3.40478	-1.84837	-3.35652
H	3.7636	-2.59367	-4.07784
H	3.78701	-0.86914	-3.66733
H	3.82305	-2.07737	-2.37409
C	-2.66011	-3.02159	3.22923

H	-3.22701	-2.22267	3.72119
H	-2.85217	-3.95326	3.77419
H	-1.59516	-2.79062	3.30992
C	-4.59807	-3.45969	1.70106
H	-4.78919	-4.46716	2.08983
H	-5.16416	-2.75037	2.31523
H	-4.97902	-3.4029	0.67834
C	-1.49623	-5.18723	1.64404
H	-2.23487	-5.86673	2.083
H	-0.88067	-5.76537	0.94664
H	-0.84251	-4.81099	2.43198
C	-2.87575	-4.58789	-0.36991
H	-2.11746	-5.03505	-1.01856
H	-3.62215	-5.35131	-0.12552
H	-3.35741	-3.78119	-0.93082
Si	1.03491	-0.24358	1.13697
C	0.52077	0.08556	2.95528
H	0.12617	1.10095	3.06951
H	-0.24178	-0.61226	3.31862
H	1.39019	-0.00243	3.61798
C	2.07885	-1.89468	1.13852
H	2.79468	-1.79298	0.31958
H	1.38333	-2.70251	0.89299
H	-3.59299	1.70898	3.67393
H	1.39621	3.40279	-3.75403
C	0.67053	5.90694	-2.62745
H	-1.67187	4.70056	1.26967
H	-0.74645	5.08753	-0.43632
C	-3.18703	4.51907	3.54044
C	-4.02805	3.98448	4.71475
H	-4.3526	4.82024	5.34371
H	-4.92814	3.46265	4.36739
H	-3.45468	3.29636	5.3464
C	-1.96434	5.26969	4.11835
H	-1.32778	4.59341	4.70016
H	-1.34688	5.714	3.32932
H	-2.29542	6.07853	4.778
C	-4.0594	5.50207	2.72326
H	-4.93381	4.99633	2.30061
H	-4.41421	6.31535	3.36664
H	-3.50174	5.95314	1.89442
C	1.46872	6.63739	-1.52225
H	1.68963	7.66665	-1.8345
H	0.91162	6.688	-0.58132

H	2.42011	6.13136	-1.32245
C	-0.65755	6.65605	-2.88808
H	-0.45447	7.68313	-3.21301
H	-1.24302	6.15927	-3.67067
H	-1.28025	6.70997	-1.9877
C	1.50317	5.93209	-3.92507
H	2.47222	5.43861	-3.79755
H	0.97667	5.44948	-4.75555
H	1.69694	6.97026	-4.2156
C	2.79553	-2.21917	2.41749
C	4.15709	-1.92105	2.70241
C	2.07671	-2.85312	3.44162
C	4.68974	-2.25693	3.96673
C	2.59714	-3.19723	4.67658
C	3.93362	-2.89184	4.94573
H	5.72813	-2.01382	4.16861
H	1.95823	-3.69221	5.4013
H	4.36835	-3.14543	5.90859
F	0.76652	-3.15396	3.21185
C	7.04983	-0.09978	1.10662
C	6.28358	-0.60254	-0.17903
B	5.16892	-1.26208	1.71557
O	6.4134	-0.86105	2.16041
O	5.00419	-1.02818	0.36792
C	6.03	0.46887	-1.23849
H	6.97657	0.85873	-1.63145
H	5.46914	0.035	-2.07177
H	5.44454	1.29928	-0.83808
C	6.92347	-1.84126	-0.82098
H	6.24414	-2.24042	-1.58025
H	7.87645	-1.59906	-1.30351
H	7.09905	-2.62761	-0.07857
C	6.80576	1.383	1.42415
H	7.19883	1.60006	2.42183
H	7.3099	2.03964	0.70547
H	5.73732	1.62172	1.4222
C	8.54922	-0.39748	1.12963
H	9.06615	0.12152	0.31403
H	8.97518	-0.04849	2.07602
H	8.75015	-1.46788	1.04511
H	3.13523	1.1124	1.50054
H	2.73406	1.02557	-0.22578
H	1.85187	2.13781	0.83941

Thermal correction to Energy= 1.382399

Thermal correction to Enthalpy= 1.383343

Thermal correction to Gibbs Free Energy= 1.189766

Sum of electronic and zero-point Energies= -3298.004798

Sum of electronic and thermal Energies= -3297.927291

Sum of electronic and thermal Enthalpies= -3297.926347

Sum of electronic and thermal Free Energies= -3298.119923

SCF Done: E(wB97XD) = -3299.365631

TS15a′

Number of Negative Frequencies =1

B	-2.61576	0.03838	1.27225
B	-1.39064	-2.13425	-0.24039
B	-2.87606	0.06746	-1.32223
C	-1.02398	3.06135	-0.90287
H	-1.6965	2.72599	-1.68495
C	-0.58083	4.37823	-0.82691
C	0.30422	4.76421	0.18683
C	0.6559	3.77241	1.10885
C	0.17067	2.46677	0.98802
C	0.54006	1.38137	1.92505
C	1.20183	1.62187	3.1331
C	1.55864	0.57734	3.99307
C	1.19492	-0.70896	3.57553
C	0.51478	-0.89505	2.37748
H	0.18492	-1.87541	2.05279
C	-4.10136	-0.48689	2.96451
C	-4.06187	1.07354	2.75971
C	-2.17382	-4.2348	-0.83172
C	-1.2452	-4.36551	0.43527
C	-4.43532	1.13681	-2.66788
C	-4.9446	-0.3006	-2.28756
Ir	-1.11011	-0.10483	-0.22798
N	-0.64663	2.11494	-0.03234
N	0.19359	0.11935	1.55502
O	-2.9401	-0.92427	2.21701
O	-3.35403	1.2021	1.50166
O	-1.87363	-2.90403	-1.30252
O	-1.07049	-2.98639	0.82844
O	-3.03492	1.05936	-2.3002
O	-4.05424	-0.66159	-1.20379
H	-0.86076	-0.26309	-1.86017
C	2.47387	0.39643	-0.7887
C	-3.95421	-0.9573	4.41193
H	-4.78696	-0.59983	5.02982
H	-3.9561	-2.05194	4.4431
H	-3.01718	-0.61191	4.85541
C	-5.32346	-1.15623	2.31888
H	-5.19263	-2.24256	2.36102
H	-6.25331	-0.90296	2.84074
H	-5.41199	-0.87263	1.26661
C	-5.42732	1.74862	2.62666
H	-6.01798	1.62818	3.54282
H	-5.29271	2.8213	2.45036
H	-5.99799	1.33838	1.79024
C	-3.22356	1.80729	3.81866
H	-3.07988	2.84457	3.49853
H	-3.71546	1.81433	4.79794
H	-2.23577	1.34859	3.92862
C	-6.38562	-0.36679	-1.78177
H	-6.62878	-1.39703	-1.50118
H	-6.53511	0.26668	-0.90406
H	-7.0909	-0.05347	-2.56099
C	-4.73781	-1.33143	-3.4076
H	-4.92917	-2.33145	-3.00728
H	-5.41853	-1.15953	-4.2491
H	-3.70813	-1.31275	-3.77615
C	-5.06642	2.24894	-1.81641
H	-4.54024	3.18963	-2.01212
H	-6.12506	2.3968	-2.05807
H	-4.97051	2.02664	-0.74954
C	-4.53101	1.49125	-4.15162
H	-5.57444	1.48869	-4.48912
H	-4.12624	2.49538	-4.31886
H	-3.96255	0.79288	-4.77016
C	0.14518	-4.93688	0.11981
H	0.78843	-4.81255	0.99779
H	0.10233	-6.0049	-0.12239
H	0.60876	-4.40788	-0.71709
C	-1.85444	-5.12696	1.61498
H	-2.07526	-6.16691	1.34515
H	-1.14484	-5.13984	2.44982
H	-2.77309	-4.65098	1.96401
C	-1.88212	-5.23035	-1.95627
H	-2.04687	-6.26271	-1.62422
H	-2.55421	-5.03595	-2.79882
H	-0.85572	-5.14075	-2.32039
C	-3.6711	-4.26826	-0.48604
H	-4.24495	-4.00618	-1.3799

H	-3.99006	-5.2612	-0.14867
H	-3.91082	-3.53354	0.28752
Si	0.93952	-0.23044	-1.81989
C	1.37119	-1.98578	-2.41377
H	1.68148	-2.61536	-1.57331
H	0.50203	-2.45572	-2.8862
H	2.19294	-1.96251	-3.14165
C	0.83911	0.84718	-3.39544
H	0.6221	1.89659	-3.16734
H	1.77704	0.81643	-3.96276
H	0.0373	0.47847	-4.04702
H	1.41999	-1.58344	4.1739
H	-0.9179	5.07925	-1.58052
C	0.91325	6.16985	0.29025
H	1.4299	2.64394	3.40976
H	1.34854	4.00927	1.90697
C	2.29705	0.86557	5.30905
C	2.58891	-0.42253	6.10204
H	3.11531	-0.17167	7.0296
H	1.66835	-0.94965	6.37671
H	3.22562	-1.11354	5.53806
C	3.6436	1.56097	4.99483
H	4.27866	0.92499	4.36801
H	3.49956	2.51077	4.46918
H	4.18485	1.77306	5.92463
C	1.43072	1.7954	6.19192
H	0.47039	1.32649	6.43426
H	1.95023	2.01263	7.13277
H	1.22204	2.75109	5.69957
C	2.45252	6.04842	0.16634
H	2.91162	7.04429	0.15909
H	2.88426	5.49019	1.0042
H	2.73127	5.52651	-0.75498
C	0.54787	6.79821	1.6556
H	0.99896	7.79354	1.74613
H	-0.53755	6.90633	1.76267
H	0.90866	6.19345	2.49457
C	0.40651	7.10328	-0.82544
H	0.6727	6.73053	-1.82085
H	-0.68106	7.23432	-0.78631
H	0.8621	8.09298	-0.71108
H	2.14615	1.23267	-0.16861
H	2.76401	-0.42152	-0.12586
C	3.63585	0.84207	-1.63101
C	4.72085	0.01463	-2.02506
C	3.65681	2.16331	-2.10069
C	5.71487	0.53799	-2.87907
C	4.63871	2.68666	-2.92598
C	5.68412	1.85413	-3.32988
H	6.53256	-0.11076	-3.1784
H	4.57082	3.72356	-3.24084
H	6.46366	2.23585	-3.9832
F	2.64732	3.00001	-1.71963
C	5.83014	-3.56189	-1.5026
C	5.10822	-3.25839	-0.13546
B	4.96579	-1.44003	-1.51844
O	4.35047	-2.05506	-0.44938
O	5.93237	-2.23855	-2.09216
C	6.07838	-2.88211	0.99388
H	5.50353	-2.48899	1.83829
H	6.65181	-3.74862	1.34019
H	6.78095	-2.10631	0.67333
C	4.1384	-4.33557	0.34435
H	4.66782	-5.27489	0.54271
H	3.66175	-4.01254	1.27564
H	3.3503	-4.52652	-0.38656
C	7.23695	-4.14644	-1.38122
H	7.21329	-5.12371	-0.88507
H	7.66247	-4.28552	-2.38036
H	7.9034	-3.48618	-0.8213
C	4.98711	-4.41227	-2.46352
H	5.46117	-4.40497	-3.45002
H	4.91279	-5.45127	-2.12503
H	3.97611	-4.00774	-2.57249

Thermal correction to Energy= 1.378738

Thermal correction to Enthalpy= 1.379682

Thermal correction to Gibbs Free Energy= 1.186275

Sum of electronic and zero-point Energies= -3297.994979

Sum of electronic and thermal Energies= -3297.917695

Sum of electronic and thermal Enthalpies= -3297.916751

Sum of electronic and thermal Free Energies= -3298.110159

SCF Done: E(wB97XD) = -3299.35919

TS2b′

Number of Negative Frequencies =1

B	-1.2299	-1.6093	0.9418
B	-2.17773	-0.29271	-1.42168
B	-1.83363	0.87973	1.193

C	1.51993	0.91483	2.14699	H	-4.04919	-3.75987	2.96286
H	0.67788	1.55455	2.38217	H	-3.74798	-2.08423	2.4251
C	2.67973	0.91889	2.91836	C	-1.58125	-3.21846	4.09731
C	3.74957	0.08467	2.58054	H	-1.91106	-4.21666	4.40969
C	3.56597	-0.72374	1.45166	H	-0.7205	-2.93853	4.71428
C	2.37649	-0.68316	0.71742	H	-2.38612	-2.50808	4.30015
C	2.14291	-1.53609	-0.47583	C	0.07048	-4.07813	2.4202
C	3.11377	-2.40364	-0.98394	H	0.88888	-3.66679	3.02037
C	2.86279	-3.21259	-2.09934	H	-0.09764	-5.11525	2.73154
C	1.58675	-3.10452	-2.65983	H	0.3869	-4.07665	1.37231
C	0.65636	-2.22195	-2.11682	C	-4.65615	0.82419	3.38035
H	-0.34301	-2.12504	-2.52656	H	-5.49082	0.38464	2.82424
C	-2.33908	-3.53256	1.60693	H	-4.16508	0.02381	3.93913
C	-1.17977	-3.20698	2.62192	H	-5.06951	1.54526	4.09593
C	-4.32896	-0.27482	-2.2678	C	-4.46642	2.49493	1.51844
C	-3.40262	-1.09736	-3.23855	H	-5.21281	1.94197	0.94043
C	-2.41149	2.13212	3.05957	H	-4.97992	3.26016	2.11154
C	-3.69548	1.50594	2.4064	H	-3.79782	2.98808	0.80698
Ir	-0.5608	0.03969	-0.20949	C	-1.91906	1.35821	4.29233
N	1.36214	0.13569	1.07153	H	-0.93707	1.74397	4.58765
N	0.92121	-1.44461	-1.05654	H	-2.59708	1.4756	5.14513
O	-2.02964	-2.65576	0.49529	H	-1.80842	0.29287	4.06867
O	-0.81269	-1.8562	2.25207	C	-2.50148	3.62463	3.37739
O	-3.36925	0.42624	-1.44925	H	-3.29063	3.82325	4.11271
O	-2.20266	-1.25849	-2.44248	H	-1.55222	3.97035	3.80106
O	-1.42747	1.94275	2.01159	H	-2.70306	4.21515	2.48079
O	-3.12893	0.50283	1.53189	C	-3.01454	-0.32607	-4.50901
H	-0.93956	1.57871	-0.62325	H	-2.2212	-0.87267	-5.03086
C	0.32257	1.56776	-1.57832	H	-3.86248	-0.22116	-5.19507
C	0.27328	1.33344	-2.96562	H	-2.63667	0.67216	-4.26853
C	1.03916	2.08275	-3.86035	C	-3.92189	-2.48608	-3.61456
H	0.99484	1.86526	-4.92506	H	-4.87501	-2.41891	-4.15282
C	1.86053	3.111	-3.39608	H	-3.20056	-2.98558	-4.2712
H	2.45585	3.69657	-4.09323	H	-4.06162	-3.11217	-2.73067
C	1.92214	3.41113	-2.02926	C	-5.23611	0.75062	-2.9484
C	1.13662	2.62625	-1.17609	H	-5.94567	0.26109	-3.62647
C	5.06377	0.02413	3.37488	H	-5.81207	1.28994	-2.18893
C	3.95263	-4.15092	-2.63948	H	-4.65983	1.48538	-3.51551
C	-2.37001	-4.97324	1.09376	C	-5.15614	-1.16351	-1.32524
H	-2.55316	-5.67997	1.91232	H	-5.61268	-0.52969	-0.55881
H	-3.1804	-5.08363	0.36557	H	-5.95292	-1.69701	-1.85654
H	-1.43577	-5.2479	0.59787	H	-4.51483	-1.88877	-0.81667
C	-3.73021	-3.13412	2.12153	H	-0.36183	0.53939	-3.34503
H	-4.45639	-3.25618	1.3115	H	1.29171	-3.6935	-3.51979

H	2.72473	1.58072	3.77485
H	4.08468	-2.44809	-0.50743
H	4.35935	-1.3945	1.14682
C	5.06635	1.00233	4.56466
H	4.26865	0.775	5.28072
H	6.02006	0.92874	5.09873
H	4.94945	2.04184	4.23812
C	6.24321	0.3897	2.4419
H	6.32543	-0.29917	1.59441
H	6.12766	1.40246	2.03981
H	7.18867	0.34906	2.99578
C	5.26378	-1.40947	3.92223
H	6.20336	-1.47119	4.48413
H	4.4461	-1.69108	4.59522
H	5.30689	-2.15256	3.11887
C	4.34919	-5.1609	-1.53589
H	5.12988	-5.8364	-1.90536
H	4.73806	-4.65999	-0.6429
H	3.49001	-5.76921	-1.23143
C	3.47595	-4.94091	-3.87298
H	3.19992	-4.27723	-4.70012
H	4.28314	-5.59149	-4.22705
H	2.61564	-5.57919	-3.64188
C	5.19014	-3.31418	-3.04423
H	4.93569	-2.58852	-3.82476
H	5.607	-2.76091	-2.19596
H	5.97774	-3.9703	-3.43311
F	1.17982	2.95727	0.15024
C	2.75562	4.54581	-1.48678
H	3.12598	4.30016	-0.48469
H	3.63537	4.70181	-2.12466
Si	1.81267	6.2062	-1.37893
H	1.5954	6.69646	-2.77521
C	0.13399	6.00289	-0.53732
H	-0.37322	6.96972	-0.43434
H	-0.51616	5.34281	-1.12207
H	0.23714	5.55996	0.4592
C	2.88467	7.47217	-0.46068
H	2.39775	8.45424	-0.42828
H	3.06787	7.15871	0.57454
H	3.85902	7.6012	-0.94712

Thermal correction to Energy= 1.192540

Thermal correction to Enthalpy= 1.193484

Thermal correction to Gibbs Free Energy= 1.019598

Sum of electronic and zero-point Energies= -2887.456784

Sum of electronic and thermal Energies= -2887.389310

Sum of electronic and thermal Enthalpies= -2887.388366

Sum of electronic and thermal Free Energies= -2887.562253

SCF Done: E(wB97XD) = -2888.5871

Int3b′

Number of Negative Frequencies =0

B	1.79407	0.63857	1.11144
B	2.1507	-0.79202	-1.50359
B	1.49006	-1.51832	1.34612
C	-2.05186	-0.94155	1.64586
H	-1.53182	-1.85962	1.88349
C	-3.34926	-0.70201	2.0917
C	-3.99062	0.49715	1.76216
C	-3.24362	1.41723	1.0133
C	-1.94232	1.11828	0.60504
C	-1.11386	2.0579	-0.18581
C	-1.46474	3.39642	-0.36028
C	-0.66328	4.27262	-1.10276
C	0.49257	3.72277	-1.66778
C	0.80817	2.38624	-1.44883
H	1.70618	1.93976	-1.85488
C	3.45831	2.10278	1.74946
C	2.17456	2.33885	2.62615
C	3.98024	-1.7419	-2.54204
C	3.57146	-0.43175	-3.30155
C	1.60704	-2.65636	3.35404
C	2.88128	-2.95018	2.48624
Ir	0.56822	-0.50238	-0.24487
N	-1.36382	-0.06619	0.90554
N	0.0365	1.56467	-0.71754
O	2.95429	1.25817	0.68107
O	1.37838	1.15786	2.33042
O	2.80934	-2.00009	-1.73003
O	2.67152	0.1909	-2.35139
O	0.71798	-2.052	2.38136
O	2.82127	-1.89305	1.49474
H	0.7269	-2.09639	-0.08317
C	-0.70387	-1.00985	-1.90442
C	-0.68502	-0.32958	-3.14145
C	-1.59525	-0.61416	-4.16434
H	-1.54359	-0.06891	-5.10489
C	-2.57028	-1.59922	-3.98601

H	-3.28615	-1.81858	-4.77538
C	-2.63408	-2.31926	-2.78499
C	-1.68452	-1.99191	-1.81044
C	-5.43031	0.82828	2.17431
C	-1.04426	5.75169	-1.21808
C	4.05236	3.3639	1.12908
H	4.37278	4.06775	1.90587
H	4.92912	3.10089	0.52696
H	3.33474	3.86638	0.4752
C	4.54712	1.30001	2.4688
H	5.32763	1.03535	1.74771
H	5.00712	1.87473	3.27973
H	4.14167	0.36903	2.87509
C	2.42038	2.40202	4.12992
H	3.06303	3.25363	4.38119
H	1.46706	2.52992	4.6545
H	2.89031	1.49001	4.50436
C	1.34755	3.55063	2.17981
H	0.38146	3.53149	2.69488
H	1.84942	4.49252	2.42592
H	1.15866	3.52639	1.10416
C	4.20789	-2.85063	3.23037
H	5.03545	-3.04942	2.54043
H	4.35708	-1.85576	3.65704
H	4.25419	-3.58877	4.03934
C	2.79744	-4.27762	1.72252
H	3.60526	-4.31081	0.98449
H	2.90114	-5.13909	2.39103
H	1.84813	-4.36268	1.18324
C	1.85322	-1.61633	4.45184
H	0.89103	-1.31929	4.88232
H	2.48343	-2.01499	5.25417
H	2.31987	-0.71986	4.03713
C	0.92474	-3.89022	3.936
H	1.60263	-4.42743	4.60926
H	0.04321	-3.58914	4.51321
H	0.59503	-4.57573	3.15125
C	2.7652	-0.69874	-4.57829
H	2.35493	0.24831	-4.94552
H	3.38828	-1.13144	-5.36854
H	1.926	-1.3726	-4.38096
C	4.71548	0.53483	-3.59316
H	5.46963	0.0663	-4.23629
H	4.32954	1.41864	-4.11398
H	5.20078	0.87247	-2.6738
C	4.23976	-2.94988	-3.43667
H	5.06951	-2.75297	-4.1257
H	4.50906	-3.81587	-2.82143
H	3.35501	-3.21532	-4.02091
C	5.15725	-1.53059	-1.58077
H	5.25874	-2.41511	-0.94382
H	6.10029	-1.38249	-2.11858
H	4.97824	-0.66794	-0.93208
H	0.06971	0.43487	-3.30677
F	-1.77542	-2.73132	-0.64232
C	-3.69613	-3.35454	-2.51128
H	-3.24452	-4.27847	-2.12579
H	-4.20952	-3.6161	-3.4448
Si	-5.00627	-2.77941	-1.23413
H	-4.8215	-1.31526	-1.01254
C	-4.78086	-3.67558	0.4118
H	-3.76373	-3.52733	0.78937
H	-5.48824	-3.31641	1.16974
H	-4.94154	-4.75532	0.29319
C	-6.74467	-3.07384	-1.91518
H	-6.91724	-4.14149	-2.1052
H	-7.51452	-2.73361	-1.21062
H	-6.89852	-2.54022	-2.86173
H	-2.36372	3.76792	0.11374
H	-3.68926	2.36207	0.7283
H	-3.84123	-1.46995	2.67512
H	1.17614	4.3145	-2.26408
C	-0.08916	6.52454	-2.14369
H	-0.40624	7.57152	-2.20399
H	0.94124	6.51282	-1.77047
H	-0.09162	6.11666	-3.16127
C	-2.47976	5.8788	-1.77608
H	-2.55632	5.42912	-2.77293
H	-3.21861	5.39442	-1.12884
H	-2.75403	6.93726	-1.85717
C	-0.98179	6.37837	0.19547
H	0.02703	6.29749	0.61762
H	-1.24734	7.44129	0.14649
H	-1.67665	5.8895	0.88712
C	-6.26587	1.12656	0.90804
H	-7.30066	1.35755	1.18837
H	-5.87329	1.98375	0.35076
H	-6.27713	0.2641	0.23198

C	-6.0959	-0.33112	2.93611
H	-5.56772	-0.56588	3.86735
H	-7.12286	-0.05444	3.19997
H	-6.14361	-1.24284	2.32902
C	-5.41589	2.07542	3.08787
H	-6.43843	2.32936	3.39223
H	-4.82526	1.89324	3.99342
H	-4.99265	2.94742	2.5771

Thermal correction to Energy= 1.194886

Thermal correction to Enthalpy= 1.195830

Thermal correction to Gibbs Free Energy= 1.023763

Sum of electronic and zero-point Energies= -2887.472345

Sum of electronic and thermal Energies= -2887.404960

Sum of electronic and thermal Enthalpies= -2887.404016

Sum of electronic and thermal Free Energies= -2887.576083

SCF Done: E(wB97XD) = -2888.6126

TS4b′

Number of Negative Frequencies =1

Ir	0.22465	0.06643	-0.08885
B	-1.16297	0.774	-1.55547
B	0.00485	1.69787	1.12969
O	-1.04688	0.26927	-2.86557
O	-1.97155	1.90321	-1.54237
C	0.9264	-3.81068	1.86103
C	0.10959	-2.41325	3.59635
C	0.04568	-1.37594	2.67009
H	-0.27401	-0.37965	2.94968
C	1.92856	-3.95668	-0.9677
N	0.92288	-1.80137	-1.23888
N	0.40063	-1.52393	1.38626
O	0.27823	1.67143	2.51077
O	-0.31566	2.99027	0.74574
C	1.25204	-2.84857	-0.45064
C	0.85603	-2.7343	0.97512
O	3.11197	-0.4555	0.83609
O	2.95112	1.64683	-0.03788
C	2.2742	-4.02693	-2.32397
C	0.55309	-3.6798	3.20473
C	-0.07416	3.90515	1.8317
C	-0.05284	2.95492	3.09381
C	4.36728	1.39965	0.13442
C	4.37268	0.18991	1.13805
C	-1.60069	1.25107	-3.77277
C	-2.59743	2.03517	-2.83922
H	0.51598	1.23363	-1.15553
C	1.8974	-2.93504	-3.11511
C	-0.43702	2.12048	-4.2758
H	0.04465	2.65376	-3.45068
H	0.31349	1.47593	-4.74628
H	-0.76894	2.85409	-5.01893
C	-2.24597	0.5162	-4.94848
H	-2.74569	1.21884	-5.62601
H	-1.47554	-0.01142	-5.52197
H	-2.97707	-0.22151	-4.60945
C	-3.98654	1.38248	-2.7648
H	-4.56665	1.86698	-1.97375
H	-4.53544	1.49661	-3.70646
H	-3.9167	0.3172	-2.52664
C	-2.74079	3.52608	-3.1492
H	-3.14693	3.6831	-4.15593
H	-3.42962	3.98315	-2.43128
H	-1.78336	4.04593	-3.06997
C	-1.18816	4.95532	1.83228
H	-1.07644	5.65646	2.66868
H	-1.14311	5.52948	0.90104
H	-2.1761	4.49254	1.88926
C	1.27496	4.58589	1.55025
H	2.07839	3.8483	1.47535
H	1.21179	5.0993	0.5854
H	1.53198	5.32526	2.31781
C	1.00849	3.29346	4.14357
H	0.82482	4.27963	4.58674
H	0.97845	2.55165	4.94936
H	2.01457	3.28729	3.71754
C	-1.41898	2.81246	3.78262
H	-1.71859	3.74086	4.28183
H	-2.19466	2.52838	3.06921
H	-1.35375	2.02529	4.54244
C	4.92968	1.0417	-1.25068
H	4.6971	1.85453	-1.94592
H	6.01703	0.90586	-1.22968
H	4.47042	0.12624	-1.63735
C	5.03319	2.67667	0.64699
H	4.9429	3.4663	-0.10632
H	4.56462	3.03562	1.56629
H	6.10036	2.51318	0.8404
C	4.31197	0.6225	2.61082

H	4.13179	-0.26097	3.23225
H	5.24686	1.0896	2.94062
H	3.48692	1.32119	2.77848
C	5.50213	-0.82096	0.93759
H	6.48191	-0.35571	1.10013
H	5.39478	-1.6391	1.65814
H	5.48418	-1.25247	-0.06624
C	3.02702	-5.2496	-2.87158
C	0.63923	-4.88191	4.15689
C	1.23356	-1.85433	-2.53915
H	0.91022	-1.00056	-3.12396
B	2.24863	0.48292	0.26385
C	-2.86253	-0.18958	0.66691
C	-1.92059	-0.60944	-0.27776
C	-2.288	-1.76799	-0.99451
C	-3.49025	-2.43768	-0.76434
C	-4.38222	-1.96396	0.19795
C	-4.08478	-0.81393	0.94079
H	-1.62715	-2.12815	-1.7757
H	-3.73626	-3.32739	-1.33955
H	-5.32188	-2.48157	0.3786
C	-5.03724	-0.25539	1.97098
H	-4.48898	0.08944	2.85673
H	-5.71581	-1.04976	2.30855
Si	-6.12509	1.19144	1.34826
H	-6.78614	0.74539	0.08403
C	-5.12403	2.75964	1.01746
H	-5.7533	3.5283	0.55189
H	-4.73644	3.17328	1.95693
H	-4.26457	2.57297	0.36625
C	-7.47432	1.54172	2.63488
H	-7.03906	1.84758	3.59441
H	-8.13563	2.35016	2.30032
H	-8.09737	0.6581	2.8178
F	-2.60533	0.92772	1.40971
H	1.26408	-4.77187	1.49383
H	2.20703	-4.76611	-0.30423
C	3.31174	-5.12632	-4.38039
H	2.38838	-5.04772	-4.9653
H	3.93839	-4.2565	-4.60761
H	3.84633	-6.01744	-4.72707
C	4.37847	-5.39038	-2.13075
H	4.24349	-5.52935	-1.0529
H	4.92828	-6.25878	-2.51246
H	5.00187	-4.5014	-2.27815
C	2.17522	-6.52059	-2.63894
H	1.21262	-6.45049	-3.15803
H	2.70299	-7.40295	-3.01982
H	1.97145	-6.68838	-1.57601
H	2.11313	-2.90211	-4.17614
H	-0.19704	-2.20843	4.61467
C	2.10488	-5.37414	4.2255
H	2.18091	-6.23745	4.89717
H	2.47991	-5.68267	3.24384
H	2.76708	-4.58808	4.60541
C	0.17974	-4.52466	5.58314
H	-0.86527	-4.19548	5.60499
H	0.26121	-5.40704	6.22731
H	0.79829	-3.73552	6.02537
C	-0.26366	-6.02052	3.62417
H	-1.3089	-5.69781	3.56207
H	0.04316	-6.35559	2.62767
H	-0.21339	-6.88568	4.29584

Thermal correction to Energy= 1.193293

Thermal correction to Enthalpy= 1.194237

Thermal correction to Gibbs Free Energy= 1.024617

Sum of electronic and zero-point Energies= -2887.466860

Sum of electronic and thermal Energies= -2887.400188

Sum of electronic and thermal Enthalpies= -2887.399244

Sum of electronic and thermal Free Energies= -2887.568863

SCF Done: E(wB97XD) = -2888.6035

Int5b′

Number of Negative Frequencies =0

B	2.8212	-2.38111	-0.9953
B	3.26104	0.01877	-0.07591
C	-0.94363	-1.05388	-2.82379
H	-0.19852	-0.79607	-3.56848
C	-2.29883	-1.10565	-3.1381
C	-2.71579	-1.70286	-0.87148
C	-1.34317	-1.64683	-0.62128
C	-0.74334	-1.9319	0.70524
C	-1.43824	-2.58111	1.72584
C	0.49806	-2.43782	3.0932
C	1.14642	-1.80234	2.03773
H	2.18195	-1.48708	2.1092
N	-0.46585	-1.30619	-1.59806

N	0.55112	-1.54994	0.86104
O	4.01539	-2.48912	-1.71125
O	2.54715	-3.58589	-0.33245
O	3.99392	-0.48212	1.02451
O	3.81433	1.25312	-0.45499
C	-3.2336	-1.42303	-2.14404
C	-0.83108	-2.85585	2.95878
C	3.72027	-4.42865	-0.4135
C	4.43963	-3.86871	-1.69721
C	4.93287	0.52204	1.47407
C	5.09761	1.4118	0.18972
Ir	1.60623	-0.8093	-0.89879
H	2.26443	-0.36095	-2.29234
C	-1.61023	-3.58146	4.06639
C	-4.74889	-1.46186	-2.39233
C	4.52369	-4.20413	0.87726
H	4.81554	-3.15445	0.97493
H	3.88963	-4.45702	1.73411
H	5.42068	-4.83271	0.9185
C	3.27107	-5.88761	-0.50284
H	4.13055	-6.55765	-0.62716
H	2.75427	-6.17056	0.42076
H	2.58163	-6.04722	-1.33547
C	3.93685	-4.50973	-3.00084
H	4.32097	-3.93188	-3.84755
H	4.27542	-5.54673	-3.10767
H	2.84346	-4.49224	-3.04975
C	5.96779	-3.90931	-1.65617
H	6.33434	-4.94049	-1.58265
H	6.37155	-3.47112	-2.57517
H	6.36012	-3.33846	-0.8111
C	6.15612	0.87167	-0.78602
H	6.07207	1.4181	-1.73094
H	7.17478	1.00074	-0.40206
H	5.98563	-0.18801	-0.99917
C	6.20807	-0.17301	1.95245
H	6.97109	0.55937	2.24315
H	5.98605	-0.79358	2.82776
H	6.62547	-0.82022	1.17758
C	4.27552	1.26948	2.64536
H	3.36059	1.77556	2.32165
H	4.00831	0.54713	3.42437
H	4.94799	2.01338	3.08728
C	5.34188	2.89804	0.45343
H	6.28637	3.05403	0.98889
H	5.40305	3.43372	-0.4999
H	4.53342	3.34445	1.03817
H	-2.45972	-2.89582	1.54805
H	-3.39127	-1.93582	-0.05694
H	-2.60439	-0.88352	-4.15324
H	1.05478	-2.60844	4.00687
C	-2.82687	-2.72005	4.48171
H	-3.49664	-2.52543	3.63743
H	-3.40585	-3.23319	5.25886
H	-2.50448	-1.7521	4.88186
C	-0.74305	-3.83262	5.31461
H	-1.33536	-4.35327	6.07518
H	0.127	-4.4588	5.08691
H	-0.38574	-2.89701	5.75951
C	-2.10535	-4.94838	3.53566
H	-1.26427	-5.57811	3.22522
H	-2.65771	-5.47881	4.32036
H	-2.7751	-4.83747	2.67624
C	-5.44904	-0.46984	-1.43198
H	-6.52929	-0.46893	-1.62104
H	-5.29723	-0.74264	-0.38238
H	-5.07622	0.55125	-1.56511
C	-5.26969	-2.89462	-2.1248
H	-6.35289	-2.93903	-2.28901
H	-4.79424	-3.61934	-2.79553
H	-5.07573	-3.21323	-1.09502
C	-5.10871	-1.07445	-3.83888
H	-4.78117	-0.0571	-4.08056
H	-4.66799	-1.76249	-4.56935
H	-6.19584	-1.11097	-3.96782

Thermal correction to Energy= 0.803489

Thermal correction to Enthalpy= 0.804433

Thermal correction to Gibbs Free Energy= 0.684089

Sum of electronic and zero-point Energies= -1737.030290

Sum of electronic and thermal Energies= -1736.987151

Sum of electronic and thermal Enthalpies= -1736.986207

Sum of electronic and thermal Free Energies= -1737.106552

SCF Done: E(wB97XD) = -1737.6762

Int6b′

Number of Negative Frequencies =0

B	1.18966	-1.93907	-1.06001
B	3.02471	-0.16181	0.01919

C	-1.42937	0.14482	-2.73069	C	-0.78851	-3.90919	-2.40113
H	-0.74048	0.62313	-3.41914	H	-0.91043	-3.5367	-3.42376
C	-2.68111	-0.29425	-3.15571	H	-1.26044	-4.89603	-2.33471
C	-3.05485	-1.08557	-0.9408	H	-1.31616	-3.22428	-1.73093
C	-1.79782	-0.60785	-0.57442	C	1.42248	-4.93746	-2.99847
C	-1.2543	-0.72511	0.80148	H	1.11309	-5.97019	-2.79888
C	-1.95465	-1.32143	1.84952	H	1.17262	-4.70385	-4.03972
C	-0.14505	-0.82774	3.31324	H	2.5083	-4.87172	-2.8915
C	0.51519	-0.26088	2.2273	C	5.77984	1.25693	0.58495
H	1.51112	0.15636	2.32736	H	5.53903	2.29593	0.83251
N	-0.98084	-0.0159	-1.48081	H	6.83584	1.0848	0.82071
N	0.00027	-0.23517	0.98855	H	5.63679	1.12288	-0.49183
O	1.23902	-2.63463	-2.27653	C	6.29539	-1.61237	0.3702
O	0.92589	-2.84138	-0.01762	H	7.05567	-1.54509	1.1576
O	3.98704	-1.16425	-0.20498	H	6.24111	-2.65652	0.04149
O	3.49405	0.67426	1.06084	H	6.62084	-1.01052	-0.48223
C	-3.52558	-0.95259	-2.25501	C	4.41548	-2.16177	1.93556
C	-1.40439	-1.41159	3.1361	H	3.43249	-1.85947	2.31165
C	0.98119	-4.18923	-0.53412	H	4.3057	-3.14382	1.46629
C	0.70964	-3.96453	-2.06416	H	5.10282	-2.25839	2.78342
C	4.93192	-1.16424	0.88879	C	5.08354	0.52216	2.87755
C	4.86166	0.32085	1.38135	H	6.08086	0.17762	3.17547
Ir	1.19227	0.04589	-0.82103	H	5.00624	1.58728	3.12481
C	2.12239	3.5341	-1.04808	H	4.33876	-0.01448	3.47112
C	1.29025	4.69843	-1.67229	C	3.11363	2.89554	-2.00588
C	-2.96759	3.18166	2.19081	H	3.80919	3.66395	-2.36439
C	-3.69104	2.5899	0.92564	H	3.68737	2.12115	-1.49452
B	-0.0821	3.34135	-0.43673	H	2.61882	2.43723	-2.86296
B	-1.48842	2.98186	0.44681	C	2.81328	3.90441	0.26691
O	-2.569	2.22864	0.0586	H	3.13979	2.97898	0.74776
O	-1.69587	3.63744	1.63767	H	3.68434	4.5443	0.0889
O	0.04348	4.62179	-0.91492	H	2.12893	4.42757	0.94377
O	1.04895	2.58003	-0.67461	C	1.89417	6.08417	-1.48062
H	1.95006	0.14271	-2.24397	H	2.87726	6.14178	-1.96113
C	-2.17501	-2.12148	4.25543	H	1.2465	6.83856	-1.94029
C	-4.88354	-1.54685	-2.6479	H	2.00633	6.33262	-0.42249
C	2.38635	-4.73134	-0.2472	C	0.9284	4.46404	-3.14248
H	3.14565	-4.11575	-0.7384	H	0.16867	5.19306	-3.44285
H	2.56766	-4.69327	0.83193	H	1.79919	4.58624	-3.79413
H	2.49942	-5.77001	-0.57765	H	0.51806	3.45991	-3.29195
C	-0.06646	-5.02994	0.19192	C	-4.53129	1.34718	1.19289
H	-0.12514	-6.04007	-0.23031	H	-5.34273	1.57944	1.89196
H	0.20204	-5.12134	1.2507	H	-4.97817	0.99449	0.25899
H	-1.05651	-4.56934	0.13832	H	-3.93076	0.53814	1.61132

C	-4.512	3.62347	0.14742
H	-4.81783	3.18552	-0.80859
H	-5.41288	3.91842	0.69539
H	-3.92152	4.52058	-0.06574
C	-2.6315	2.12755	3.24764
H	-1.97692	2.57332	4.00392
H	-3.53411	1.75956	3.74557
H	-2.10572	1.27987	2.80325
C	-3.66909	4.3733	2.8345
H	-4.66227	4.08537	3.19739
H	-3.08402	4.72579	3.6907
H	-3.78039	5.20557	2.13532
H	-2.9369	-1.73697	1.66331
H	-3.67328	-1.58018	-0.20352
H	0.35247	-0.8221	4.27518
H	-2.96134	-0.13321	-4.18939
C	-4.82875	-3.07772	-2.4325
H	-4.63193	-3.33507	-1.38597
H	-5.78601	-3.53177	-2.71605
H	-4.04124	-3.53187	-3.04552
C	-5.23164	-1.27222	-4.12117
H	-6.20886	-1.70983	-4.35416
H	-5.28937	-0.19805	-4.33224
H	-4.49853	-1.71832	-4.80293
C	-5.99343	-0.93976	-1.75998
H	-6.0433	0.14851	-1.88307
H	-6.96754	-1.35813	-2.04041
H	-5.83387	-1.1547	-0.69806
C	-2.42571	-3.5881	3.83352
H	-2.97237	-4.11773	4.62306
H	-3.01876	-3.65352	2.91463
H	-1.47929	-4.11411	3.66165
C	-1.39745	-2.11901	5.58302
H	-1.2091	-1.10055	5.94207
H	-1.98108	-2.63829	6.35143
H	-0.4349	-2.63533	5.49177
C	-3.52924	-1.4113	4.48146
H	-3.37889	-0.3749	4.80281
H	-4.14542	-1.40049	3.5758
H	-4.09662	-1.93027	5.26349

Thermal correction to Energy= 1.191311

Thermal correction to Enthalpy= 1.192255

Thermal correction to Gibbs Free Energy= 1.020822

Sum of electronic and zero-point Energies= -2559.203803

Sum of electronic and thermal Energies= -2559.138438

Sum of electronic and thermal Enthalpies= -2559.137494

Sum of electronic and thermal Free Energies= -2559.308927

SCF Done: E(wB97XD) = -2560.3226

TS7b′

Number of Negative Frequencies =1

Ir	-0.51239	0.16143	-0.24028
B	-1.5773	-2.30175	-0.25285
B	-0.42888	-2.35089	1.063
H	0.51117	-1.62505	-2.7027
C	2.66961	-1.6394	-2.90775
C	3.77659	-0.33847	-1.25193
C	2.53338	0.0199	-0.72581
C	2.37366	0.89205	0.46603
C	3.43967	1.58176	1.04724
C	1.95602	2.4505	2.68503
C	0.92601	1.75398	2.05879
H	-0.09159	1.78376	2.43181
B	-2.1258	0.67479	0.89366
N	1.38468	-0.43814	-1.27677
N	1.11431	0.99325	0.96839
O	-2.0651	0.6888	2.30331
O	-3.41256	1.03363	0.49033
O	-1.31639	-2.96663	-1.4485
O	-2.92788	-2.33839	0.06001
O	-0.42104	3.1535	-0.67812
O	-1.45032	2.06556	-2.4102
C	3.87649	-1.17852	-2.37039
C	3.25867	2.38923	2.17852
C	-4.2085	1.39729	1.63735
C	-3.40991	0.75026	2.83347
C	-1.18575	3.40079	-2.89054
C	-0.92136	4.19226	-1.55518
C	-2.58648	-3.24037	-2.10228
C	-3.6109	-3.17753	-0.90073
H	-1.56441	-0.31765	-1.33514
B	-0.85566	1.91402	-1.15969
C	0.25046	-2.84825	3.20442
C	1.46035	-1.25505	-2.33295
O	0.94554	-2.45346	0.97445
O	-0.88794	-2.67743	2.31742
C	1.43323	-3.0926	2.18997
C	5.25453	-1.55982	-2.93381

C	4.44512	3.14824	2.79236	H	-4.84176	-1.50769	-1.5776
C	-2.39183	3.87401	-3.7035	C	-3.83377	-4.53432	-0.21336
H	-2.26369	4.91143	-4.03582	H	-4.40242	-4.37375	0.70795
H	-2.50289	3.24518	-4.59345	H	-4.3952	-5.22774	-0.84969
H	-3.31674	3.8015	-3.12684	H	-2.88237	-5.00171	0.05917
C	0.05176	3.3158	-3.80016	C	1.64557	-4.57275	1.83897
H	-0.14208	2.58358	-4.59042	H	2.34021	-4.64157	0.99587
H	0.28448	4.27819	-4.27039	H	2.06699	-5.13286	2.68093
H	0.92936	2.98026	-3.23812	H	0.7068	-5.04817	1.5389
C	-2.20596	4.74512	-0.92206	C	2.76726	-2.45444	2.57451
H	-1.97826	5.10849	0.08541	H	3.15061	-2.88291	3.50817
H	-2.62132	5.5776	-1.50152	H	3.50483	-2.6452	1.78758
H	-2.96512	3.96304	-0.83361	H	2.67815	-1.37313	2.69908
C	0.12965	5.29919	-1.64711	C	0.38672	-1.55048	4.01119
H	-0.18353	6.08319	-2.34727	H	-0.54738	-1.36954	4.55081
H	0.26264	5.76076	-0.66259	H	1.20257	-1.61371	4.74049
H	1.09883	4.91119	-1.9694	H	0.55666	-0.69268	3.35688
C	-3.38873	1.57436	4.12273	C	-0.04862	-4.01586	4.14528
H	-4.39771	1.68608	4.53778	H	0.80475	-4.21903	4.80326
H	-2.77271	1.06508	4.87217	H	-0.90838	-3.76422	4.77436
H	-2.96867	2.57078	3.96155	H	-0.29141	-4.92856	3.5963
C	-3.84132	-0.69096	3.14695	H	4.68002	0.02585	-0.77767
H	-3.13187	-1.12605	3.85784	H	4.42395	1.50241	0.60163
H	-4.84234	-0.72541	3.59344	H	2.64629	-2.3025	-3.76381
H	-3.82434	-1.31274	2.24831	H	1.71523	3.04112	3.56046
C	-5.62372	0.85051	1.43487	C	4.02956	3.97116	4.02654
H	-6.26222	1.07059	2.29939	H	4.90288	4.49561	4.42955
H	-6.07367	1.31969	0.5532	H	3.27624	4.72682	3.77713
H	-5.61308	-0.23027	1.27336	H	3.6283	3.33687	4.82506
C	-4.25303	2.9318	1.69652	C	5.03279	4.11648	1.73738
H	-3.24942	3.3506	1.81804	H	5.39461	3.58568	0.85053
H	-4.66275	3.31089	0.75484	H	4.2813	4.84325	1.40969
H	-4.8855	3.29332	2.51571	H	5.87913	4.6687	2.16291
C	-2.47213	-4.60357	-2.78769	C	5.53312	2.13614	3.22494
H	-3.42315	-4.89295	-3.25023	H	6.39029	2.66605	3.6571
H	-1.7153	-4.5532	-3.57783	H	5.14778	1.44103	3.97951
H	-2.17327	-5.38559	-2.08551	H	5.90042	1.54364	2.38012
C	-2.82488	-2.15139	-3.15866	C	6.06619	-2.29678	-1.84149
H	-1.97047	-2.12533	-3.84374	H	7.05316	-2.57635	-2.22872
H	-3.72428	-2.36459	-3.74745	H	6.22108	-1.67232	-0.95521
H	-2.92737	-1.16156	-2.70761	H	5.55429	-3.21157	-1.52223
C	-4.95986	-2.53765	-1.23352	C	6.00673	-0.27525	-3.35719
H	-5.48785	-3.11243	-2.00418	H	6.99523	-0.53121	-3.75678
H	-5.58815	-2.52173	-0.33657	H	5.45569	0.26583	-4.13462

H	6.15539	0.40833	-2.51449
C	5.14367	-2.48369	-4.1617
H	4.6468	-3.42943	-3.91786
H	4.59407	-2.00819	-4.9819
H	6.14641	-2.72401	-4.53207

Thermal correction to Energy= 1.190452

Thermal correction to Enthalpy= 1.191396

Thermal correction to Gibbs Free Energy= 1.027370

Sum of electronic and zero-point Energies= -2559.195837

Sum of electronic and thermal Energies= -2559.131839

Sum of electronic and thermal Enthalpies= -2559.130895

Sum of electronic and thermal Free Energies= -2559.294921

SCF Done: E(wB97XD) = -2560.3133

Int8b′

Number of Negative Frequencies =0

Ir	0.59654	0.12597	-0.51077
B	0.14901	1.85878	-1.648
H	1.53451	-0.14601	-1.7903
C	-1.44547	-1.71366	-2.19274
C	-2.65454	-2.31686	-2.52675
C	-3.6576	-1.10614	-0.73787
C	-2.41786	-0.52913	-0.45659
C	-2.20616	0.4422	0.6419
C	-3.24342	0.90106	1.45477
C	-1.68909	2.24054	2.65556
C	-0.69264	1.75982	1.81365
B	0.70694	-1.39673	1.11897
N	-1.3215	-0.84179	-1.18623
N	-0.9312	0.8767	0.83191
O	1.58814	-1.50929	2.17514
O	-0.4793	-2.06432	1.39562
O	3.30575	1.60802	-0.6021
O	2.40432	1.57596	1.49571
O	1.38411	-2.80351	-1.26062
O	3.19459	-1.64088	-0.47223
C	-3.80693	-2.02157	-1.78788
C	-3.01108	1.81141	2.49399
C	-0.34329	-2.78667	2.64579
C	0.88468	-2.06486	3.31781
C	3.68553	-2.95677	-0.81214
C	2.55609	-3.49471	-1.76252
C	4.17692	2.41714	0.22114
C	3.79294	1.94536	1.66585
C	-0.58353	3.13795	-3.4285
B	1.81037	-1.61564	-0.64965
H	0.34036	2.06234	1.92613
H	-0.53077	-1.94434	-2.72525
B	2.21678	1.17535	0.16247
C	-0.42862	4.04534	-2.15925
O	-0.29716	3.06239	-1.0991
O	0.14582	1.94376	-3.04136
C	-5.17649	-2.65468	-2.0649
C	-4.16986	2.27943	3.38219
C	0.86049	4.87429	-2.16984
H	1.00226	5.32912	-1.18394
H	1.72864	4.24217	-2.37791
H	0.82107	5.67574	-2.91572
C	-1.62707	4.93833	-1.85164
H	-1.43021	5.52355	-0.94615
H	-1.81415	5.6393	-2.67356
H	-2.53323	4.35101	-1.68136
C	-2.03419	2.70797	-3.68774
H	-2.64909	3.54049	-4.04694
H	-2.03979	1.92167	-4.45009
H	-2.49188	2.3011	-2.77992
C	0.03063	3.70204	-4.70591
H	-0.11828	2.99719	-5.53168
H	-0.44527	4.65076	-4.98055
H	1.10503	3.86918	-4.59623
C	5.62349	2.1464	-0.18346
H	5.79525	2.50326	-1.20554
H	6.32234	2.67016	0.47974
H	5.85006	1.07774	-0.15862
C	3.82136	3.88592	-0.04101
H	3.92737	4.09379	-1.11091
H	2.78466	4.09107	0.24219
H	4.47893	4.56948	0.50799
C	4.5421	0.67602	2.09597
H	5.60318	0.87584	2.28466
H	4.09263	0.29378	3.01791
H	4.44778	-0.10238	1.333
C	3.8912	3.01392	2.7497
H	3.58916	2.59021	3.71447
H	4.92037	3.37866	2.84871
H	3.23876	3.86518	2.53752
C	5.05847	-2.81151	-1.46247
H	5.77354	-2.42032	-0.72999

H	5.42942	-3.78113	-1.81512
H	5.03111	-2.11753	-2.30616
C	2.7407	-3.05727	-3.22141
H	3.58502	-3.56797	-3.69692
H	1.83258	-3.3007	-3.78379
H	2.90082	-1.97628	-3.29036
C	2.30652	-4.99739	-1.68898
H	1.49864	-5.27325	-2.3762
H	3.20399	-5.55605	-1.97903
H	2.01143	-5.30734	-0.68312
C	3.80222	-3.75058	0.49454
H	4.45301	-3.20429	1.18473
H	2.82563	-3.85368	0.97321
H	4.22693	-4.74701	0.33072
C	1.84327	-2.98169	4.07214
H	2.66909	-2.39046	4.48299
H	1.33504	-3.48118	4.90494
H	2.27177	-3.74292	3.41582
C	-0.07403	-4.25164	2.28515
H	0.01709	-4.87958	3.17788
H	-0.90597	-4.62868	1.68073
H	0.83926	-4.35041	1.69162
C	-1.65939	-2.67282	3.41188
H	-2.45558	-3.17126	2.84765
H	-1.5854	-3.15634	4.39277
H	-1.95475	-1.63074	3.55721
C	0.48088	-0.88168	4.20454
H	-0.01103	-1.21658	5.12419
H	1.37928	-0.31816	4.47664
H	-0.19177	-0.20162	3.67441
H	-1.40506	2.94214	3.42999
H	-2.67196	-3.01261	-3.35645
H	-4.51641	-0.85208	-0.13005
H	-4.24977	0.5439	1.27719
C	-5.61005	-3.46593	-0.82207
H	-5.6947	-2.83356	0.06856
H	-6.58835	-3.92955	-0.99771
H	-4.8896	-4.2629	-0.60333
C	-5.14402	-3.59886	-3.27979
H	-6.14261	-4.01945	-3.44336
H	-4.85062	-3.07327	-4.19599
H	-4.45353	-4.43636	-3.12705
C	-6.21159	-1.53902	-2.33763
H	-5.92637	-0.94078	-3.21111
H	-7.19521	-1.9819	-2.53472
H	-6.31779	-0.86074	-1.48396
C	-4.80503	1.04752	4.06794
H	-5.62937	1.36307	4.71883
H	-5.20897	0.33701	3.33862
H	-4.06862	0.51753	4.68385
C	-3.70384	3.26022	4.47201
H	-3.26384	4.16781	4.04285
H	-4.56148	3.56415	5.08258
H	-2.96606	2.80336	5.14183
C	-5.22822	2.98494	2.50307
H	-4.79837	3.85404	1.99128
H	-5.64012	2.31352	1.74172
H	-6.06148	3.33373	3.12499

Thermal correction to Energy= 1.189911

Thermal correction to Enthalpy= 1.190856

Thermal correction to Gibbs Free Energy= 1.024789

Sum of electronic and zero-point Energies= -2559.206665

Sum of electronic and thermal Energies= -2559.141769

Sum of electronic and thermal Enthalpies= -2559.140825

Sum of electronic and thermal Free Energies= -2559.306892

SCF Done: E(wB97XD) = -2560.3336

TS9b′

Number of Negative Frequencies =1

Ir	0.30432	-0.39041	-0.29279
B	1.59617	-0.44902	1.31485
B	-0.78153	-1.28543	1.30941
O	2.79565	-1.14439	1.40356
O	1.38944	0.30282	2.47394
C	-2.36012	3.17527	0.31951
C	-0.3243	3.79927	1.37059
C	0.18961	2.56112	0.99993
H	1.20152	2.28767	1.24802
C	-3.86749	1.03405	-1.11775
N	-1.7738	-0.12647	-1.23812
N	-0.52032	1.63885	0.3363
O	-2.01438	-0.73003	1.66093
O	-0.4942	-2.3587	2.14202
C	-2.51072	0.92529	-0.80982
C	-1.7901	1.94579	-0.01659
O	0.03747	-2.83679	-2.0769
O	1.19429	-3.37749	-0.18612
C	-4.51514	0.07212	-1.90214

C	-1.64013	4.13693	1.03945	H	2.77058	-3.54189	-2.33297
C	-1.65391	-2.63715	2.96918	C	1.18919	-5.7934	-0.2177
C	-2.4096	-1.26465	2.94945	H	2.05332	-5.86583	0.45195
C	1.2994	-4.52661	-1.06021	H	0.28659	-5.78874	0.39861
C	0.11432	-4.28933	-2.05834	H	1.17646	-6.68635	-0.85354
C	3.28924	-1.04949	2.76108	C	-1.23019	-4.80171	-1.5281
C	2.60951	0.27322	3.26144	H	-2.0367	-4.42946	-2.16929
H	1.73472	-0.98167	-0.56293	H	-1.27332	-5.89597	-1.52651
C	-3.71728	-0.97623	-2.37208	H	-1.41225	-4.44256	-0.51054
C	2.79683	-2.29688	3.504	C	0.35017	-4.78562	-3.48004
H	1.70455	-2.34466	3.4928	H	0.51059	-5.86987	-3.48822
H	3.17375	-3.18602	2.98758	H	-0.52582	-4.56794	-4.10156
H	3.15429	-2.32195	4.53945	H	1.2161	-4.30053	-3.93721
C	4.8138	-1.01528	2.72243	C	-6.01527	0.19724	-2.19052
H	5.22575	-0.83775	3.72275	C	-2.29009	5.47624	1.4057
H	5.19564	-1.97836	2.3657	C	-2.37332	-1.03935	-2.01667
H	5.18159	-0.23912	2.04609	H	-1.74302	-1.85452	-2.34795
C	3.40797	1.53307	2.9036	B	0.54165	-2.36061	-0.86253
H	2.7976	2.41684	3.11995	C	2.22423	1.59064	-1.79486
H	4.33101	1.60654	3.48849	C	1.14739	0.72851	-1.97157
H	3.66198	1.5461	1.83932	C	0.69791	0.62108	-3.30253
C	2.23273	0.28693	4.73889	C	1.30144	1.32938	-4.34925
H	3.12427	0.17754	5.36711	C	2.39036	2.16678	-4.09753
H	1.75292	1.23895	4.99235	C	2.88387	2.31696	-2.79209
H	1.53464	-0.51777	4.98233	H	-0.13708	-0.03992	-3.52576
C	-1.17762	-3.09454	4.3442	H	0.92686	1.22325	-5.36568
H	-2.02723	-3.24089	5.02139	H	2.86755	2.71136	-4.90959
H	-0.64669	-4.04881	4.25507	H	-3.37192	3.39602	0.00552
H	-0.4943	-2.3701	4.79423	H	-4.43489	1.86914	-0.72837
C	-2.43268	-3.76131	2.27529	H	-4.11633	-1.76305	-3.00083
H	-2.78695	-3.44011	1.2904	H	0.32196	4.47893	1.91219
H	-1.76766	-4.61971	2.13413	C	-1.35136	6.36432	2.24072
H	-3.29476	-4.08676	2.86796	H	-1.86298	7.29874	2.49731
C	-3.93143	-1.35603	2.99601	H	-1.05902	5.8771	3.17822
H	-4.26619	-1.84076	3.92054	H	-0.44076	6.62699	1.68998
H	-4.36329	-0.34921	2.96549	C	-2.65447	6.22979	0.10527
H	-4.32751	-1.91586	2.14458	H	-1.76002	6.42505	-0.49788
C	-1.89732	-0.2802	4.00826	H	-3.36082	5.66365	-0.51178
H	-2.18607	-0.58034	5.02152	H	-3.11946	7.19287	0.34821
H	-0.80816	-0.19026	3.95506	C	-3.57352	5.20965	2.22582
H	-2.32536	0.70833	3.80768	H	-3.34482	4.66676	3.15049
C	2.67525	-4.4535	-1.73367	H	-4.04798	6.16004	2.49788
H	3.44737	-4.4296	-0.95769	H	-4.30616	4.62187	1.66216
H	2.8605	-5.31904	-2.37907	C	-6.29288	1.55014	-2.88473

H	-7.36402	1.64757	-3.09808
H	-6.00065	2.39982	-2.25792
H	-5.74783	1.62546	-3.83286
C	-6.52551	-0.93471	-3.09892
H	-6.38522	-1.92015	-2.63986
H	-7.59819	-0.80417	-3.28056
H	-6.021	-0.93288	-4.07209
C	-6.78433	0.14002	-0.84963
H	-6.59614	-0.80557	-0.32727
H	-6.49801	0.95836	-0.17995
H	-7.86272	0.21922	-1.03296
F	2.72791	1.76169	-0.51445
C	4.08764	3.16616	-2.47037
H	3.92853	3.73109	-1.54258
H	4.25315	3.9026	-3.26731
Si	5.69666	2.15882	-2.26691
H	5.91782	1.41121	-3.54427
C	5.58635	0.9238	-0.84438
H	4.73436	0.24606	-0.95677
H	6.49982	0.31823	-0.7821
H	5.46801	1.44939	0.1108
C	7.14058	3.34834	-1.99435
H	7.00677	3.91349	-1.06246
H	8.0944	2.81049	-1.92075
H	7.22666	4.07327	-2.81355

Thermal correction to Energy= 1.193247

Thermal correction to Enthalpy= 1.194192

Thermal correction to Gibbs Free Energy= 1.021690

Sum of electronic and zero-point Energies= -2887.457904

Sum of electronic and thermal Energies= -2887.390657

Sum of electronic and thermal Enthalpies= -2887.389713

Sum of electronic and thermal Free Energies= -2887.562215

SCF Done: E(wB97XD) = -2559.02

TS2a-1

Number of Negative Frequencies = 1

Co	-0.58871	0.13203	0.10985
P	-0.54789	2.40451	0.09733
P	-0.89891	-2.03002	-0.56932
N	-2.5468	0.36381	-0.38549
C	-2.37918	2.73463	0.20801
H	-2.59683	2.78701	1.28496
H	-2.69306	3.69105	-0.22526
C	-3.16373	1.5847	-0.36591
C	-4.48282	1.76492	-0.77396
H	-4.90383	2.76707	-0.758
C	-5.2643	0.68105	-1.18389
C	-4.66378	-0.57887	-1.08764
H	-5.23175	-1.47516	-1.32367
C	-3.34278	-0.71254	-0.6709
C	-2.75558	-2.07437	-0.41695
C	4.08531	0.79439	-1.53482
C	3.98949	-0.7852	-1.60723
B	1.9114	0.12817	-1.25878
O	2.77096	1.17569	-1.03315
O	2.54901	-1.01117	-1.68408
H	0.71461	0.24936	-1.30956
C	-0.05816	3.15702	-1.57718
H	0.94992	2.76191	-1.74758
C	0.00488	4.68983	-1.67263
H	0.25097	4.97773	-2.70361
H	-0.95795	5.1558	-1.43034
H	0.7678	5.12431	-1.02298
C	-0.97985	2.62278	-2.68789
H	-1.12777	1.54443	-2.61544
H	-1.96673	3.09924	-2.64742
H	-0.54962	2.84852	-3.672
C	0.15489	3.52904	1.43712
H	-0.08848	2.95249	2.33252
C	-0.46589	4.9244	1.63019
H	-1.55742	4.88778	1.71381
H	-0.08653	5.35524	2.56618
H	-0.21304	5.62112	0.8278
C	1.68954	3.58756	1.35351
H	2.0332	4.17829	0.49691
H	2.09377	4.05561	2.25997
H	2.12278	2.58787	1.25993
H	-3.24386	-2.8437	-1.02404
H	-2.95006	-2.32567	0.6346
C	-0.5798	-2.42471	-2.39965
H	0.51448	-2.45498	-2.46904
C	-1.13957	-3.76934	-2.89628
H	-2.23175	-3.81439	-2.80789
H	-0.89991	-3.89156	-3.96072
H	-0.72243	-4.62929	-2.36896
C	-1.07289	-1.2987	-3.32215
H	-2.16043	-1.17151	-3.2652
H	-0.60746	-0.34435	-3.07575

H	-0.8238	-1.53878	-4.36412
C	-0.30701	-3.57047	0.35321
H	-0.15725	-3.18777	1.36345
C	1.07695	-4.03036	-0.14207
H	1.52424	-4.68803	0.61184
H	1.01683	-4.58974	-1.08221
H	1.7562	-3.18686	-0.29891
C	-1.2844	-4.75228	0.47378
H	-1.50624	-5.22526	-0.48767
H	-0.83814	-5.51564	1.12363
H	-2.23477	-4.45915	0.93173
C	4.49706	-1.49174	-0.34545
H	4.0201	-1.10005	0.55618
H	4.25953	-2.55804	-0.41185
H	5.58275	-1.39124	-0.24124
C	4.62835	-1.4164	-2.84498
H	5.70968	-1.23789	-2.86159
H	4.46607	-2.49884	-2.82515
H	4.19634	-1.02835	-3.77025
C	4.23514	1.46586	-2.90729
H	4.11801	2.54764	-2.78788
H	5.21962	1.27411	-3.34691
H	3.46882	1.11993	-3.60822
C	5.14176	1.33663	-0.57293
H	6.14876	1.04497	-0.89264
H	5.09493	2.43032	-0.55836
H	4.98221	0.97876	0.4465
C	-6.67444	0.85551	-1.68612
H	-6.69651	0.94461	-2.78114
H	-7.30415	-0.00022	-1.41904
H	-7.13695	1.76008	-1.27718
C	0.41951	0.9578	3.66977
Si	0.17683	-0.37681	2.28313
C	-1.20844	-1.39789	3.18565
H	-0.89399	-1.69921	4.19433
H	-2.08674	-0.74687	3.29802
H	-1.54673	-2.30153	2.66686
C	1.8619	-1.38917	2.5472
H	2.64988	-0.64809	2.35664
H	1.94366	-2.14527	1.76239
C	2.08924	-2.01074	3.8915
C	2.74544	-1.34222	4.94143
C	1.63894	-3.30137	4.19661
C	2.92276	-1.92191	6.199
C	1.80007	-3.91071	5.43284
C	2.44902	-3.21041	6.45194
H	3.43658	-1.36674	6.9795
H	1.42146	-4.9178	5.57816
H	2.58373	-3.67096	7.42657
F	1.01196	-4.01939	3.21907
H	0.62632	0.45562	4.62421
H	1.251	1.64807	3.48105
H	-0.48926	1.55547	3.82293
H	3.12697	-0.3415	4.75408

Thermal correction to Energy= 0.972190

Thermal correction to Enthalpy=0.973134

Thermal correction to Gibbs Free Energy= 0.829347

Sum of electronic and zero-point Energies=-2817.427165

Sum of electronic and thermal Energies=-2817.372369

Sum of electronic and thermal Enthalpies=-2817.371425

Sum of electronic and thermal Free Energies=-2817.515211

SCF Done: E(wB97XD) = -2819.35443705

TS12a-1

Number of Negative Frequencies =1

Co	0.76808	-1.54896	-1.87913
P	0.64463	-3.8071	-2.43409
P	1.98838	0.31154	-1.50714
N	2.52419	-2.34998	-0.72042
C	2.44407	-4.26215	-2.1935
H	2.62502	-5.33896	-2.15869
H	2.95016	-3.87604	-3.08941
C	3.04521	-3.55894	-1.00674
C	4.15362	-4.06266	-0.32209
H	4.53417	-5.04984	-0.57068
C	4.7886	-3.28468	0.6516
C	4.30681	-1.98466	0.84319
H	4.80455	-1.31118	1.53568
C	3.18761	-1.54459	0.13531
C	2.69072	-0.12597	0.19108
C	0.33628	-4.44614	-4.1862
H	-0.75315	-4.56514	-4.23353
C	0.98754	-5.8037	-4.50747
H	0.63311	-6.16482	-5.48149
H	2.07777	-5.7134	-4.57631
H	0.76237	-6.5777	-3.7671
C	0.72525	-3.39207	-5.23352

H	0.20805	-2.45106	-5.04115
H	1.80432	-3.1958	-5.22819
H	0.45935	-3.75411	-6.23595
C	-0.25911	-5.05211	-1.27889
H	-0.68132	-4.38284	-0.52232
C	0.6491	-6.05638	-0.54972
H	1.45711	-5.56864	0.00402
H	0.05186	-6.62164	0.1775
H	1.09556	-6.78605	-1.2362
C	-1.43585	-5.80334	-1.92743
H	-1.0956	-6.56787	-2.63387
H	-2.01136	-6.31507	-1.14568
H	-2.12687	-5.14223	-2.4557
H	3.47744	0.54886	0.54101
H	1.86039	-0.04128	0.8973
C	3.57383	0.32997	-2.55201
H	3.91597	-0.70275	-2.408
C	3.31772	0.49818	-4.06033
H	3.07632	1.5316	-4.32492
H	4.22345	0.22223	-4.61621
H	2.50193	-0.14814	-4.39498
C	4.69838	1.26858	-2.08216
H	4.44647	2.32244	-2.24002
H	4.95133	1.1356	-1.0252
H	5.60893	1.06537	-2.66117
C	1.4145	2.10705	-1.28983
H	0.36625	1.97934	-1.0077
C	1.40958	2.88065	-2.61747
H	0.84894	3.81509	-2.48972
H	2.42122	3.14848	-2.93942
H	0.9234	2.3109	-3.41119
C	2.12827	2.91833	-0.19554
H	3.19361	3.05785	-0.40707
H	1.67574	3.91692	-0.13421
H	2.03816	2.46107	0.79399
C	5.95495	-3.81599	1.44607
H	6.63225	-3.01205	1.75205
H	5.60501	-4.31395	2.36008
H	6.52938	-4.55181	0.87383
C	-1.05179	0.3145	0.86176
C	-2.11703	-0.10351	-4.44354
C	-2.22212	1.03729	-3.36627
B	-0.85176	-0.668	-2.54618
O	-1.53047	-1.18141	-3.69797

O	-1.10662	0.74055	-2.50242
C	-2.0749	2.45932	-3.9137
H	-2.89545	2.69832	-4.6011
H	-2.11083	3.17899	-3.08817
H	-1.13101	2.60156	-4.44398
C	-1.16348	0.24868	-5.59899
H	-1.00167	-0.64472	-6.21096
H	-1.57181	1.03338	-6.24643
H	-0.19057	0.5758	-5.21976
C	-3.51214	0.9607	-2.52886
H	-3.42331	1.65023	-1.68248
H	-4.39314	1.25078	-3.113
H	-3.67462	-0.04321	-2.12881
C	-3.45672	-0.57615	-5.01712
H	-3.97179	0.23376	-5.54859
H	-3.28353	-1.38866	-5.73164
H	-4.11718	-0.95449	-4.2332
C	-1.42348	-1.49021	-0.99047
C	-2.26401	-2.5484	-1.40803
C	-1.78489	-0.85819	0.23816
C	-3.39305	-2.9836	-0.71901
H	-2.06141	-2.99286	-2.37406
C	-2.94478	-1.3023	0.88566
C	-3.75199	-2.34367	0.46372
H	-4.0011	-3.79613	-1.10933
H	-4.63117	-2.6114	1.04091
H	-1.57519	1.24617	0.5936
F	-3.33601	-0.6566	2.02814
H	-0.07401	0.38377	0.38799
Si	-0.85053	0.35984	2.78001
H	-1.02848	-1.00069	3.36241
C	0.89497	0.91632	3.29585
H	1.18439	1.87063	2.8391
H	0.91311	1.06096	4.38385
H	1.66702	0.17623	3.05871
C	-2.04628	1.6051	3.56065
H	-1.79619	2.627	3.24713
H	-3.0818	1.40594	3.27329
H	-1.9831	1.57459	4.65525
H	1.54024	-1.56879	-3.15848

Thermal correction to Energy=0.966994

Thermal correction to Enthalpy=0.967938

Thermal correction to Gibbs Free Energy=0.828604

Sum of electronic and zero-point Energies=-2817.390012

Sum of electronic and thermal Energies=-2817.336119

Sum of electronic and thermal Enthalpies=-2817.335175

Sum of electronic and thermal Free Energies=-2817.474509

SCF Done: E(wB97XD) = -2819.31658178

Int11a-1

Number of Negative Frequencies =0

Co	0.58749	-1.05687	-1.73133
P	0.3608	-3.30283	-1.81143
P	1.43715	1.0627	-1.58291
N	2.33614	-1.54223	-0.52364
C	1.7742	-3.89381	-0.72357
H	1.34583	-4.1744	0.24614
H	2.2505	-4.79721	-1.11809
C	2.78897	-2.80875	-0.46095
C	4.09737	-3.09869	-0.06638
H	4.434	-4.13168	-0.04365
C	4.96087	-2.06711	0.31345
C	4.44158	-0.76695	0.32
H	5.05337	0.06786	0.65071
C	3.1287	-0.53971	-0.09066
C	2.47372	0.81284	-0.04321
C	0.6949	-4.23467	-3.42275
H	-0.03471	-3.79726	-4.10798
C	0.49112	-5.75736	-3.38319
H	0.79639	-6.18834	-4.34585
H	1.09622	-6.24427	-2.60826
H	-0.55347	-6.03353	-3.22145
C	2.10028	-3.90282	-3.95473
H	2.2761	-2.82485	-3.9867
H	2.88665	-4.35981	-3.34031
H	2.21213	-4.30342	-4.97039
C	-1.16216	-4.03943	-0.97561
H	-1.25027	-3.366	-0.11356
C	-1.09941	-5.48064	-0.43955
H	-0.18574	-5.69189	0.12604
H	-1.9435	-5.6408	0.24322
H	-1.17929	-6.22756	-1.23352
C	-2.41726	-3.8191	-1.83591
H	-2.39725	-4.42395	-2.75035
H	-3.30827	-4.10871	-1.26482
H	-2.51855	-2.77129	-2.125
H	3.20223	1.60844	0.13377
H	1.76148	0.81817	0.79203
C	2.80269	1.52057	-2.81461
H	3.48086	0.66532	-2.67968
C	2.35841	1.50786	-4.28474
H	1.68091	2.33587	-4.51325
H	3.24054	1.60961	-4.93081
H	1.84803	0.57776	-4.53681
C	3.59334	2.80453	-2.5076
H	2.98626	3.70039	-2.67374
H	3.97636	2.84204	-1.48265
H	4.45858	2.87047	-3.18012
C	0.41717	2.59594	-1.15463
H	-0.43999	2.1419	-0.64361
C	-0.12256	3.30971	-2.4059
H	-0.87886	4.04599	-2.10485
H	0.6684	3.85438	-2.93445
H	-0.58049	2.6031	-3.10052
C	1.06434	3.6016	-0.18649
H	1.94486	4.09065	-0.61594
H	0.33572	4.38866	0.04695
H	1.35734	3.14408	0.76356
C	6.39231	-2.34185	0.70129
H	7.05499	-2.2473	-0.16911
H	6.74554	-1.63334	1.45773
H	6.51347	-3.35565	1.09597
C	0.2055	-1.52634	1.89706
C	-1.46521	-1.2467	-5.39867
C	-1.63094	0.27813	-5.05925
B	-0.43654	-0.79145	-3.35124
O	-1.04237	-1.7941	-4.12728
O	-0.69789	0.44637	-3.96025
C	-1.24186	1.24089	-6.1828
H	-1.88005	1.09484	-7.0626
H	-1.37088	2.2746	-5.84449
H	-0.19982	1.11393	-6.48477
C	-0.34454	-1.52327	-6.41402
H	-0.16668	-2.60235	-6.46726
H	-0.60832	-1.17253	-7.41794
H	0.59156	-1.04347	-6.11235
C	-3.03336	0.63482	-4.54354
H	-3.01915	1.65365	-4.14284
H	-3.77917	0.59489	-5.34536
H	-3.34946	-0.03807	-3.74146
C	-2.74384	-1.96191	-5.83752
H	-3.13595	-1.53458	-6.76827

H	-2.52955	-3.02062	-6.01973
H	-3.5214	-1.9046	-5.07258
C	-0.87674	-0.63108	-0.30857
C	-2.01282	0.04891	-0.82344
C	-0.90031	-0.83762	1.10727
C	-3.08857	0.50226	-0.05702
H	-2.04942	0.27379	-1.88233
C	-1.98314	-0.34328	1.84728
C	-3.08384	0.30854	1.32242
H	-3.92363	1.01356	-0.53276
H	-3.88115	0.64598	1.97676
H	0.82178	-0.7711	2.4153
F	-1.96307	-0.51486	3.21019
H	0.87732	-2.00583	1.18709
Si	-0.24073	-2.80286	3.25884
H	-1.62044	-3.33828	3.06814
C	0.95241	-4.28382	3.14196
H	1.99858	-3.95505	3.08905
H	0.8552	-4.92675	4.02576
H	0.75337	-4.91178	2.26562
C	-0.0389	-2.08636	5.00116
H	1.01137	-1.83219	5.1952
H	-0.63727	-1.18109	5.13232
H	-0.34564	-2.81393	5.76245
H	1.45075	-1.19934	-2.98596

Thermal correction to Energy=0.967881

Thermal correction to Enthalpy=0.968825

Thermal correction to Gibbs Free Energy=0.825019

Sum of electronic and zero-point Energies=-2817.427049

Sum of electronic and thermal Energies=-2817.371843

Sum of electronic and thermal Enthalpies=-2817.370899

Sum of electronic and thermal Free Energies= -2817.514706

SCF Done: E(wB97XD) = -2819.35700947

Int2a'

Number of Negative Frequencies =o

B	1.95968	1.29045	0.92402
B	2.24414	-0.07458	-1.50546
B	1.99215	-1.46249	0.8638
C	-1.07938	-0.7201	2.46361
H	-0.25954	-1.40177	2.65636
C	-2.20792	-0.70597	3.27894
C	-3.27102	0.15402	2.98296
C	-3.09917	0.98415	1.86777
C	-1.93412	0.93585	1.0979
C	-1.72438	1.79534	-0.09685
C	-2.57775	2.853	-0.42263
C	-2.36355	3.63608	-1.56506
C	-1.25157	3.29514	-2.34229
C	-0.42523	2.24002	-1.95988
H	0.45874	1.96814	-2.52854
C	3.68259	2.70166	1.54486
C	2.46587	2.87801	2.5316
C	4.16891	-0.47348	-2.74662
C	3.19737	0.44358	-3.5751
C	2.52704	-3.53385	1.80861
C	3.77237	-2.57117	1.87142
Ir	0.82454	-0.06981	-0.04235
N	-0.93562	0.06833	1.39103
N	-0.64554	1.50003	-0.86304
O	3.08236	1.97027	0.4501
O	1.63744	1.73918	2.21015
O	3.29876	-0.97431	-1.70951
O	2.24364	0.8521	-2.57165
O	1.63301	-2.82858	0.91844
O	3.18166	-1.28743	1.58243
H	-0.13714	-1.49519	-1.07896
C	-2.68999	-1.55934	-2.06693
C	4.26726	4.00442	0.99695
H	4.68385	4.62018	1.8035
H	5.07707	3.77633	0.2956
H	3.51642	4.59124	0.46202
C	4.80544	1.82229	2.11526
H	5.52659	1.61222	1.31852
H	5.336	2.31835	2.93642
H	4.4091	0.86476	2.46213
C	2.8208	2.81774	4.01799
H	3.49551	3.63572	4.29896
H	1.90853	2.91174	4.6175
H	3.29605	1.86846	4.27521
C	1.63033	4.13685	2.24736
H	0.70767	4.08865	2.83564
H	2.16334	5.05522	2.51869
H	1.35456	4.19374	1.1895
C	4.46566	-2.4961	3.23393
H	5.29643	-1.78427	3.18145
H	3.78432	-2.15664	4.01829
H	4.87618	-3.47109	3.52416

C	4.8143	-2.85361	0.77797
H	5.5544	-2.04661	0.78488
H	5.33768	-3.80203	0.9469
H	4.34872	-2.86379	-0.21034
C	1.80655	-3.67902	3.15901
H	0.84972	-4.18877	3.00106
H	2.39086	-4.26644	3.87627
H	1.60397	-2.6988	3.60314
C	2.80597	-4.91962	1.2236
H	3.53006	-5.47051	1.83629
H	1.87855	-5.50278	1.19385
H	3.19339	-4.84985	0.20467
C	2.42001	-0.32105	-4.65903
H	1.62246	0.32246	-5.04637
H	3.06193	-0.61177	-5.49836
H	1.95779	-1.22305	-4.24533
C	3.83852	1.69361	-4.18009
H	4.62558	1.42871	-4.89676
H	3.08048	2.27693	-4.71536
H	4.27027	2.3324	-3.4064
C	4.755	-1.65988	-3.51412
H	5.37345	-1.32131	-4.35451
H	5.39087	-2.25046	-2.84602
H	3.97289	-2.31862	-3.90022
C	5.29419	0.31099	-2.05321
H	5.80954	-0.35838	-1.35684
H	6.02954	0.69501	-2.77003
H	4.88485	1.14306	-1.47344
Si	-1.17766	-2.54076	-1.408
C	-0.42568	-3.61443	-2.76648
H	-0.10774	-3.00911	-3.62332
H	0.45659	-4.13977	-2.38385
H	-1.13942	-4.36609	-3.12596
C	-1.61561	-3.55529	0.11599
H	-2.27323	-3.00164	0.79356
H	-2.12539	-4.48469	-0.16493
H	-0.69099	-3.80031	0.64689
H	-1.00075	3.84138	-3.24379
H	-2.243	-1.38367	4.12308
C	-4.57592	0.20068	3.7916
H	-3.4121	3.07926	0.23003
H	-3.8966	1.65861	1.58184
C	-3.31407	4.79439	-1.90363
C	-2.89984	5.52347	-3.19599

H	-3.6056	6.33565	-3.40194
H	-1.90125	5.96684	-3.11214
H	-2.90625	4.85316	-4.06298
C	-4.74663	4.24052	-2.09385
H	-4.7829	3.51439	-2.91383
H	-5.11697	3.74396	-1.19069
H	-5.43852	5.057	-2.33209
C	-3.30591	5.81752	-0.74253
H	-2.30153	6.22647	-0.58575
H	-3.98142	6.65095	-0.96927
H	-3.63591	5.36971	0.2008
C	-5.76199	-0.13452	2.85442
H	-6.69693	-0.15714	3.42688
H	-5.87708	0.61087	2.06021
H	-5.62377	-1.10781	2.3728
C	-4.76567	1.61948	4.37905
H	-5.70256	1.67049	4.94639
H	-3.94371	1.88012	5.05536
H	-4.80975	2.38335	3.59529
C	-4.57216	-0.81071	4.95346
H	-4.47099	-1.84176	4.5961
H	-3.76358	-0.61248	5.66625
H	-5.51803	-0.7429	5.50197
H	-2.83083	-0.69826	-1.40543
H	-2.43219	-1.16336	-3.05701
C	-3.95799	-2.36671	-2.14416
C	-4.3372	-3.08181	-3.29115
C	-4.82259	-2.46025	-1.04799
C	-5.50903	-3.84004	-3.33059
H	-3.69846	-3.03027	-4.16979
C	-5.99629	-3.20074	-1.05498
C	-6.34239	-3.90114	-2.21232
H	-5.77197	-4.37835	-4.23681
H	-6.61765	-3.22	-0.16524
H	-7.2566	-4.48706	-2.23604
F	-4.49598	-1.78307	0.08918

Thermal correction to Energy=1.197289

Thermal correction to Enthalpy=1.198233

Thermal correction to Gibbs Free Energy=1.022041

Sum of electronic and zero-point Energies=-2887.499097

Sum of electronic and thermal Energies=-2887.431028

Sum of electronic and thermal Enthalpies=-2887.430084

Sum of electronic and thermal Free Energies=-2887.606276

SCF Done: E(wB97XD) = -2888.62639662

Int2a'

Number of Negative Frequencies =o

B	-1.45693	-1.96292	1.17297
B	-2.47576	-0.71864	-1.18306
B	-2.15182	0.72082	1.08046
C	1.11404	0.42527	2.3823
H	0.17304	0.80901	2.76006
C	2.29488	0.56271	3.10708
C	3.48697	0.02793	2.60697
C	3.39719	-0.64164	1.37884
C	2.18241	-0.7461	0.6971
C	2.03703	-1.46419	-0.59781
C	3.1055	-2.09284	-1.24147
C	2.92805	-2.77296	-2.45408
C	1.63109	-2.7695	-2.97838
C	0.60448	-2.11998	-2.29632
H	-0.40696	-2.08209	-2.68806
C	-2.67552	-3.6617	2.13766
C	-1.24057	-3.6084	2.78147
C	-4.59748	-1.18912	-2.00235
C	-3.63314	-0.88734	-3.20787
C	-3.26199	2.68958	1.64547
C	-3.63442	1.54861	2.66303
Ir	-0.86119	-0.43667	0.02949
N	1.05094	-0.19742	1.19892
N	0.79	-1.48793	-1.12895
O	-2.50421	-2.85275	0.95069
O	-0.75057	-2.3277	2.32654
O	-3.83898	-0.70327	-0.8775
O	-2.33168	-0.94333	-2.5755
O	-2.6317	1.9465	0.57988
O	-2.61559	0.55525	2.39446
H	-1.03617	2.29432	-0.95514
C	-0.04827	2.2813	-1.4081
C	0.20023	1.66998	-2.64186
C	1.4839	1.71257	-3.19194
H	1.6827	1.23903	-4.14958
C	2.51723	2.36186	-2.51361
H	3.51444	2.39277	-2.94762
C	2.30708	2.97756	-1.26904
C	1.00775	2.91162	-0.75929
C	4.83652	0.14179	3.33259
C	4.11839	-3.46271	-3.1387
C	-3.15124	-5.05237	1.71749
H	-3.2376	-5.71807	2.58503
H	-4.13915	-4.97635	1.25106
H	-2.47294	-5.50978	0.99281
C	-3.74699	-2.97614	2.99931
H	-4.66199	-2.87659	2.40658
H	-3.9797	-3.55439	3.90119
H	-3.42607	-1.97134	3.28835
C	-1.21015	-3.62708	4.31037
H	-1.64045	-4.55613	4.70385
H	-0.1734	-3.56142	4.65875
H	-1.76	-2.78135	4.72944
C	-0.28827	-4.68056	2.22727
H	0.73074	-4.45341	2.55823
H	-0.54848	-5.68477	2.5808
H	-0.29618	-4.68402	1.13252
C	-3.55629	1.942	4.13868
H	-3.78742	1.07253	4.76342
H	-2.55855	2.2954	4.41171
H	-4.28162	2.72978	4.376
C	-4.99273	0.89553	2.36354
H	-5.11125	0.01484	3.00289
H	-5.8269	1.57786	2.56351
H	-5.04057	0.56203	1.32292
C	-2.2207	3.67908	2.19277
H	-1.84889	4.29767	1.37016
H	-2.64513	4.33765	2.95921
H	-1.36201	3.15257	2.62081
C	-4.45108	3.45719	1.0656
H	-5.00943	3.97732	1.85354
H	-4.09239	4.20904	0.35398
H	-5.13423	2.79045	0.53421
C	-3.80048	0.53135	-3.77418
H	-2.9827	0.73656	-4.47375
H	-4.74665	0.64776	-4.31467
H	-3.76027	1.27859	-2.97564
C	-3.66965	-1.90794	-4.34622
H	-4.65878	-1.93692	-4.81909
H	-2.93872	-1.63143	-5.11455
H	-3.42581	-2.91289	-3.99266
C	-5.93762	-0.45223	-2.04549
H	-6.52373	-0.74368	-2.92583
H	-6.52167	-0.70461	-1.15389
H	-5.80027	0.63176	-2.06018

C	-4.83815	-2.69028	-1.77565
H	-5.34835	-2.82065	-0.81648
H	-5.46012	-3.12955	-2.56445
H	-3.89241	-3.23612	-1.71819
H	-0.60609	1.14647	-3.14505
H	1.39563	-3.26461	-3.91275
H	2.26068	1.08516	4.05526
H	4.09082	-2.05684	-0.79365
H	4.28768	-1.08678	0.95234
C	4.71771	0.91648	4.65864
H	4.03579	0.42272	5.35999
H	5.70021	0.9745	5.13962
H	4.36587	1.94253	4.50202
C	5.84289	0.88563	2.42172
H	6.00994	0.35624	1.47754
H	5.48601	1.89362	2.18161
H	6.81152	0.97845	2.927
C	5.37228	-1.27651	3.64317
H	6.34039	-1.21061	4.15365
H	4.68115	-1.82417	4.29357
H	5.51628	-1.86923	2.73347
C	4.73171	-4.50639	-2.17471
H	5.58381	-5.00455	-2.65203
H	5.09219	-4.04814	-1.24762
H	3.99674	-5.27331	-1.90587
C	3.70199	-4.18591	-4.4335
H	3.29282	-3.49186	-5.17623
H	4.57699	-4.66935	-4.88161
H	2.95537	-4.96497	-4.24252
C	5.18459	-2.39853	-3.49437
H	4.77659	-1.64642	-4.17903
H	5.55354	-1.87618	-2.60515
H	6.04399	-2.87272	-3.98314
F	0.77119	3.49467	0.44813
C	3.39513	3.70635	-0.52269
H	3.26293	3.57715	0.55852
H	4.37463	3.28249	-0.77882
Si	3.45844	5.5846	-0.88933
H	3.89561	5.7501	-2.30931
C	1.76483	6.39125	-0.67143
H	1.82177	7.47125	-0.85238
H	1.03746	5.97206	-1.37526
H	1.37256	6.23409	0.33928
C	4.74901	6.38973	0.24015
H	4.85387	7.4589	0.02057
H	4.46634	6.2948	1.29582
H	5.73583	5.92793	0.11579

Thermal correction to Energy=1.197480

Thermal correction to Enthalpy=1.198424

Thermal correction to Gibbs Free Energy= 1.019054

Sum of electronic and zero-point Energies= -2887.496631

Sum of electronic and thermal Energies= -2887.428210

Sum of electronic and thermal Enthalpies= -2887.427266

Sum of electronic and thermal Free Energies= -2887.606636

SCF Done: E(wB97XD) = -2888.62736521

Int2c'

Number of Negative Frequencies =o

B	-1.22201	-1.72648	0.81416
B	-1.98906	-0.39311	-1.54775
B	-1.69466	0.78411	1.07665
C	1.66812	0.82568	2.05415
H	0.80451	1.42213	2.32497
C	2.89076	0.96973	2.70684
C	3.99554	0.21695	2.30038
C	3.77214	-0.69323	1.25944
C	2.51743	-0.80649	0.65699
C	2.23872	-1.76383	-0.44012
C	3.14167	-2.76461	-0.81131
C	2.84888	-3.67265	-1.83568
C	1.60095	-3.52002	-2.45064
C	0.73211	-2.51601	-2.03552
H	-0.2497	-2.39328	-2.47932
C	-2.45261	-3.58918	1.45207
C	-1.289	-3.3396	2.48353
C	-4.13277	-0.31042	-2.4201
C	-3.2437	-1.23391	-3.33822
C	-2.27904	2.04156	2.94715
C	-3.56792	1.47968	2.24421
Ir	-0.40998	-0.12584	-0.26506
N	1.47814	-0.0325	1.04531
N	1.03824	-1.64446	-1.06452
O	-2.07869	-2.72224	0.35127
O	-0.84339	-2.00705	2.13206
O	-3.14793	0.3721	-1.617
O	-2.04315	-1.39168	-2.54115
O	-1.26907	1.81379	1.9319
O	-3.01777	0.4633	1.3738

H	-0.51479	1.4718	-0.83866
C	0.73701	1.70941	-1.42475
C	5.39879	0.37723	2.90265
C	3.85951	-4.7627	-2.22359
C	-2.55906	-5.02073	0.92451
H	-2.79277	-5.72334	1.73359
H	-3.36486	-5.07835	0.18524
H	-1.63536	-5.34331	0.43802
C	-3.82388	-3.11652	1.95547
H	-4.549	-3.19303	1.1391
H	-4.18436	-3.72883	2.78976
H	-3.78506	-2.06936	2.26601
C	-1.70814	-3.34332	3.95387
H	-2.10127	-4.32329	4.25024
H	-0.83974	-3.12298	4.58403
H	-2.47116	-2.58804	4.15617
C	-0.08668	-4.27712	2.28686
H	0.74485	-3.91915	2.90285
H	-0.3169	-5.30644	2.58415
H	0.24379	-4.2816	1.24331
C	-4.5874	0.82957	3.17918
H	-5.41978	0.42793	2.5919
H	-4.14716	0.00681	3.7475
H	-4.9952	1.56185	3.88648
C	-4.2688	2.51021	1.34574
H	-5.01932	1.99518	0.73883
H	-4.76787	3.29018	1.93189
H	-3.55944	2.98327	0.66049
C	-1.86587	1.23741	4.18899
H	-0.87848	1.57546	4.52205
H	-2.56827	1.37972	5.01784
H	-1.79596	0.16974	3.96039
C	-2.31035	3.53428	3.27419
H	-3.10702	3.76216	3.99267
H	-1.35638	3.83485	3.72048
H	-2.46409	4.13943	2.37801
C	-2.84614	-0.5717	-4.66479
H	-2.09203	-1.18916	-5.16408
H	-3.70591	-0.47499	-5.33736
H	-2.41742	0.42059	-4.5017
C	-3.81833	-2.62556	-3.61334
H	-4.77088	-2.56062	-4.15292
H	-3.11932	-3.19548	-4.2359
H	-3.97625	-3.18307	-2.68762

C	-4.96675	0.73463	-3.16294
H	-5.69426	0.25759	-3.83072
H	-5.52099	1.34007	-2.43791
H	-4.33948	1.40858	-3.75106
C	-5.03071	-1.10175	-1.45583
H	-5.47306	-0.40582	-0.73636
H	-5.8407	-1.61936	-1.98298
H	-4.44487	-1.8323	-0.8919
H	1.27718	-4.17985	-3.24911
H	2.9625	1.69669	3.50654
H	4.08004	-2.85192	-0.27774
H	4.59523	-1.29415	0.89309
C	5.4248	1.41754	4.03832
H	4.75954	1.13816	4.86332
H	6.43964	1.49374	4.44393
H	5.13483	2.41345	3.68525
C	6.3624	0.84753	1.78555
H	6.45697	0.10251	0.98809
H	6.00631	1.77733	1.32857
H	7.36309	1.02397	2.19776
C	5.88255	-0.97751	3.47144
H	6.89162	-0.87307	3.88749
H	5.22027	-1.32848	4.27104
H	5.92049	-1.75588	2.70173
C	4.16071	-5.64614	-0.98899
H	4.88383	-6.42664	-1.25322
H	4.58571	-5.06658	-0.16265
H	3.25115	-6.13529	-0.62272
C	3.32882	-5.66915	-3.35037
H	3.12542	-5.1049	-4.26744
H	4.07799	-6.43112	-3.5914
H	2.41061	-6.18994	-3.05619
C	5.16717	-4.09276	-2.70954
H	4.98425	-3.46173	-3.58648
H	5.61813	-3.46548	-1.93325
H	5.90136	-4.85801	-2.98821
C	1.08785	2.9248	-0.63623
C	0.14542	3.9362	-0.35964
C	2.38101	3.16137	-0.15838
C	0.48589	5.09756	0.32496
H	-0.8802	3.78641	-0.68991
C	2.75192	4.30841	0.53295
C	1.7942	5.29231	0.77695
H	-0.27279	5.85299	0.51462

H	3.78202	4.41632	0.85922
H	2.06766	6.19658	1.31337
Si	0.25724	2.04971	-3.2265
H	-1.19467	2.3833	-3.32513
C	1.21907	3.52985	-3.92577
H	1.02735	4.44405	-3.35336
H	2.30093	3.34775	-3.90603
H	0.9318	3.71539	-4.96796
C	0.63561	0.52674	-4.28338
H	0.07538	-0.34417	-3.93284
H	0.37613	0.6985	-5.33168
H	1.70596	0.2878	-4.23884
F	3.35231	2.23087	-0.38676
H	1.59688	1.03521	-1.45426

Thermal correction to Energy= 1.197574

Thermal correction to Enthalpy= 1.198518

Thermal correction to Gibbs Free Energy= 1.019544

Sum of electronic and zero-point Energies= -2887.494130

Sum of electronic and thermal Energies= -2887.425672

Sum of electronic and thermal Enthalpies= -2887.424727

Sum of electronic and thermal Free Energies=-2887.603702

SCF Done: E(RwB97XD) = -2888.61296423

TS2c'

Number of Negative Frequencies =1

B	-1.1573	-1.62141	0.81453
B	-2.05429	-0.37176	-1.56861
B	-1.73405	0.88794	1.00422
C	1.60882	0.93839	1.99594
H	0.77429	1.60466	2.18276
C	2.76298	0.97534	2.77601
C	3.82058	0.10312	2.50271
C	3.6357	-0.77029	1.42297
C	2.45579	-0.75591	0.67437
C	2.22222	-1.66711	-0.47511
C	3.1663	-2.60685	-0.8993
C	2.90815	-3.4748	-1.96762
C	1.65118	-3.35167	-2.56856
C	0.74672	-2.39818	-2.10951
H	-0.24032	-2.29157	-2.5465
C	-2.27578	-3.5225	1.53281
C	-1.11677	-3.17344	2.54029
C	-4.23777	-0.44687	-2.33373
C	-3.32894	-1.30704	-3.29351
C	-2.33065	2.21898	2.81394
C	-3.60131	1.51672	2.21483
Ir	-0.4424	0.00038	-0.35175
N	1.44805	0.09248	0.9729
N	1.02178	-1.55873	-1.09878
O	-1.9622	-2.67571	0.3973
O	-0.74024	-1.83674	2.13208
O	-3.26785	0.31006	-1.58146
O	-2.08275	-1.374	-2.55445
O	-1.3532	2.00423	1.76419
O	-3.01444	0.49252	1.3794
H	-0.80303	1.52825	-0.8189
C	0.63115	1.55265	-1.56665
C	5.12868	0.07481	3.3081
C	3.96764	-4.49372	-2.41447
C	-2.30957	-4.97575	1.05765
H	-2.49423	-5.65969	1.89506
H	-3.12043	-5.10425	0.333
H	-1.37581	-5.26578	0.56971
C	-3.66564	-3.10795	2.0372
H	-4.39664	-3.26419	1.23769
H	-3.97809	-3.70125	2.90402
H	-3.68365	-2.04708	2.29885
C	-1.52157	-3.13731	4.01431
H	-1.85982	-4.12279	4.35662
H	-0.6598	-2.84527	4.62423
H	-2.32112	-2.41464	4.1933
C	0.12759	-4.05912	2.36856
H	0.94607	-3.63933	2.96276
H	-0.05061	-5.08629	2.70632
H	0.44965	-4.08786	1.32284
C	-4.52084	0.85098	3.2386
H	-5.35282	0.36236	2.72071
H	-3.99554	0.09088	3.82215
H	-4.94176	1.59112	3.9298
C	-4.42408	2.43237	1.29527
H	-5.16128	1.82618	0.7608
H	-4.95309	3.2077	1.861
H	-3.78859	2.91239	0.54544
C	-1.79768	1.52894	4.07944
H	-0.82434	1.95966	4.33867
H	-2.46747	1.6723	4.93469
H	-1.65883	0.45651	3.91204

C	-2.46258	3.72292	3.05312
H	-3.22991	3.93573	3.80728
H	-1.51052	4.12294	3.41819
H	-2.72064	4.25615	2.13526
C	-3.0477	-0.62336	-4.63896
H	-2.28206	-1.18956	-5.17944
H	-3.9472	-0.58719	-5.264
H	-2.67943	0.39651	-4.49999
C	-3.8132	-2.7373	-3.54168
H	-4.79188	-2.74219	-4.0366
H	-3.10498	-3.25706	-4.19695
H	-3.88751	-3.3025	-2.61014
C	-5.18136	0.53035	-3.03622
H	-5.89849	-0.00043	-3.67406
H	-5.74841	1.09284	-2.28669
H	-4.6318	1.24851	-3.64876
C	-5.02323	-1.29811	-1.32448
H	-5.47717	-0.63403	-0.58233
H	-5.8183	-1.87719	-1.80863
H	-4.35259	-1.97938	-0.79502
H	1.35204	-3.98396	-3.39559
H	2.81334	1.69448	3.58446
H	4.119	-2.66709	-0.38913
H	4.42546	-1.46399	1.16372
C	5.13027	1.11365	4.44536
H	4.32446	0.93148	5.1653
H	6.0788	1.06	4.99094
H	5.02594	2.13559	4.06389
C	6.31541	0.38409	2.3641
H	6.39819	-0.34906	1.55463
H	6.20644	1.37409	1.90751
H	7.25761	0.36851	2.92483
C	5.31573	-1.3297	3.93026
H	6.25009	-1.36705	4.50284
H	4.49064	-1.57209	4.60949
H	5.36222	-2.11364	3.16677
C	4.28862	-5.44606	-1.23752
H	5.0491	-6.17527	-1.54066
H	4.67455	-4.90598	-0.36648
H	3.39583	-5.99744	-0.92168
C	3.48795	-5.34116	-3.60808
H	3.26738	-4.7222	-4.48505
H	4.27246	-6.05049	-3.89344
H	2.59186	-5.92229	-3.36268
C	5.25313	-3.74151	-2.83534
H	5.05571	-3.0626	-3.67238
H	5.66944	-3.14842	-2.01411
H	6.02093	-4.45755	-3.15116
C	1.22277	2.72339	-0.82479
C	0.45801	3.79378	-0.32466
C	2.60803	2.84649	-0.64823
C	1.04255	4.90244	0.28981
H	-0.62401	3.73776	-0.40209
C	3.22241	3.93223	-0.03737
C	2.42901	4.9779	0.43592
H	0.4111	5.70579	0.65973
H	4.30455	3.94572	0.05043
H	2.89076	5.8378	0.9134
Si	0.0603	2.05245	-3.31832
H	-1.3981	2.37694	-3.33739
C	0.98621	3.57976	-3.95848
H	0.78287	4.46438	-3.34488
H	2.07208	3.42168	-3.95699
H	0.68306	3.80642	-4.98869
C	0.40373	0.61013	-4.49868
H	-0.09594	-0.30219	-4.16359
H	0.06264	0.83073	-5.51758
H	1.48326	0.41263	-4.54486
F	3.41719	1.84688	-1.10393
H	1.46187	0.87499	-1.79065

Thermal correction to Energy=1.192691

Thermal correction to Enthalpy=1.193635

Thermal correction to Gibbs Free Energy=1.022833

Sum of electronic and zero-point Energies=-2887.444584

Sum of electronic and thermal Energies= -2887.377361

Sum of electronic and thermal Enthalpies= -2887.376416

Sum of electronic and thermal Free Energies=-2887.547218

SCF Done: E(RwB97XD) = -2888.58003577

Int3c'

Number of Negative Frequencies =0

Ir	0.21356	0.69179	-0.20647
B	1.83304	1.08018	-1.35755
B	1.3996	-0.84821	0.33511
O	1.63115	1.76972	-2.57376
O	3.09261	0.4733	-1.37887
C	-4.16453	1.45161	0.11952
C	-3.29283	3.34295	1.26958

C	-2.02446	2.94103	0.86456
H	-1.14992	3.51195	1.15907
C	-3.51378	-0.72951	-1.84189
N	-1.24719	-0.51682	-1.03147
N	-1.79805	1.85963	0.10613
O	1.98783	-0.92537	1.5942
O	1.62682	-2.05254	-0.35906
C	-2.54784	-0.08467	-1.06859
C	-2.86197	1.11457	-0.26698
C	-3.22001	-1.87182	-2.59372
C	-4.4135	2.5874	0.90005
C	2.19821	-3.02239	0.55475
C	2.83589	-2.09496	1.65543
C	2.43282	1.09984	-3.57105
C	3.63667	0.50761	-2.72023
C	-1.89624	-2.31954	-2.50281
C	1.53271	0.02195	-4.19955
H	1.19308	-0.69733	-3.44799
H	0.64794	0.50928	-4.62007
H	2.04312	-0.52397	-5.00081
C	2.83004	2.10825	-4.64814
H	3.44295	1.63355	-5.42359
H	1.92836	2.50341	-5.12864
H	3.38984	2.95206	-4.23847
C	4.89666	1.38088	-2.73189
H	5.59575	1.00236	-1.97914
H	5.38858	1.3394	-3.71077
H	4.68151	2.41988	-2.48649
C	4.02331	-0.92769	-3.08989
H	4.33989	-0.99953	-4.13842
H	4.86233	-1.25094	-2.46484
H	3.19631	-1.62116	-2.9182
C	3.1892	-3.89666	-0.21322
H	3.69099	-4.60312	0.45891
H	2.65876	-4.47772	-0.97624
H	3.9481	-3.2926	-0.71446
C	1.04184	-3.88554	1.08498
H	0.31559	-3.27564	1.63165
H	0.52551	-4.35223	0.23883
H	1.39655	-4.68227	1.74832
C	2.78927	-2.64914	3.08008
H	3.37631	-3.57199	3.16397
H	3.21603	-1.91216	3.76804
H	1.76548	-2.8554	3.40232

C	4.26406	-1.64286	1.31623
H	4.97794	-2.47295	1.37396
H	4.30266	-1.19957	0.31778
H	4.56925	-0.8738	2.03204
C	-4.29759	-2.55619	-3.44211
C	-5.84734	2.93983	1.32369
C	-0.96144	-1.64022	-1.73443
H	0.0574	-1.98935	-1.64504
C	1.62563	2.4524	0.33496
H	-4.51297	-0.31058	-1.87119
H	-4.98377	0.8041	-0.16653
H	-1.5582	-3.20302	-3.03549
H	-3.38412	4.23641	1.87596
C	-5.90329	4.22957	2.16391
H	-6.94254	4.44582	2.43375
H	-5.33458	4.13581	3.09599
H	-5.51946	5.09464	1.61134
C	-6.71594	3.14347	0.05893
H	-6.32896	3.96216	-0.55816
H	-6.75308	2.24283	-0.5631
H	-7.74456	3.39118	0.34578
C	-6.42796	1.78016	2.16849
H	-5.83331	1.61628	3.07412
H	-7.45423	2.01555	2.4738
H	-6.45493	0.83876	1.6096
C	-4.86513	-1.54482	-4.46692
H	-5.63836	-2.02381	-5.07912
H	-5.31965	-0.6764	-3.97802
H	-4.0785	-1.17918	-5.1362
C	-3.74162	-3.76815	-4.21352
H	-3.35419	-4.53993	-3.53889
H	-4.5401	-4.223	-4.80998
H	-2.93928	-3.47894	-4.90174
C	-5.43821	-3.04513	-2.51689
H	-5.0683	-3.7726	-1.78553
H	-5.89782	-2.21826	-1.96441
H	-6.2246	-3.5277	-3.10929
C	2.72248	2.22593	1.34989
C	2.36072	1.92129	2.67959
C	4.10087	2.35955	1.13956
C	3.28381	1.78939	3.71004
H	1.30368	1.77533	2.89105
C	5.05561	2.22836	2.14507
C	4.64753	1.94574	3.44671

H	2.94037	1.56375	4.71505
H	6.10132	2.36645	1.88743
H	5.38215	1.8494	4.24083
Si	1.64919	4.17549	-0.53002
H	1.49378	5.11432	0.63949
C	0.13477	4.44455	-1.64251
H	-0.81981	4.2771	-1.13754
H	0.13968	5.48223	-2.00624
H	0.18692	3.77335	-2.50278
C	3.15227	4.83978	-1.47076
H	3.33984	4.27827	-2.38848
H	2.93262	5.88012	-1.75104
H	4.06264	4.83025	-0.87017
F	4.5755	2.66959	-0.09388
H	0.76029	2.70401	0.97721

Thermal correction to Energy=0.992319

Thermal correction to Enthalpy=0.993263

Thermal correction to Gibbs Free Energy= 0.844998

Sum of electronic and zero-point Energies= -2475.784797

Sum of electronic and thermal Energies= -2475.728632

Sum of electronic and thermal Enthalpies=-2475.727688

Sum of electronic and thermal Free Energies=-2475.875953

SCF Done: E(RwB97XD) = -2476.65589626

TS4c'

Number of Negative Frequencies =1

Ir	0.21057	0.71882	-0.22056
B	1.94865	1.11789	-1.39038
B	1.38986	-0.82536	0.31105
O	1.72622	1.69264	-2.6563
O	3.19705	0.50318	-1.36238
C	-4.17078	1.45774	0.17842
C	-3.28761	3.29674	1.39917
C	-2.02196	2.89539	0.98427
H	-1.14374	3.45174	1.2984
C	-3.53707	-0.69112	-1.82329
N	-1.26226	-0.4952	-1.02238
N	-1.80157	1.8413	0.18892
O	1.95034	-0.91819	1.58159
O	1.64037	-2.01673	-0.39731
C	-2.56479	-0.06208	-1.04389
C	-2.87196	1.11699	-0.21366
C	-3.2517	-1.82406	-2.59134
C	-4.41435	2.56786	0.99649
C	2.18945	-2.9981	0.51571
C	2.7975	-2.08798	1.64763
C	2.58331	1.01004	-3.60021
C	3.77918	0.50343	-2.69078
C	-1.93061	-2.28282	-2.50645
C	1.75216	-0.13067	-4.21418
H	1.40919	-0.82797	-3.4456
H	0.86796	0.2991	-4.69636
H	2.32	-0.68879	-4.96692
C	2.97723	1.99014	-4.70678
H	3.63272	1.50852	-5.44191
H	2.07663	2.33018	-5.22912
H	3.49038	2.8697	-4.31296
C	5.00258	1.42863	-2.70066
H	5.68965	1.11802	-1.90832
H	5.53002	1.36603	-3.65969
H	4.73508	2.46851	-2.51366
C	4.2423	-0.92773	-2.98947
H	4.60257	-1.02119	-4.02104
H	5.07096	-1.18722	-2.32194
H	3.44039	-1.64927	-2.8224
C	3.19986	-3.86037	-0.24055
H	3.68408	-4.57849	0.43222
H	2.68874	-4.42814	-1.02643
H	3.97224	-3.2495	-0.71202
C	1.02042	-3.87017	1.00168
H	0.27632	-3.26835	1.53326
H	0.53066	-4.32865	0.13559
H	1.35756	-4.67327	1.66666
C	2.71364	-2.66569	3.06124
H	3.29905	-3.58947	3.14615
H	3.12109	-1.93976	3.77234
H	1.68168	-2.87775	3.35222
C	4.23418	-1.6289	1.35551
H	4.94764	-2.459	1.41905
H	4.30065	-1.16863	0.36625
H	4.51754	-0.87189	2.093
C	-4.3356	-2.48488	-3.4539
C	-5.84676	2.92308	1.42289
C	-0.98966	-1.61612	-1.73471
H	0.02844	-1.97201	-1.65413
C	1.59186	2.40507	0.23019
H	-4.53335	-0.26478	-1.84343
H	-4.9964	0.83124	-0.1377

H	-1.60394	-3.16567	-3.04334
H	-3.37253	4.16706	2.03807
C	-5.89618	4.19592	2.28904
H	-6.93452	4.41199	2.56262
H	-5.32845	4.07984	3.21906
H	-5.50733	5.06988	1.75418
C	-6.71079	3.15792	0.16046
H	-6.31669	3.98622	-0.43899
H	-6.75225	2.27005	-0.47923
H	-7.73845	3.40666	0.44992
C	-6.43734	1.75016	2.24237
H	-5.84718	1.5646	3.14678
H	-7.46352	1.98502	2.54861
H	-6.46685	0.8204	1.66448
C	-4.86657	-1.45709	-4.48227
H	-5.64475	-1.91413	-5.10521
H	-5.30325	-0.57838	-3.99514
H	-4.06317	-1.10935	-5.14128
C	-3.79504	-3.70578	-4.22207
H	-3.41572	-4.47858	-3.5439
H	-4.59954	-4.15394	-4.81574
H	-2.9909	-3.42843	-4.91267
C	-5.49928	-2.95396	-2.54742
H	-5.15233	-3.6866	-1.81001
H	-5.95545	-2.11962	-2.00363
H	-6.28334	-3.42507	-3.15203
C	2.65537	2.23761	1.29768
C	2.2879	1.94142	2.62618
C	4.02871	2.41442	1.1002
C	3.21169	1.83644	3.66182
H	1.23538	1.7698	2.83528
C	4.98332	2.30643	2.10629
C	4.57389	2.01751	3.40615
H	2.86678	1.60937	4.66698
H	6.02656	2.46461	1.85067
H	5.30733	1.93855	4.20375
Si	1.5591	4.13915	-0.61771
H	1.41142	5.07644	0.54864
C	0.02168	4.33896	-1.70735
H	-0.91739	4.11573	-1.1914
H	-0.03375	5.37242	-2.0734
H	0.10211	3.67032	-2.56945
C	3.02826	4.80639	-1.60543
H	3.23415	4.18305	-2.47898
H	2.76472	5.81104	-1.96176
H	3.94065	4.88226	-1.01056
F	4.48574	2.73078	-0.13869
H	0.71056	2.65447	0.84602

Thermal correction to Energy= 0.990728

Thermal correction to Enthalpy=0.991672

Thermal correction to Gibbs Free Energy=0.846973

Sum of electronic and zero-point Energies=-2475.735146

Sum of electronic and thermal Energies= -2475.679750

Sum of electronic and thermal Enthalpies=-2475.678806

Sum of electronic and thermal Free Energies= -2475.823504

SCF Done: E(RwB97XD) = -2476.61093105

Int5c'

Number of Negative Frequencies =0

Ir	0.18206	0.67289	-0.20017
B	1.92754	1.28178	-1.23785
B	1.3996	-0.85451	0.32881
O	1.65635	1.82012	-2.54226
O	3.11781	0.5111	-1.34737
C	-4.15823	1.45161	0.11952
C	-3.29283	3.34295	1.26958
C	-2.02446	2.94733	0.85196
H	-1.14992	3.52455	1.14017
C	-3.50748	-0.72951	-1.84189
N	-1.23459	-0.51052	-1.03777
N	-1.79805	1.87853	0.08093
O	1.98783	-0.92537	1.5879
O	1.62682	-2.05884	-0.35906
C	-2.54154	-0.07837	-1.07489
C	-2.86197	1.12087	-0.27958
C	-3.21371	-1.87182	-2.59372
C	-4.4135	2.5874	0.90005
C	2.19821	-3.02239	0.55475
C	2.83589	-2.09496	1.65543
C	2.42022	1.11244	-3.53325
C	3.62407	0.51391	-2.69503
C	-1.88994	-2.31954	-2.50281
C	1.51381	0.03455	-4.15545
H	1.17418	-0.67213	-3.39759
H	0.62904	0.52188	-4.58227
H	2.01792	-0.51767	-4.95671
C	2.82374	2.10195	-4.63554
H	3.43034	1.60835	-5.40469

H	1.92206	2.49711	-5.11604
H	3.38984	2.94576	-4.23847
C	4.89666	1.37458	-2.73819
H	5.60205	1.00236	-1.99174
H	5.37598	1.3268	-3.72337
H	4.69411	2.41988	-2.49909
C	4.00441	-0.92769	-3.07099
H	4.31469	-0.99323	-4.11952
H	4.84973	-1.25094	-2.45224
H	3.17741	-1.61486	-2.893
C	3.1892	-3.89666	-0.21322
H	3.69099	-4.60312	0.45891
H	2.65876	-4.47772	-0.97624
H	3.9481	-3.2926	-0.71446
C	1.04184	-3.88554	1.08498
H	0.31559	-3.27564	1.63165
H	0.52551	-4.35223	0.23883
H	1.39655	-4.68227	1.74832
C	2.78927	-2.64914	3.08008
H	3.37631	-3.57199	3.16397
H	3.21603	-1.91216	3.76804
H	1.76548	-2.8554	3.40232
C	4.26406	-1.64286	1.31623
H	4.97794	-2.47295	1.37396
H	4.30266	-1.19957	0.31778
H	4.56925	-0.8738	2.03204
C	-4.29759	-2.55619	-3.44211
C	-5.84734	2.93983	1.32369
C	-0.95514	-1.64022	-1.73443
H	0.0637	-1.99565	-1.64504
C	1.78313	2.3768	0.15226
H	-4.50667	-0.31058	-1.86489
H	-4.97747	0.7978	-0.16023
H	-1.5582	-3.20302	-3.02919
H	-3.38412	4.23011	1.88226
C	-5.90329	4.22957	2.16391
H	-6.94254	4.44582	2.43375
H	-5.33458	4.13581	3.09599
H	-5.51946	5.09464	1.61134
C	-6.71594	3.14347	0.05893
H	-6.32896	3.96216	-0.55816
H	-6.75308	2.24283	-0.5631
H	-7.74456	3.39118	0.34578
C	-6.42796	1.78016	2.16849

H	-5.83331	1.61628	3.07412
H	-7.45423	2.01555	2.4738
H	-6.45493	0.83876	1.6096
C	-4.86513	-1.54482	-4.46692
H	-5.63836	-2.02381	-5.07912
H	-5.31965	-0.6764	-3.97802
H	-4.0785	-1.17918	-5.1362
C	-3.74162	-3.76815	-4.21352
H	-3.35419	-4.53993	-3.53889
H	-4.5401	-4.223	-4.80998
H	-2.93928	-3.47894	-4.90174
C	-5.43821	-3.04513	-2.51689
H	-5.0683	-3.7726	-1.78553
H	-5.89782	-2.21826	-1.96441
H	-6.2246	-3.5277	-3.10929
C	2.76658	2.20703	1.29949
C	2.35442	1.92759	2.61659
C	4.14497	2.36585	1.13326
C	3.25861	1.80199	3.67224
H	1.29738	1.76903	2.80285
C	5.07451	2.24096	2.15767
C	4.62863	1.95204	3.44671
H	2.88997	1.57635	4.67095
H	6.12652	2.37275	1.92523
H	5.34435	1.8557	4.25973
Si	1.65549	4.18179	-0.53632
H	1.48118	5.03242	0.68989
C	0.14107	4.44455	-1.63621
H	-0.81351	4.2645	-1.13124
H	0.13968	5.48223	-1.98734
H	0.19952	3.77965	-2.50278
C	3.15227	4.86498	-1.45816
H	3.32724	4.30347	-2.38218
H	2.94522	5.90532	-1.73214
H	4.06894	4.84285	-0.86387
F	4.6196	2.67589	-0.10018
H	0.79179	2.37641	0.68741

Thermal correction to Energy=0.991853

Thermal correction to Enthalpy=0.992798

Thermal correction to Gibbs Free Energy=0.847047

Sum of electronic and zero-point Energies=-2475.744393

Sum of electronic and thermal Energies= -2475.688680

Sum of electronic and thermal Enthalpies= -2475.687736

Sum of electronic and thermal Free Energies=-2475.833486

SCF Done: E(RwB97XD) = -2476.61919338

TS2c

Number of Negative Frequencies =1

B	-0.20341	1.07238	-2.01027
C	0.70121	0.24491	2.62379
C	0.64001	0.0035	3.99461
C	-0.58135	-0.28458	4.61216
C	-1.719	-0.25819	3.79939
C	-1.61062	0.01096	2.43583
C	-0.91861	1.78218	-4.11942
C	0.59791	2.14837	-3.93184
N	-0.40937	0.20817	1.83662
O	-1.15136	0.8636	-3.02614
O	0.78077	1.95073	-2.50941
C	1.55054	1.19061	-4.66306
H	2.57748	1.40058	-4.34597
H	1.33216	0.14912	-4.41803
H	1.49702	1.3196	-5.75018
C	0.96728	3.59381	-4.27302
H	2.03244	3.75616	-4.07507
H	0.78841	3.80726	-5.33384
H	0.40142	4.31146	-3.67409
C	-1.25702	1.07424	-5.43285
H	-2.32636	0.83896	-5.46163
H	-1.03076	1.71318	-6.29509
H	-0.70326	0.13842	-5.53702
C	-1.85826	2.98337	-3.92223
H	-1.76702	3.71123	-4.73632
H	-2.89378	2.62961	-3.89912
H	-1.65324	3.49377	-2.97572
H	1.5563	0.0375	4.57756
H	-2.70365	-0.41992	4.22947
H	0.25293	-0.465	-1.35209
C	-2.82012	0.22133	1.57181
C	1.98187	0.60428	1.92275
H	2.69391	1.06632	2.61481
C	-0.6669	-0.60582	6.08258
H	-0.51457	-1.6795	6.25633
H	0.09971	-0.07263	6.65462
H	-1.64727	-0.34447	6.49401
Co	-0.22123	0.32337	-0.17683
H	2.44475	-0.30263	1.51158
P	1.49584	1.6244	0.42558
C	0.95653	3.29364	1.15933
H	-0.09265	3.08293	1.39924
C	0.95974	4.38638	0.07691
H	0.429	5.2761	0.43938
H	1.97815	4.69758	-0.18354
H	0.46968	4.03979	-0.83799
C	1.63456	3.7671	2.45585
H	2.70218	3.96924	2.32934
H	1.16481	4.70057	2.79426
H	1.52273	3.03742	3.26468
C	3.12659	1.93636	-0.46061
H	2.82948	2.58417	-1.29145
C	4.22137	2.638	0.35912
H	5.13028	2.7323	-0.24974
H	3.93476	3.6453	0.67237
H	4.49323	2.06782	1.25582
C	3.66192	0.63125	-1.06845
H	4.54707	0.8476	-1.68069
H	3.97363	-0.07857	-0.29214
H	2.92098	0.14203	-1.70081
H	-3.06188	1.29162	1.62169
H	-3.68895	-0.31689	1.96308
P	-2.43828	-0.13139	-0.22986
C	-3.34903	-1.75199	-0.61687
C	-3.8082	0.85883	-1.11498
C	-5.07389	1.16344	-0.29258
H	-4.86709	1.73278	0.61865
H	-5.76063	1.76884	-0.89887
H	-5.61067	0.25237	-0.0072
C	-3.07002	-2.25711	-2.04294
H	-3.62445	-1.68607	-2.79331
H	-2.0064	-2.20118	-2.28443
H	-3.37981	-3.30643	-2.12972
C	-4.20713	0.26252	-2.47619
H	-4.82165	-0.63616	-2.35945
H	-4.81248	0.99486	-3.0273
H	-3.3319	0.02184	-3.07996
H	-3.29477	1.80661	-1.31641
C	-4.85042	-1.82156	-0.29176
H	-5.44664	-1.2072	-0.97445
H	-5.20218	-2.85644	-0.39826
H	-5.08099	-1.50573	0.7315
H	-2.82647	-2.43859	0.06389
C	0.76572	-1.74954	-0.53782

H	1.68632	-1.51545	-0.04527
C	1.09465	-2.72792	-1.68069
C	0.65711	-4.05101	-1.61386
C	1.82988	-2.29136	-2.78269
C	0.95535	-4.93744	-2.64851
H	0.07814	-4.39507	-0.74457
C	2.12746	-3.17768	-3.81821
H	2.17466	-1.24855	-2.83562
C	1.69046	-4.50059	-3.75123
H	0.61105	-5.98049	-2.59561
H	2.70682	-2.83308	-4.68715
H	1.92544	-5.19968	-4.56693
Si	-0.05196	-2.58625	1.00973
H	-1.34805	-2.07745	1.48106
C	1.31672	-2.99547	2.32231
H	0.92386	-2.84474	3.3061
H	1.62256	-4.01495	2.21272
H	2.15862	-2.35241	2.17206
C	-0.93756	-4.22465	0.46662
H	-0.25108	-5.04217	0.53942
H	-1.77648	-4.40536	1.10572
H	-1.27357	-4.13006	-0.54484

Thermal correction to Energy= 0.980931

Thermal correction to Enthalpy=0.991876

Thermal correction to Gibbs Free Energy= 0.808314

Sum of electronic and zero-point Energies=-2819.456638

Sum of electronic and thermal Energies=-2819.362003

Sum of electronic and thermal Enthalpies=-2819.361059

Sum of electronic and thermal Free Energies= -2819.504620

SCF Done: E(wB97XD) = -2819.303233
